# Functional annotation of the human brain methylome identifies tissue-specific epigenetic variation across brain and blood

**DOI:** 10.1186/gb-2012-13-6-r43

**Published:** 2012-06-15

**Authors:** Matthew N Davies, Manuela Volta, Ruth Pidsley, Katie Lunnon, Abhishek Dixit, Simon Lovestone, Cristian Coarfa, R Alan Harris, Aleksandar Milosavljevic, Claire Troakes, Safa Al-Sarraj, Richard Dobson, Leonard C Schalkwyk, Jonathan Mill

**Affiliations:** 1 Institute of Psychiatry, King's College London, De Crespigny Park, London, SE5 8AF, UK; 2 Department of Twin Research and Genetic Epidemiology, King's College London, Westminster Bridge Road, London, SE1 7EH, UK; 3 Department of Molecular and Human Genetics, Baylor College of Medicine, One Baylor Plaza, Houston, TX 77030, USA

## Abstract

**Background:**

Dynamic changes to the epigenome play a critical role in establishing and maintaining cellular phenotype during differentiation, but little is known about the normal methylomic differences that occur between functionally distinct areas of the brain. We characterized intra- and inter-individual methylomic variation across whole blood and multiple regions of the brain from multiple donors.

**Results:**

Distinct tissue-specific patterns of DNA methylation were identified, with a highly significant over-representation of tissue-specific differentially methylated regions (TS-DMRs) observed at intragenic CpG islands and low CG density promoters. A large proportion of TS-DMRs were located near genes that are differentially expressed across brain regions. TS-DMRs were significantly enriched near genes involved in functional pathways related to neurodevelopment and neuronal differentiation, including *BDNF*, *BMP4*, *CACNA1A*, *CACA1AF*, *EOMES*, *NGFR*, *NUMBL*, *PCDH9*, *SLIT1*, *SLITRK1 *and *SHANK3*. Although between-tissue variation in DNA methylation was found to greatly exceed between-individual differences within any one tissue, we found that some inter-individual variation was reflected across brain and blood, indicating that peripheral tissues may have some utility in epidemiological studies of complex neurobiological phenotypes.

**Conclusions:**

This study reinforces the importance of DNA methylation in regulating cellular phenotype across tissues, and highlights genomic patterns of epigenetic variation across functionally distinct regions of the brain, providing a resource for the epigenetics and neuroscience research communities.

## Background

DNA methylation is a key epigenetic mechanism involved in the developmental regulation of gene expression [1], but the tissue-specific nature of DNA methylation has not been fully characterized at a genomic level. Epigenetic processes control several neurobiological and cognitive processes, including neurogenesis and brain development [2], neuronal activity [3], learning and memory [4], drug addiction [5], neurodegeneration [6], and circadian rhythm [7]. The importance of DNA methylation in normal brain function and development is exemplified by the neurodevelopmental deficits associated with mutations in the methyl CpG binding protein 2 gene (*MECP2*) in Rett syndrome [8], and the aberrant DNA methylation signatures observed in neuropsychiatric disorders, including schizophrenia and bipolar disorder [9]. Although gene expression analyses have highlighted clear transcriptomic differences across brain regions [10-12], current studies of tissue-specific DNA methylation in the brain have assessed only a small percentage of CpG sites in the human genome [13], and none has taken an unbiased methylome-wide approach across multiple brain regions and blood obtained from the same individuals. Little is known, therefore, about normal methylomic differences between functionally distinct areas of the brain and how these correspond to patterns observed in easily accessible peripheral tissues such as blood. In this study we used methylated DNA immunoprecipitation combined with ultra-deep sequencing (MeDIP-seq) to profile the methylomic landscape across multiple dissected brain regions and blood obtained from multiple individuals. We present annotated maps of the brain methylome, representing a unique resource for the genomics and neuroscience research communities, identifying key regions of the genome characterized by functionally relevant tissue-specific DNA methylation.

## Results and discussion

### Methylomic profiling across brain and blood

Our primary methylomic profiling experiments used multiple dissected brain regions (inferior frontal gyrus, middle frontal gyrus, entorhinal cortex, superior temporal gyrus of the temporal cortex, visual cortex, and cerebellum) from post-mortem brain samples obtained from individuals free of any neuropathology and neuropsychiatric disease. From a subset of these individuals, whole blood samples were also obtained longitudinally prior to death. A detailed list of the primary samples used in this study is given in Supplementary Table 1 in Additional file 1. Of these, 21 tissue samples from three individuals (two female, one male) were initially assessed using ultra-deep paired-end MeDIP-seq (see Materials and methods). After stringent quality control (Supplementary Figure 1 in Additional file 1), an average of >70.4 million uniquely mapped 50 bp reads were obtained from each of the 21 samples (Supplementary Table 2 in Additional file 1); to our knowledge, this represents the largest between-individual and cross-tissue DNA methylation dataset yet produced. To generate an estimate of actual DNA methylation from our MeDIP-seq data, we used the MEDIPS analysis package [14] to control for local CpG density and generate DNA methylation scores for overlapping 500 bp bins across the genome. Bisulfite pyrosequencing was used to verify DNA methylation estimates at selected regions of the genome, and examine base pair-specific levels of DNA methylation across nominated regions in additional brain and blood samples. Normalized raw MeDIP-seq reads and MEDIPS-estimated absolute DNA methylation values for each tissue/individual combination are available as a resource for download and browsing as UCSC tracks from our laboratory website [15]. The data are also being integrated into the Human Epigenome Atlas [16,17] as part of the regular data release by NIH Epigenomics Roadmap Initiative [18].

### Genome-wide DNA methylation across cortex, cerebellum and peripheral blood is highly tissue-specific

**Figure 1:**
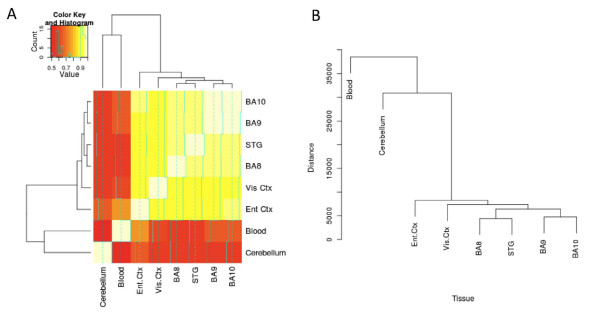
**Methylomic profiling across multiple brain areas and blood from a cohort of individuals highlights clear tissue-specific differences in DNA methylation**. **(a, b) **DNA methylation was calculated from ultra-deep MeDIP-seq data using 500 bp bins across the genome, and the relationship between tissues determined by Pearson correlation (a) and unsupervised hierarchical clustering (b). BA, Brodmann area; Ent Ctx, entorhinal cortex; STG, superior temporal gyrus; Vis Ctx, visual cortex.

As expected, canonical genic DNA methylation profiles do not differ across samples or tissue types, with overall low average DNA methylation around the immediate transcription start site, high levels of DNA methylation across the gene body, and more subtle hypomethylation being observed at the 3' end of genes (Supplementary Figure 2 in Additional file 1), confirming previous observations [19]. Genome-wide, however, there are striking tissue-specific differences in DNA methylation, with a clear hierarchical distinction between the six cortical regions, cerebellum and blood (Figure 1a, b). These broad differences reflect the known developmental pathways of the three tissues; blood cells originate from the mesoderm, while cells of the central nervous system are ectodermic. Within the brain, the cerebellum develops from the metencephalon, whilst the cerebral cortex develops from the most anterior part of the neural plate (the telencephalon). The cortex itself is subdivided into numerous functionally distinct anatomical regions, specializing in sensory, motor, and association tasks.

### DNA methylation varies across different functionally annotated regions of the genome

**Figure 2:**
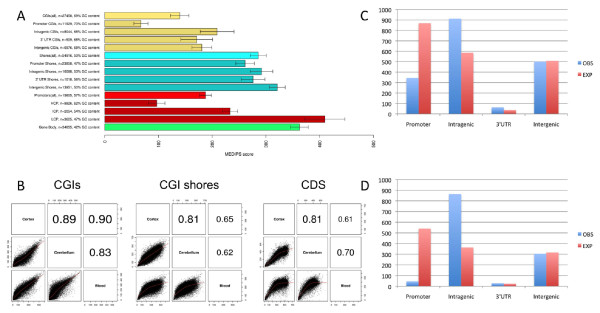
**Although DNA methylation at CGIs is relatively conserved across tissues, intragenic CGIs are dramatically over-represented and promoter CGIs under-represented in the most tissue-variable CGIs**. **(a) **Average DNA methylation values calculated by MEDIPS from MeDIP-seq data for all annotated gene features: CGIs (yellow), CGI shores (blue), gene promoters (red) and CDSs (green). DNA methylation is lower in promoter CGIs compared to intragenic, 3' UTR and intergenic CGIs. CGI shores are characterized by higher DNA methylation than CGIs, with less location-dependent variation. Promoter DNA methylation shows a strong inverse correlation with GC density, with LCPs showing a higher average level of DNA methylation than CDSs. Error bars represent standard error of the mean. **(b) **Although TS-DMRs are distributed across all feature types, there are marked differences in the between-tissue correlation of DNA methylation across each of the broad feature categories we examined, with CGIs being more correlated across cortex, cerebellum and blood than CGI shores or CDSs. **(c, d) **There is a highly significant enrichment of intragenic CGIs (*P *= 2 × 10^-102^) in analyses of CGI DMRs differentiating blood, cortex and cerebellum (c), and an even more dramatic enrichment (*P *= 1 × 10^-246^) in comparisons between cortex and cerebellum (d). EXP, expected; OBS, observed.

We defined feature sets spanning i) all annotated CpG islands (CGIs), sub-typed by location (gene promoter, intragenic, 3' UTR, and intergenic) as described previously [19], ii) CGI shores (spanning 2,000 bp up- and downstream of each CGI), and iii) all annotated coding sequences (CDSs). We also examined tissue-specific patterns of DNA methylation across gene promoters defined by low, medium and high CG content (LCPs, ICPs, and HCPs, respectively) [20]. BED files of the feature annotations used in this study (CGIs, CGI shores, CDSs, LCPs, ICPs, and HCPs) are available for download from our laboratory webpage [15]. DNA methylation across each feature was quantified using both normalized MeDIP-seq read counts and MEDIPS scores; Figure 2a shows the average DNA methylation levels for each feature type. As described previously [21], CGIs are significantly hypomethylated compared to CGI shores and the gene body, with no overall difference in canonical methylation patterns between tissues or individuals. There is, however, considerable heterogeneity in DNA methylation across different categories of CGI, dependent upon genomic location, with promoter CGIs being significantly hypomethylated in comparison to intragenic, 3' UTR, and intergenic CGIs. CGI shores, on the other hand, do not vary significantly as a function of genomic location. Although the majority of promoters (approximately 60%) are associated with CGIs, not all promoter regions are hypomethylated; promoter methylation is inversely correlated with CG density, with LCPs being the most methylated of any feature type tested. In contrast, HCPs (which overlap considerably with promoter CGIs) are hypomethylated relative to the other features.

### DNA methylation differs significantly between tissues across all annotated feature types

We next examined tissue- and brain region-specific DNA methylation in the context of annotated gene features to identify regions of the genome harboring tissue-specific differentially methylated regions (TS-DMRs). Due to the overall similarity between the six cortical regions, we initially focused on gross differences between cortex, cerebellum and blood. Cross-tissue normalized MeDIP-seq data for all 65,535 annotated features can be downloaded from our laboratory website [15]. For each broad category of feature, hierarchical clustering of the MeDIP-seq data can clearly distinguish between tissue types (Supplementary Figure 3 in Additional file 1). The top 50 variably methylated annotated features across cortex, cerebellum and blood are listed in Supplementary Table 3 in Additional file 1. Using Illumina 450 K HumanMethylation microarray data obtained from matched cerebellum, frontal cortex, and whole blood samples from 90 individuals being assessed by our group as part of an ongoing clinical epigenetics study (Mill *et al*., unpublished data), we were able to confirm that the vast majority of these features are true TS-DMRs (Supplementary Table 4 in Additional file 1); 74% of the 206 probes mapping to the broad genomic regions covered by these features were characterized by false discovery rate-significant intra-individual between-tissue DNA methylation differences in the direction predicted by our MeDIP-seq data.

Ingenuity Pathway Analysis (IPA) of the most variable features (defined as those with a coefficient of variance (CV) >1 across the three tissue types) highlights a highly significant enrichment of functional pathways involved in regulating developmental gene expression (*P *= 8.83 × 10^-25^), organismal development (*P *= 1.37 × 10^-20^), and tissue differentiation (*P *= 2.34 × 10^-20^) (Supplementary Figure 4 in Additional file 1). Of note, given the origins of the samples used in this analysis, it is interesting that the primary tissue-specific functional pathways enriched in the list of TS-DMRs are nervous system development and function (*P *= 4.60 × 10^-21^) and hematological system development and function (*P *= 3.61 × 10^-8^), indicating that epigenetic differences are likely to be associated with significant phenotypic differences. Although TS-DMRs are distributed across all feature types, there are marked differences in the between-tissue correlation of DNA methylation across each of the broad feature categories we examined, with CGIs being more correlated across cortex, cerebellum and blood than CGI shores or CDSs (Figure 2b).

### TS-DMRs across brain regions are associated with stable gene expression differences and are highly enriched for functionally relevant neurobiological pathways

Within the brain, the top 50 differentially methylated annotated features between cortical and cerebellum samples are listed in Supplementary Table 5 in Additional file 1. Strikingly, many of these DMRs are associated with genes of known relevance to cortical and/or cerebellar development and function, including *PPP2R2B *(encoding a phosphatase implicated in the negative control of cell growth and division, whose disruption causes cerebellar ataxia [22]), *JAKMIP1/MARLIN1 *(encoding a RNA-binding protein that associates with GABA receptors [23]), *EOMES *(encoding a transcriptional activator that plays a crucial role in brain development, controlling the proliferation of intermediate progenitor cells and their progeny in the cerebral cortex [24]), *GPMB6 *(encoding a proteolipid widely expressed in neurons and in oligodendrocytes [25]), and *GRM4 *(encoding a metabotropic glutamate receptor with a distinct distribution in the brain, primarily expressed in cerebellar granule cells [26]). Furthermore, IPA of the top within-brain variable DMRs (differentiating cortex from cerebellum) highlights a primary network involved in nervous system development and function (Supplementary Figure 5 in Additional file 1), with highly significant enrichment of developmentally relevant pathways, including neurogenesis (*P *= 1.76 × 10^-19^), the guidance of neurites (*P *= 2.11 × 10^-12^), development of the cerebellum (*P *= 8.49 × 10^-5^) and development of the cortex (*P *= 1.11 × 10^-4^) (Supplementary Table 6 in Additional file 1).

We next used RNA extracted from matched cerebellum and frontal cortex (Brodmann area (BA)9) samples, obtained from an independent cohort of 42 additional individuals, to assess tissue-specific expression levels for detectable gene transcripts located in the vicinity of the top 50 cortex-cerebellum DMRs. The majority (82%) of these DMRs are mirrored by significant (*P *< 0.05) gene expression differences between cerebellum and frontal cortex (Supplementary Table 7 in Additional file 1), with 61% of genes being represented by at least one probe with a highly significant (*P *< 1 × 10^-10^) expression difference across the two brain regions. Interestingly, DNA methylation at these DMRs is not always negatively correlated with gene expression; *EOMES*, for example, shows highly significant elevated expression in the cerebellum compared to the frontal cortex (log2 expression values = 9.25 versus 6.46, *P *= 1.90 × 10^-33^). An analysis of publically available gene expression data [12] confirms many of these gene expression differences and demonstrates that many of the cortex-cerebellum DMRs are associated with developmentally stable gene expression differences between the brain regions (Supplementary Figure 6 in Additional file 1). This further supports the notion that the TS-DMRs identified here mediate functionally important differences in the cellular transcriptome.

### DNA methylation differs across functionally discrete regions of the cerebral cortex

Although for all features tested the six cortical regions formed a tight cluster, distinct from both cerebellum and blood (Figure 1a, b), we were interested to see if we could identify DMRs that could distinguish between them. Supplementary Table 8 in Additional file 1 lists the 50 most variably methylated features across samples obtained from the frontal cortex (BA8, BA9, and BA10), entorhinal cortex, superior temporal gyrus, and visual cortex. While the magnitude of within-cortex variation is clearly lower than observed between average cortex and either cerebellum or blood, there are some noticeable region-specific patterns of DNA methylation, particularly in the visual cortex. This list of DMRs contains a striking number of genes implicated in brain function related to the cortex and neurodevelopment, including *CACNA1A *and *CACNA1F *(calcium-channel genes involved in neuronal growth and development and controlling the release of neurotransmitters [27]), *GALNT9 *(a brain-specific O-glycosylase [28]), *SLC8A2/NCX2 *(a sodium/calcium exchanger that has been shown to be important in synaptic plasticity and cortical development [29]), *NUMBL *(encoding a protein that maintains progenitor cells during cortical neurogenesis [30]), and *GRIK5 *(a receptor for the excitory neurotansmitter glutamate [31]). IPA on loci associated with the top 500 across-cortex variably methylated features highlights an interactive network of genes involved in neurodevelopment and function (Supplementary Figure 7 in Additional file 1), with a significant enrichment for functional pathways associated with neurogenesis and neuronal function, including many directly related to development of the cortex, such as 'forebrain development' (*P *= 6.15 × 10^-7^) (Supplementary Table 9 in Additional file 1).

### Although DNA methylation at promoter CGIs is strongly conserved across brain areas and blood, tissue-specific DNA methylation is particularly striking at intragenic CGIs

**Figure 3:**
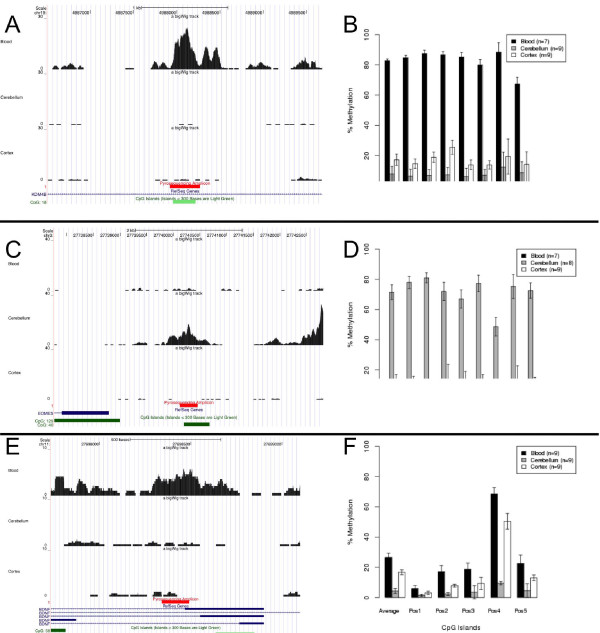
**Verification and replication of MeDIP-seq data for three top-ranked CGI DMRs**. **(a, b) **Tissue-specific DNA methylation across an intragenic CGI in the *JMJD2B*/*KDM4B *gene. (a) MeDIP-seq analysis shows this region is hypermethylated in blood DNA compared to cortex and cerebellum (the red bar depicts the region subsequently analyzed by bisulfite pyrosequencing). (b) Pyrosequencing data for this region in an extended sample set confirm significant tissue-specific methylation patterns (*P *= 2 × 10^-8^). **(c, d) **Tissue-specific DNA methylation across a CGI in the promoter of the *EOMES *gene. (c) MeDIP-seq analysis shows this region is hypermethylated in cerebellum DNA compared to cortex and blood. (d) Pyrosequencing data for this region in an extended sample set confirm significant tissue-specific methylation patterns (*P *= 2 × 10^-5^). **(e, f) **Tissue-specific DNA methylation across an intragenic CGI in the *BDNF *gene. (e) MeDIP-seq analysis shows this region is hypermethylated in blood DNA compared to cortex and cerebellum from the same individuals. (f) Pyrosequencing data for this region in an extended sample set confirm significant tissue-specific methylation patterns (*P *= 4 × 10^-9^). Error bars represent standard error of the mean.

The top 50 differentially methylated CGIs between cortex, cerebellum and blood are listed in Supplementary Table 10 in Additional file 1. Of particular interest is the observation that a CGI associated with *BMP4*, which encodes a protein mediating differentiation of the ectoderm into the nervous plate [32], is one of the strongest CGI DMRs between blood and brain (cortex and cerebellum). Also showing highly variable patterns of DNA methylation between brain tissues and blood are CGIs associated with other key neurodevelopmental genes, including *BDNF *(encoding a neurotrophic factor with an important role in neurodevelopment and neurogenesis [33]) and *SLITRK1 *(encoding an integral membrane protein involved in neurite outgrowth [34]). IPA of the most variable (CV >1) blood versus brain CGIs reveals a highly significant enrichment of functional pathways involved in basic tissue development, including loci regulating both neurogenesis (*P *= 9.88 × 10^-14^) and hematopoiesis (*P *= 4.59 × 10^-8^) (Supplementary Table 11 in Additional file 1). Bisulfite pyrosequencing was used to verify selected CGI DMRs, and examine base pair-specific levels of DNA methylation across the nominated regions in additional samples. These included the top differentially methylated CGI, located within the *JMJD2B/KDM4B *gene, encoding a histone demethylase that specifically demethylates histone H3 lysine 9, and highly ranked DMRs associated with the neurodevelopmental genes *BDNF *and *EOMES*. We observed a highly significant correlation between our MeDIP-seq and pyrosequencing data (correlation = 0.58, *P *= 7.07 × 10^-13^; Supplementary Figure 8 in Additional file 1), with highly significant between-tissue DNA methylation differences, confirming the MeDIP-seq data, being observed for all tested bisulfite-PCR amplicons (Figure 3).

**Figure 4:**
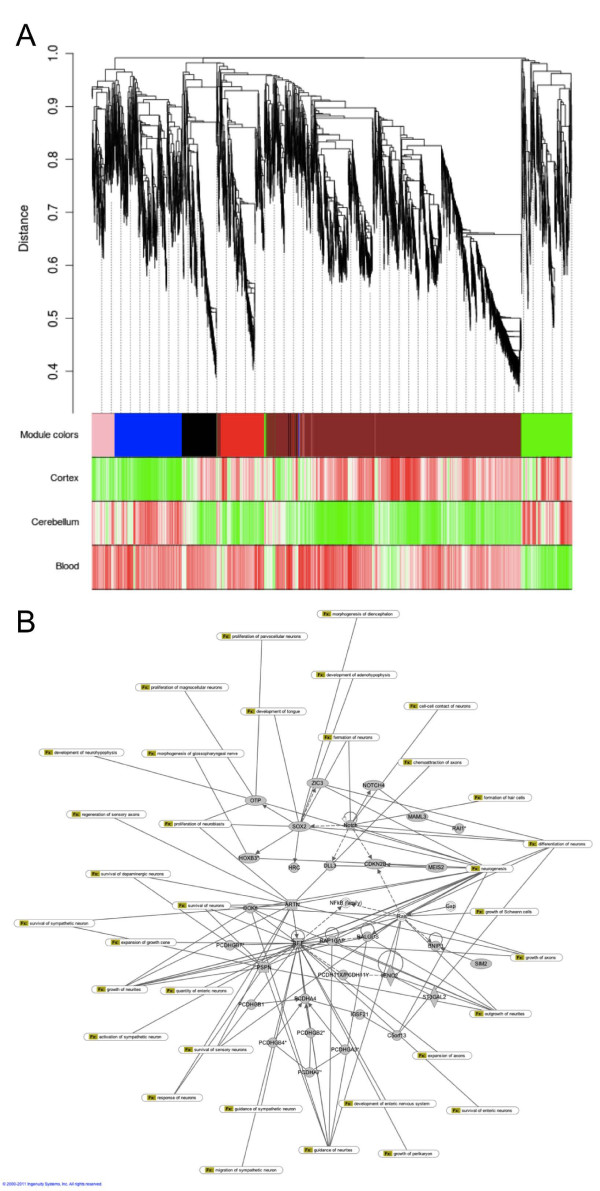
**Weighted gene co-methylation network analysis of DNA methylation at intragenic CGIs**. **(a) **Dendrograms produced by average linkage hierarchical clustering of intragenic CGIs on the basis of topological overlap. Modules of co-methylated loci were assigned colors as indicated by the horizontal bar beneath each dendrogram. The 'blue' module was strongly negatively co-methylated (r^2 ^= -0.98, *P *= 4 × 10^-5^) in cortex. **(b) **IPA on the genes associated with the blue module highlighted a network involved in nervous system development and function.

We observed a highly significant (χ^2 ^*P*-value = 2 × 10^-102^) over-representation (observed/expected (o/e) = 1.73) of intragenic CGIs amongst these TS-DMRs, with a complementary under-representation (o/e = 0.43) of promoter CGIs (Figure 2c) and a highly significant difference in average CV between these two classes of CGI (promoter CGI mean CV = 0.35, intragenic CGI mean CV = 0.60, *P *< 1 × 10^-10^). Promoter CGIs are also significantly less-variable across tissues compared to 3' CGIs (mean CV = 0.59) and intergenic CGIs (mean CV = 0.50). A comparison of the most differentially methylated CGIs between cerebellum and cortex, which again are significantly enriched for pathways related to brain development, neurogenesis and functional specialization in the brain (Supplementary Table 12 in Additional file 1), highlights an even more dramatic over-representation of intragenic CGIs (o/e = 2.37) and under-representation of promoter CGIs (o/e = 0.09) (χ^2 ^*P*-value = 1 × 10^-246^; Figure 2d). To explore the functional organization of DNA methylation at intragenic CGIs across cortex, cerebellum and blood, we used weighted gene co-methylation network analysis [35] to identify modules of co-methylated features via unsupervised hierarchical clustering on the basis of high topological overlap (see Materials and methods). Six modules were identified with clear tissue-specific patterns, demonstrating that the methylome is organized into modules of co-methylated features (Figure 4a). The strongest tissue-specific module (blue), composed of approximately 1,000 intragenic CGIs that were hypomethylated in the cortex compared to cerebellum and blood (Pearson correlation = -0.98, *P *= 4 × 10^-5^), representing a network of genes involved in nervous system development and function (Figure 4b) and significantly enriched for functional pathways, including neurogenesis (*P *= 2.47 × 10^-25^) and the differentiation of neurons (*P *= 6.18 × 10^-18^). Analysis of the top 5% of genes ranked by module membership using publically available gene expression datasets by GeneMania [36] showed that 58.9% are also coexpressed, indicating that the co-methylation networks identified here map onto functional gene coexpression networks. Furthermore, genes in this module are significantly enriched in relevant gene expression modules from published brain gene expression datasets (Supplementary Table 13 in Additional file 1). A role for differential DNA methylation at intragenic CGIs across different cell types in the mouse hematopoietic lineage has been recently reported [37], and it has been suggested that these features may regulate transcription from alternative promoters across specific cell types [19].

### On average, CGI shores show more tissue-specific DNA methylation than CGIs, but do not vary by genic location

CGI shores have been previously shown to harbor phenotypically relevant tissue-specific patterns of DNA methylation [21]. Compared to CGIs, we found CGI shores to be significantly more variable across brain areas and blood (average CGI CV = 0.46, average CGI shore CV = 0.57, *P *< 1 × 10^-10^), with DNA methylation noticeably less correlated across tissues, especially between brain (cortex and cerebellum) and blood (Figure 2b). Unlike for CGIs, however, the location of CGI shores makes little impact on their variability across tissues with no over-representation of intragenically located features (Supplementary Figure 9 in Additional file 1). The top 50 differentially methylated CGI shores between cortex, cerebellum and blood are listed in Supplementary Table 14 in Additional file 1. Strikingly, many are associated with genes of known neurobiological function, including *PCDH9 *(encoding a cadherin-related neuronal receptor that localizes to synaptic junctions, involved in specific neuronal connections and signal transduction [38]), *NGFR *(encoding the receptor for nerve growth factor, with widespread effects on neurodevelopment [39]), *AUTS2 *(encoding the autism susceptibility candidate 2, a nuclear protein expressed in developing brain regions [40]), *SHANK3 *(encoding a scaffold protein involved in the structural and functional organization of the post-synaptic density and also implicated in autism [41]), and *SLIT1 *(encoding a protein with a key role in cortical development and synaptogenesis [42]). IPA of the most variable CGI shores again reveals a highly significant enrichment for functional pathways involved in nervous system function and development (Supplementary Table 15 in Additional file 1). Bisulfite pyrosequencing was used to confirm and replicate the two top-ranked CGI shore DMRs, which are highly significantly hypermethylated specifically in the cerebellum, flanking a hypomethylated intergenic CGI on chromosome 7 (Supplementary Figure 1 in Additional file 10). Again, our pyrosequencing data were significantly correlated with the MEDIPS scores across each of the three amplicons spanning this region (left shore, correlation = 0.76, *P *= 5.58 × 10^-5^; CGI, correlation = 0.80, *P *= 1.18 × 10^-5^; right shore, correlation = 0.82, *P *= 4.36 × 10^-6^), confirming the validity of the methylome data.

### Low CG content promoters are also characterized by widespread tissue-specific DNA methylation across brain regions and blood

**Figure 5:**
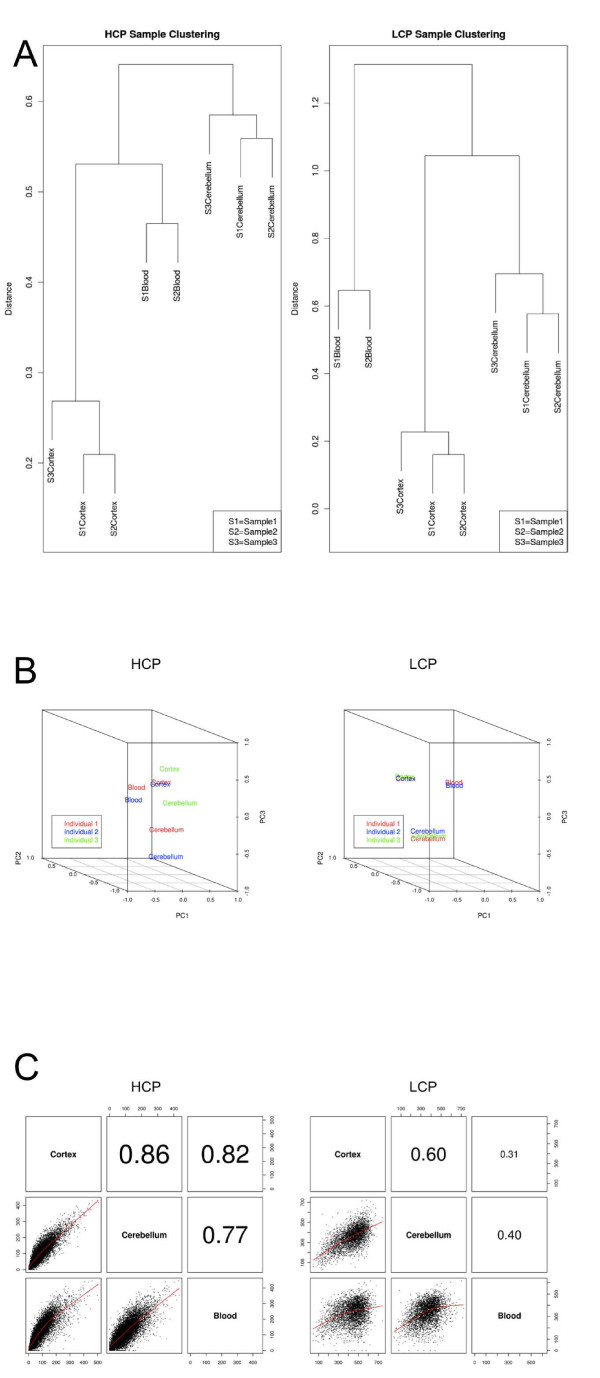
**DNA methylation across LCPs is strongly associated with tissue type**. **(a) **MEDIPS scores across both HCPs and LCPs can be used to accurately cluster samples by tissue type, but the strength of clustering, indicated by Pearson dissimilarity on the y-axis, is much higher in LCPs. **(b, c) **This pattern is reflected in three-factor PCA plots (b) and correlation analyses (c), with LCPs demonstrating stronger tissue-specific patterns of DNA methylation than HCPs.

Our CGI data concur with the notion that CpG-rich promoters (that is, HCPs) are predominantly hypomethylated and associated with ubiquitously expressed house-keeping genes [20,43]. We next decided to compare HCP methylation with that seen in LCPs; recent methylomic analyses of other tissues indicate that differential DNA methylation across LCPs is associated with tissue-specific gene expression in somatic cells [20]. Our data provide strong evidence to support this notion, and like intragenic CGIs, LCPs appear to be a major location for tissue-specific DNA methylation signatures across brain regions and blood. While hierarchical clustering of both HCP and LCP DNA methylation can distinguish between tissues, the Euclidian distance between tissues is much larger in the case of LCPs (Figure 5a). Principal components analysis of our MeDIP-seq data shows a much stronger tissue classification based upon LCP methylation (Figure 5b), and correlation analyses show that while HCP methylation is largely conserved across tissues (reflecting the pattern seen for promoter CGIs), the correlation of LCP methylation across tissues is much lower.

### Between-individual differences in DNA methylation are correlated across brain and blood

**Figure 6:**
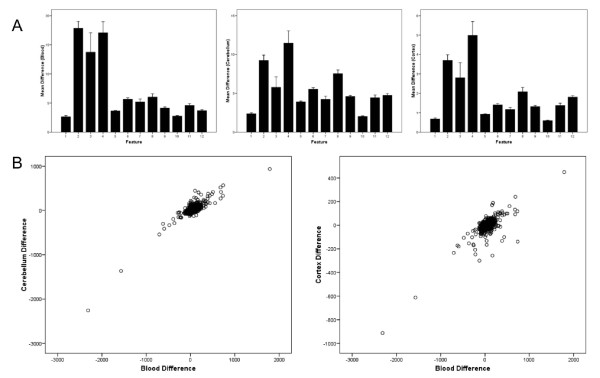
**Between-individual variation in DNA methylation is often correlated between blood and brain**. **(a) **Between-individual variation in DNA methylation is highest in blood and lowest in the cortex. All tissues show a similarly significant (ANOVA *P *< 0.001) distribution of variability across features, with the greatest between-individual variation occurring in non-promoter CGIs. Scores represent the mean difference in normalized MeDIP-seq read density between individual 1 and 2 for each of the feature categories. Error bars represent standard error of the mean. Features 1 to 4 = promoter, intragenic, 3', and intergenic CGIs; features 4 to 8 = promoter, intragenic, 3', and intergenic CGI shores; feature 9 = CDS; features 10 to 12 = HCPs, ICPs, and LCPs. **(b) **Between-individual differences in DNA methylation observed in blood are significantly (*P *< 0.001) correlated with differences observed in the cerebellum (correlation = 0.76) and cortex (correlation = 0.66) from the same individuals. Scores represent the mean difference in normalized MeDIP-seq read density between individual 1 and 2 for each of the quantified features.

Because aberrant DNA methylation is being increasingly implicated in the etiology of complex disease phenotypes, including several mental health disorders [44], a key question in epigenetic epidemiology concerns the extent to which easily accessible peripheral tissues (for example, whole blood) can be used to ask questions about inter-individual phenotypic variation manifest in inaccessible tissues such as the brain [45,46]. Our use of brain tissue and blood obtained pre-mortem from the same individuals enabled us to investigate the extent to which between-individual methylomic variation in the blood is reflected across the cortex and cerebellum. Comparing MeDIP-seq data from blood, cortex and cerebellum, we observe that inter-individual DNA methylation differences are highest in blood and lowest in the cortex, with a highly significant difference in variability across tissues (*P *< 0.001; Figure 6a). The pattern of relative inter-individual variability by feature is, however, the same across tissues, with non-promoter CGIs showing a significantly higher level of between-individual DNA methylation differences than other features. Strikingly, there is a significant correlation between individual DNA methylation differences in the blood and those in brain tissue (cortex and cerebellum) from the same two individuals (Figure 6b), with the most reproducible pattern of blood-detected individual differences observed in the cerebellum (correlation = 0.76, *P *< 0.001) and slightly less correlation between blood and cortex (correlation = 0.66, *P *< 0.001). Although replicate DNA samples from each individual were assessed using high-resolution SNP arrays to identify potential structural variants and minimize the confounding effect of inter-individual copy-number variations (CNVs) that could manifest as MeDIP-seq read differences (see Materials and methods), the influence of genomic differences between individuals cannot be fully excluded. Supplementary Table 16 in Additional file 1 lists the top 50 between-individual differences in DNA methylation identified in blood between the two female MeDIP-seq blood samples and lists the corresponding methylation scores from cortex and cerebellum from the same individuals. Interestingly, while many features show near-identical between-individual patterns across all three tissues, some examples of blood-identified variation are only detected in either the cortex or cerebellum. Given recent reports by us and others of widespread genotype-associated allele-specific DNA methylation [47], it is likely that many of these between-individual DNA methylation differences are mediated by DNA sequence variation that is common across tissues, although other mechanisms such as epigenetic changes occurring early in development before complete tissue differentiation could also be important.

## Conclusions

We used MeDIP-seq to undertake the first genomic characterization of intra- and inter-individual variation in DNA methylation across multiple regions of the human brain and an easily accessible peripheral tissue (whole blood). In summary, we show that between-tissue variation in DNA methylation greatly exceeds between-individual differences within any one tissue, with clear hierarchical differences in DNA methylation across specific brain areas, and between brain and blood. Interestingly, we observe that TS-DMRs are strikingly under-represented in classic promoter CGIs, being located primarily in intragenic CGIs and LCPs. These TS-DMRs are dramatically enriched for genes known to be involved in brain development and neurobiological function, forming networks of co-methylated loci that define the cellular phenotype. Finally, although inter-individual differences in DNA methylation are dwarfed by tissue-specific variation, and absolute levels of DNA methylation at specific loci clearly differs between cortex, cerebellum and blood, we observe that some between-individual variation in DNA methylation is correlated between brain regions and blood.

To our knowledge, this study represents the most comprehensive cross-tissue inter-individual assessment of the methylomic landscape of the brain yet undertaken. The data generated in this project are available as a resource to the genomics research community; annotated UCSC tracks can be downloaded from our laboratory website [15], and the raw data are being integrated into the Human Epigenome Atlas [16] as part of the regular data release by NIH Epigenomics Roadmap Initiative. A primary goal of global research initiatives such as the International Human Epigenome Consortium and the NIH Epigenomics Roadmap is to create high-resolution reference epigenome maps across multiple human tissue and cell types that will expedite the application of epigenomic technologies to studies of human health and disease [18]. In this regard, and given increasing evidence supporting a role for epigenetic disruption in neuropsychiatric disease, a unique aspect of this study was our use of blood and multiple brain regions from the same individuals, enabling us to address questions about the extent to which easily accessible peripheral tissues can be used to ask questions about inter-individual phenotypic variation manifest in inaccessible tissues such as the brain.

In addition to creating reference maps of DNA methylation across multiple brain regions and blood, we have identified key regions of the genome that are characterized by tissue-specific patterns of DNA methylation. Of note, TS-DMRs were significantly enriched near genes involved in functional pathways related to neurodevelopment and neuronal differentiation, including *BDNF*, *BMP4*, *CACNA1A*, *CACA1AF*, *EOMES*, *NGFR*, *NUMBL*, *PCDH9*, *SLIT1*, *SLITRK1*, and *SHANK3*. Although DNA methylation at promoter CGIs is largely conserved across tissues, reflecting data from other studies, we find striking evidence that intragenic CGIs are a primary location for TS-DMRs. This builds on recent data highlighting cell type-specific DNA methylation at intragenic CGIs in the immune system [37], and evidence that these features may regulate transcription from alternative promoters across specific cell types [19]. Our data highlight LCPs as another region characterized by considerable cross-tissue epigenetic variation; again these data support recent methylomic analyses of other tissues, indicating that differential DNA methylation across LCPs is associated with tissue-specific gene expression in somatic cells [20]. Given the striking enrichment of tissue-relevant pathways and gene sets amongst the loci associated with the TS-DMRs we have identified, and the observation that many of these genes are differentially expressed across brain regions (Supplementary Table 7 and Supplementary Figure 6 in Additional file 1), it is likely that they mediate functionally relevant differences in the cellular transcriptome.

There are several limitations to this study. First, although it represents one of the largest cross-tissue methylomic analyses performed to date, the number of individuals profiled in our initial MeDIP-seq screen was relatively small. Our successful verification and replication experiments (using bisulfite pyrosequencing and the Illumina 450 K array), combined with our gene expression data, in larger number of samples, however, highlights the validity of the between-tissue differences we observe. Second, the samples used in our initial MeDIP-seq screen were all obtained from donors >75 years old and may not fully represent patterns of DNA methylation earlier in life. However, our observation of highly significant gene expression differences corresponding to the top 50 cortex-cerebellum DMRs in a younger cohort of samples (average age = 62 ± 18 years) suggests that many of the tissue-specific differences observed are developmentally stable, resulting in functional differences at an earlier age. Future work should focus on identifying developmental trajectories of epigenetic change across multiple tissues.

In summary, this study reinforces the importance of DNA methylation in regulating cellular phenotype across tissues, and highlights genomic patterns of epigenetic variation across functionally distinct regions of the brain.

## Materials and methods

### Sample preparation

Post-mortem brain samples from nine elderly control individuals (free of neuropathological and neuropsychiatric disease) were obtained from the MRC London Neurodegenerative Diseases Brain Bank. Multiple brain regions were dissected from each sample by a trained neuropathologist, snap-frozen and stored at -80°C. Genomic DNA was isolated from each dissected brain region from each sample using a standard phenol-chloroform extraction method, and tested for degradation and purity prior to analysis. From a subset of individuals whole blood samples were also obtained longitudinally prior to death, and DNA extracted using a standard phenol-chloroform method. A detailed list of the samples used for methylomic profiling in this study is given in Supplementary Table 1 in Additional file 1. An independent set of matched frontal cortex and cerebellum samples (average age = 62 ± 18 years, 33% female) for gene expression analysis was obtained from 42 additional individuals provided by the London Neurodegenerative Diseases Brain Bank.

### Methylated DNA immunoprecipitation and sequencing

DNA was fragmented using a Covaris sonication system and sequencing libraries were prepared from 5 μg fragmented genomic DNA. End repair, <A> base addition and adaptor ligation steps were performed using Illumina's Paired-End DNA Sample Prep kit. Adaptor-ligated DNA was immunoprecipitated by anti-5mC using a commercial antibody (Diagenode, Liège, Belgium), and MeDIP products were validated by quantitative PCR. MeDIP DNA was purified with ZYMO DNA Clean & Concentrator-5 columns, and amplified using adaptor-mediated PCR. DNA fragments between 220 and 320 bp in size were gel-excised, and amplification quality and quantity were evaluated by Agilent BioAnalyzer analysis. The libraries were subjected to highly parallel 50 bp paired-end sequencing on the Illumina Hi-Seq platform.

### Sequencing quality control and alignment

From the raw fastq files, Illumina quality scores were converted into Sanger Phred quality scores using MAQ [48]. Quality control was performed on the raw sequence data using FastQC [49]. Supplementary Figure 1 in Additional file 1 shows FastQC output for one representative sample highlighting the high quality sequencing data obtained. Alignment to hg18 was performed using the Burrows-Wheeler algorithm.

### Correcting for local CpG densities

The MEDIPS package [14] was used to calculate methylation scores by incorporating a coupling factor based on local CpG density. Bin sizes of 500 bp were defined across the genome, with an overlap of 250 bp. The number of CpGs within the maximal defined distance around the genomic bin was calculated and a calibration curve determined relative to the dependency of local MeDIP-seq signal intensities and local CpG densities.

### DMR calling across known features using normalized read counts

Mapped reads were also quantified using SeqMonk (Babraham Institute, Cambridge, UK). BED files of the feature annotations used in this study (CGIs, CGI shores, CDS, LCPs, ICPs, and HCPs) are available for download from our laboratory webpage [15]. Read-depth scores were generated for each feature, normalized for total read count and feature length. For each feature, the CV across tissues/samples was calculated (Standard deviation/(Mean + 1)) and ranked.

### Weighted gene co-methylation network analysis

We employed weighted gene co-methylation network analysis [35,50,51] as described in R [52] to find weighted signed co-methylation networks (modules) in the cortex, cerebellum and peripheral blood using log transformed MEDIPS scores. For each genomic feature we performed hierarchical clustering of the samples, based on Pearson correlation, and mapped the final sample dendrogram to three traits (cortex, cerebellum and blood) to describe the sample-trait relationship. For each genomic feature type we performed hierarchical clustering of the topological overlap matrix. Leaves of the tree were grouped into modules, which is a cluster of highly co-methylated genomic locations (GL). After finding the modules, the next step was to describe the relationship between modules and each tissue (that is, cortex, cerebellum and peripheral blood). For this we calculated the Pearson correlation coefficient for the module representative also known as module eigengene (ME), which is the first principal component of each module's methylation profile, and each trait. The GL-trait relation (or gene significance GS if thinking in terms of gene expression) was defined as (the absolute value of) the correlation between the GL methylation profile and the trait. For each module, we also calculated a quantitative measure of module membership as (the absolute value of) the correlation of the ME and the GL methylation profile. This measure allows us to assess the similarity between a module's DNA methylation profile and DNA methylation at a genomic location.

### Bisulfite pyrosequencing analysis

Genomic DNA (0.5 μg), extracted from dissected brain samples and blood (Supplementary Table 1 in Additional file 1), was bisulfite converted using the EZ 96-DNA methylation kit (Zymo Research, Irvine, CA, USA) following the manufacturer's standard protocol. Fully methylated and unmethylated samples were included throughout the experimental procedure as assay controls. Pyrosequencing assays were designed using the Pyromark Assay Design Software (Qiagen, Crawley, UK). Bisulfite-PCR amplification was performed in duplicate using Hot Star Taq DNA polymerase (Qiagen, UK) and optimized cycling conditions. Pyrosequencing was performed using the Pyromark Q24 (Qiagen, UK). A full list of bisulfite PCR and sequencing primers is given in Supplementary Table 17 in Additional file 1.

### Gene expression analysis

RNA was extracted from matched cerebellum and frontal cortex samples from 42 LBBND donors using the Trizol extraction method and purified using an RNeasy Mini Kit with DNase I digestion (Qiagen, UK) according to the manufacturer's instructions. RNA was tested for purity and degradation using an Agilent 2100 Bioanalyzer and RNA 6000 Nano kit (Agilent Technologies, Wokingham, UK). RNA was biotinylated and amplified using the Illumina TotalPrep™ RNA Amplification kit (Life Technologies, Paisley, UK). Gene expression was assessed using Illumina HumanHT-12 v4 microarrays (Illumina, San Diego, California, USA) according to the standard manufacturer's protocol. Following scanning, signal intensities for each probe were extracted using Illumina GenomeStudio and imported into R using the Lumi package within Bioconductor. Probes relating to the top 50 cerebellum-cortex DMRs were tested for expression differences between cerebellum and cortex.

### Genotyping arrays and CNV analysis

For the individuals assessed by MeDIP-seq, Affymetrix SNP 6.0 arrays (Affymetrix, High Wycombe, UK) were used to genotype samples from two tissues according to the manufacturers' standard protocol. CNVs were identified using the PennCNV program [53]. Briefly, this implements a hidden Markov model that integrates multiple sources of information to infer CNV calls for individual genotyped samples. CNVs were stringently called by comparing the duplicate arrays from each individual.

### Data availability

The methylomic data generated in this project is available for browsing as UCSC Genome Browser tracks from our laboratory website [15]. Raw data have also been deposited into the Human Epigenome Atlas [16,17] and will be integrated into this resource as part of the next data release by NIH Epigenomics Roadmap Initiative.

## Abbreviations

BA: Brodmann area; bp: base pair; CDS: coding sequence; CGI: CpG island; CNV: copy number variation; CV: coefficient of variance; DMR: differentially methylated region; GL: genomic location; GS: gene significance; HCP: high CG content promoter; ICP: intermediate CG content promoter; IPA: ingenuity pathway analysis; LCP: low CG content promoter; ME: module eigengene; MeDIP-seq: methylated DNA immunoprecipitation combined with ultra-deep sequencing; o/e: observed/expected; PCR: polymerase chain reaction; SNP: single nucleotide polymorphism; TS-DMR: tissue-specific differentially methylated region; UTR: untranslated region.

## Competing interests

The authors declare that they have no competing interests.

## Authors' contributions

JM and LS conceived the experiment and participated in analysis and bioinformatics. SL provided samples. CT dissected post-mortem brain samples. MV, RP, and KL performed laboratory work. MD performed MeDIP-seq data analysis and bioinformatics. The NIH Roadmap Epigenomics Data Analysis and Coordination Center (CC, RAH, AM) provided bioinformatics support. AD and RD ran the weighted gene co-methylation network analysis. JM and MD drafted the manuscript. All authors read and approved the final manuscript.

## Supplementary Material

Additional file 1 **Supplementary figures and tables**. This file contains Supplementary Figures 1 to 10 and Supplementary Tables 1 to 17. Click here for file
