# Supplementary material for: Functional annotation of the human brain methylome identifies tissue-specific epigenetic variation across brain and blood
Source: Genome Biol. 2012 Jun 15;13(6):R43. doi: 10.1186/gb-2012-13-6-r43 (PMC3446315; doi:10.1186/gb-2012-13-6-r43)
Supplement: Additional file 1 — Supplementary figures and tables. This file contains Supplementary Figures 1 to 10 and Supplementary Tables 1 to 17. [file gb-2012-13-6-r43-S1.PDF]

Functional annotation of the human brain methylome identifies tissue-specific epigenetic variation across brain and blood

Matthew Davies[1,2], Manuela Volta[1], Ruth Pidsley[1], Katie Lunnon[1], Abhishek Dixit[1], Simon Lovestone[1], Cristian Coarfa[3], R. Alan Harris[3], Aleksandar Milosavljevic[3], Claire Troakes[1], Safa Al-Sarraj[1], Richard Dobson[1], Leonard C. Schalkwyk[1], Jonathan Mill[1]*

[1] Institute of Psychiatry, King's College London, De Crespigny Park, London, SE5 8AF, UK.

[2] Department of Twin Research and Genetic Epidemiology, King's College London, Westminster Bridge Road, London, SE1 7EH, UK.

[3] Department of Molecular and Human Genetics, Baylor College of Medicine, One Baylor Plaza, Houston, TX 77030, USA.

* Corresponding Author: Dr Jonathan Mill

Address: Institute of Psychiatry, SGDP Centre, De Crespigny Park, Denmark Hill, London. SE5 8AF. UK. Telephone: +44(0)2078480859

E-mail: jonathan.mill@kcl.ac.uk

SUPPLEMENTARY DATA

10 Supplementary Figures
17 Supplementary Tables

Additional data is available at http://epigenetics.iop.kcl.ac.uk/brain

Raw MeDIP-seq data has been submitted to the NIH Human Epigenome Atlas (www.epigenomeatlas.org) for download and browsing.

Supplementary Figure 1 - High-quality 50bp paired-end MeDIP-seq data was obtained for all samples. Shown for one representative sample are FastQC metrics of A) per base sequence quality (where the central red line is the median value, the yellow box represents the inter-quartile range (25-75%), and the upper and lower whiskers represent the 10% and 90% points while the blue line represents the mean quality), B) average sequence quality (demonstrating that the overwhelming majority of sequences have high quality values), and C) read duplication (demonstrating a low frequency of clonally-duplicated reads). D) shows the average gap insert-size distribution of mapped reads following alignment, highlighting a tight distribution around 150bp.

A

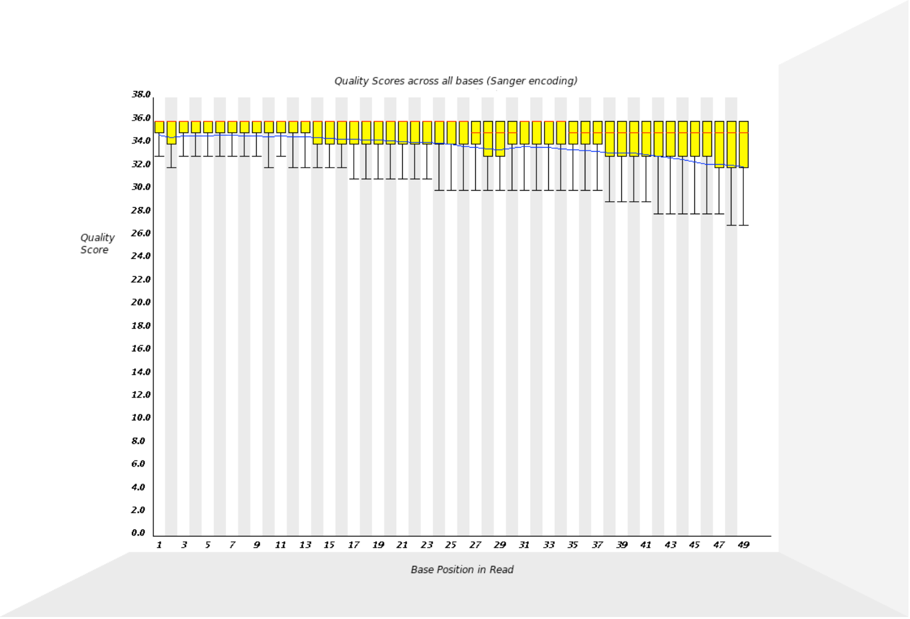

B

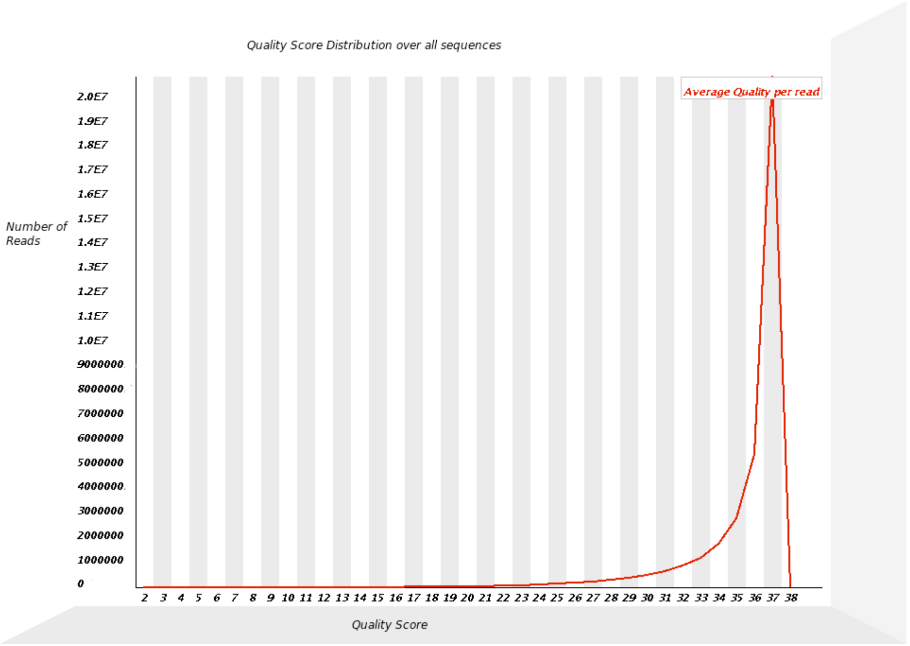

C

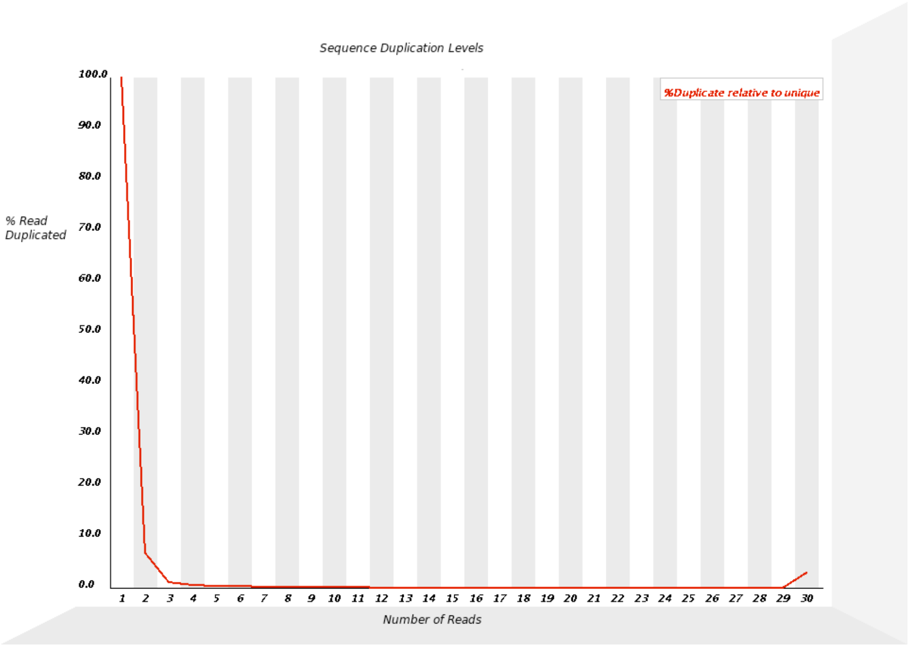
Sequence Duplication Levels
%Duplicate relative to unique
% Read Duplicated
100.0
90.0
80.0
70.0
60.0
50.0
40.0
30.0
20.0
10.0
0.0
1 2 3 4 5 6 7 8 9 10 11 12 13 14 15 16 17 18 19 20 21 22 23 24 25 26 27 28 29 30
Number of Reads

D

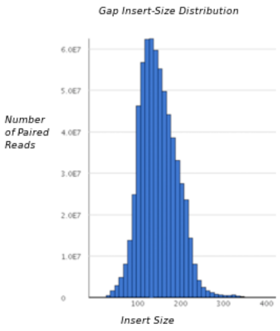
Gap Insert-Size Distribution
Number of Paired Reads
Insert Size

Supplementary Figure 2: Gene-level analysis of canonical DNA methylation patterns. A) There is a canonical decrease in DNA methylation at the transcription start site with B) a more subtle decrease also seen at the 3' end, confirming previous observations (Maunakea et al, 2010), and C) overall high levels of DNA methylation across the gene-body.

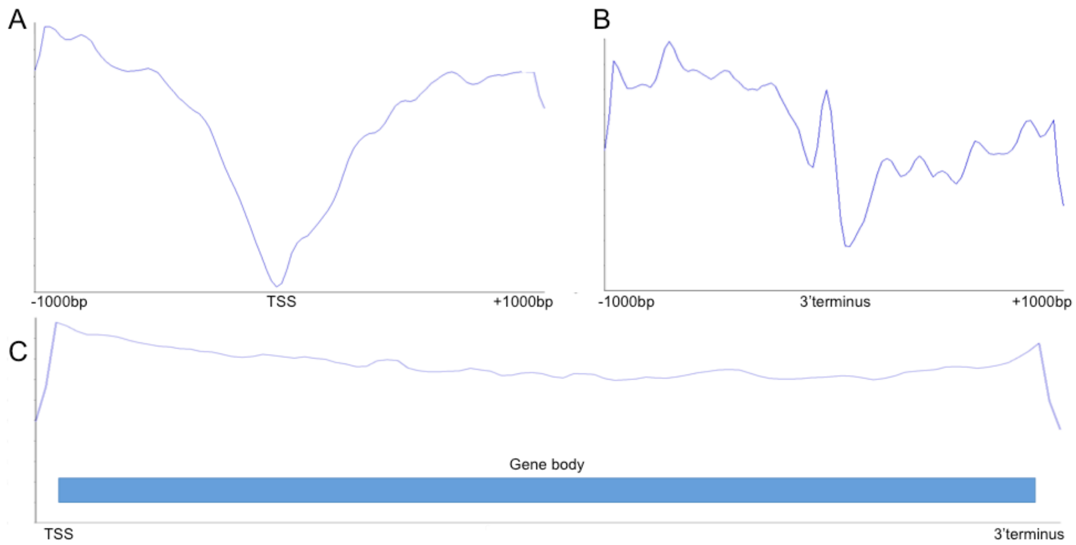

Maunakea AK, Nagarajan RP, Bilenky M, Ballinger TJ, D'Souza C, Fouse SD, Johnson BE, Hong C, Nielsen C, Zhao Y, Turecki G, Delaney A, Varhol R, Thiessen N, Shchors K, Heine VM, Rowitch DH, Xing X, Fiore C, Schillebeeckx M, Jones SJ, Haussler D, Marra MA, Hirst M, Wang T, Costello JF. Conserved role of intragenic DNA methylation in regulating alternative promoters. Nature. 2010 Jul 8;466(7303):253-7.

Supplementary Figure 3: Hierarchical clustering of MeDIP-seq data can clearly differentiate between cortex, cerebellum and blood for each broad category of annotated feature. A) CGIs, B) CGI shores, C) CDS.

A

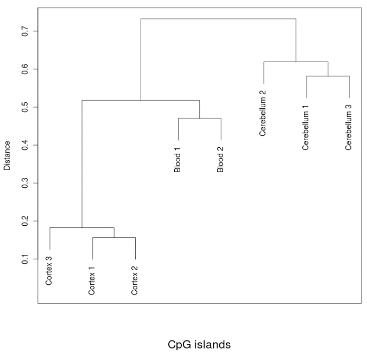

B

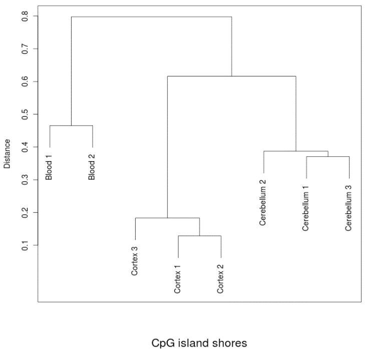

C

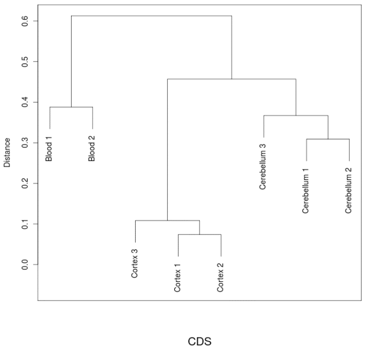

Supplementary Figure 4: Ingenuity Pathway Analysis (IPA) of the top-ranked cross-tissue variably-methylated features between cortex, cerebellum and blood highlights a highly-significant enrichment for developmental and neurobiological functions.

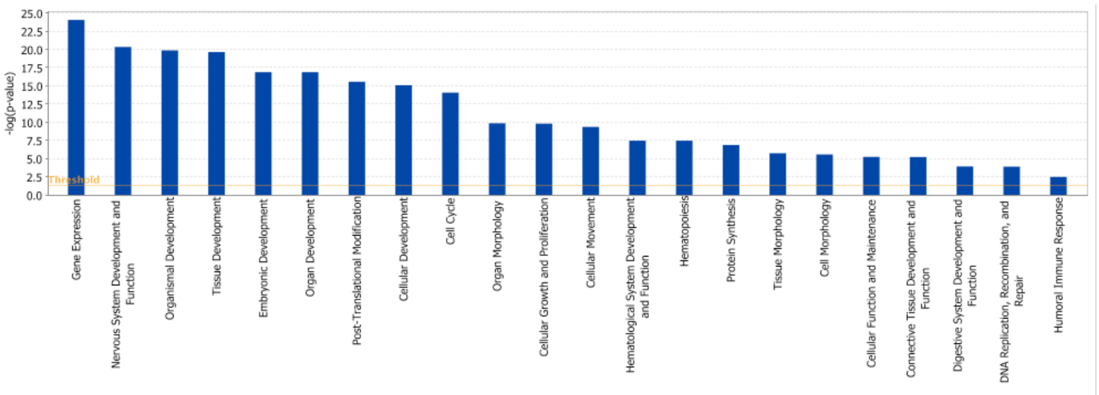

Supplementary Figure 5: IPA of the top within-brain variable DMRs (differentiating cortex from cerebellum) highlights a primary network involved in nervous system development and function.

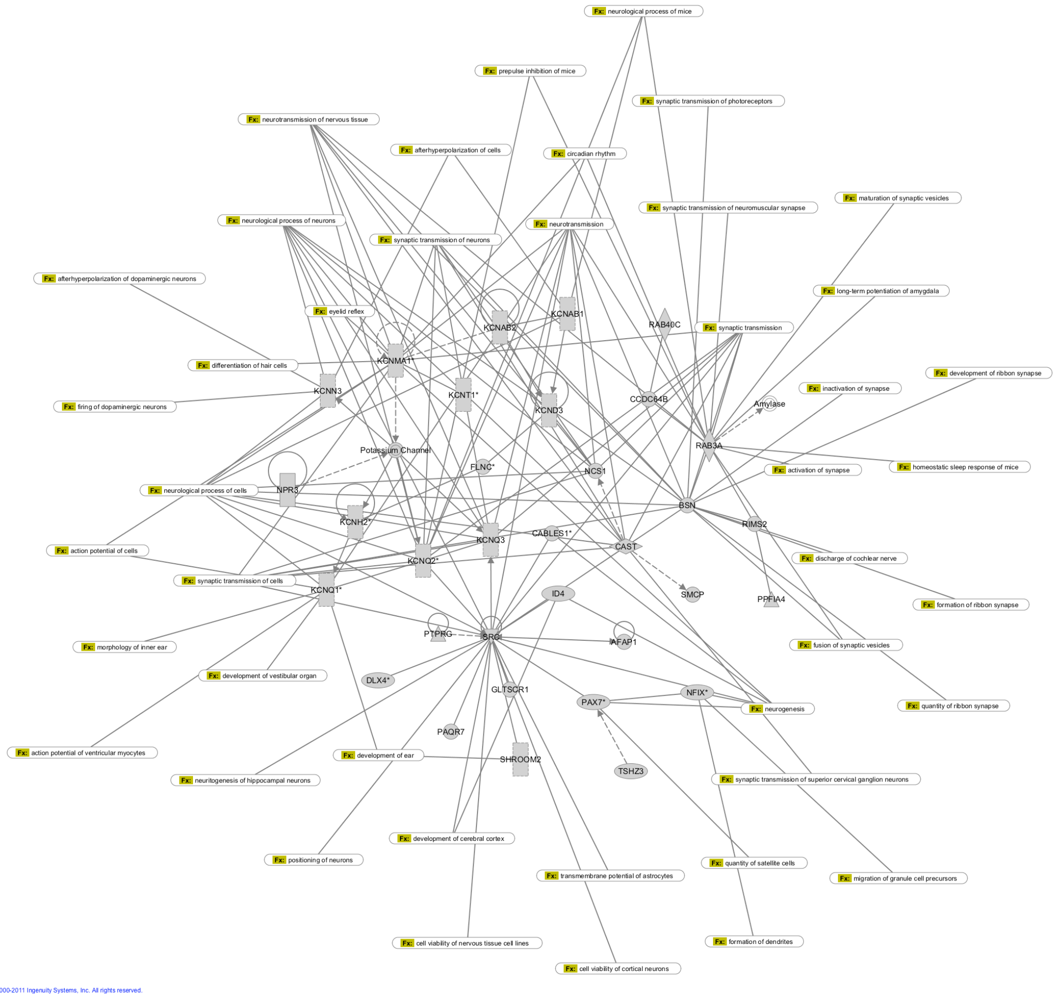

Supplementary Figure 6 – An analysis of publically available gene expression data (from Johnson et al (2009)) demonstrates that our top cortex-cerebellum DMRs are widely mirrored by stable gene expression differences between the brain regions (red circles = cerebellum; blue = cortex). Shown are two examples of genes associated with differential DNA methylation between cortex and cerebellum (*EOMES* and *JAKMIP1*).

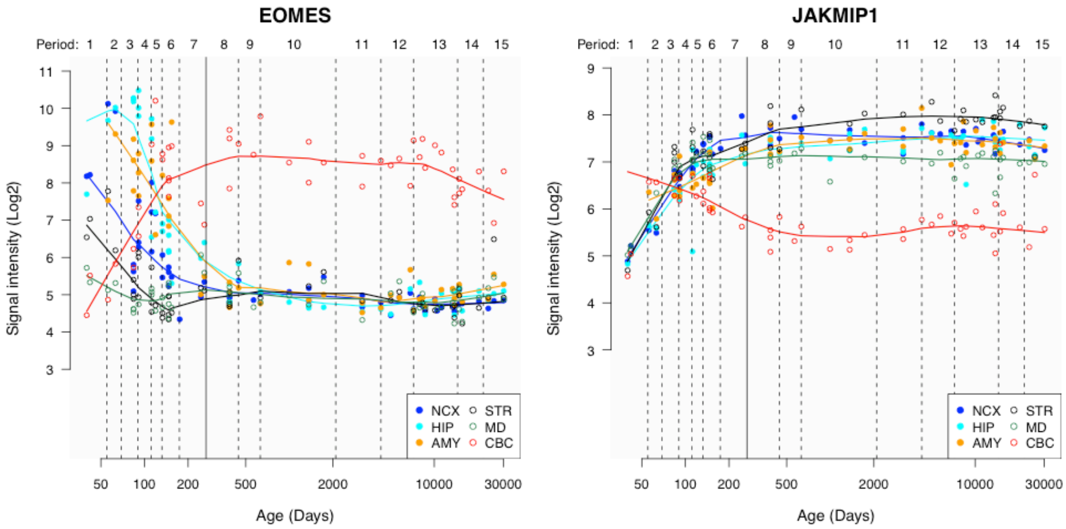

Johnson MB, Kawasawa YI, Mason CE, Krsnik Z, Coppola G, Bogdanović D, Geschwind DH, Mane SM, State MW, Sestan N. Functional and evolutionary insights into human brain development through global transcriptome analysis. Neuron. 2009 May 28;62(4):494-509.

Supplementary Figure 7 - The top-ranked network incorporating across-cortex DMRs is significantly enriched for biological functions associated with neurogenesis and cortical function.

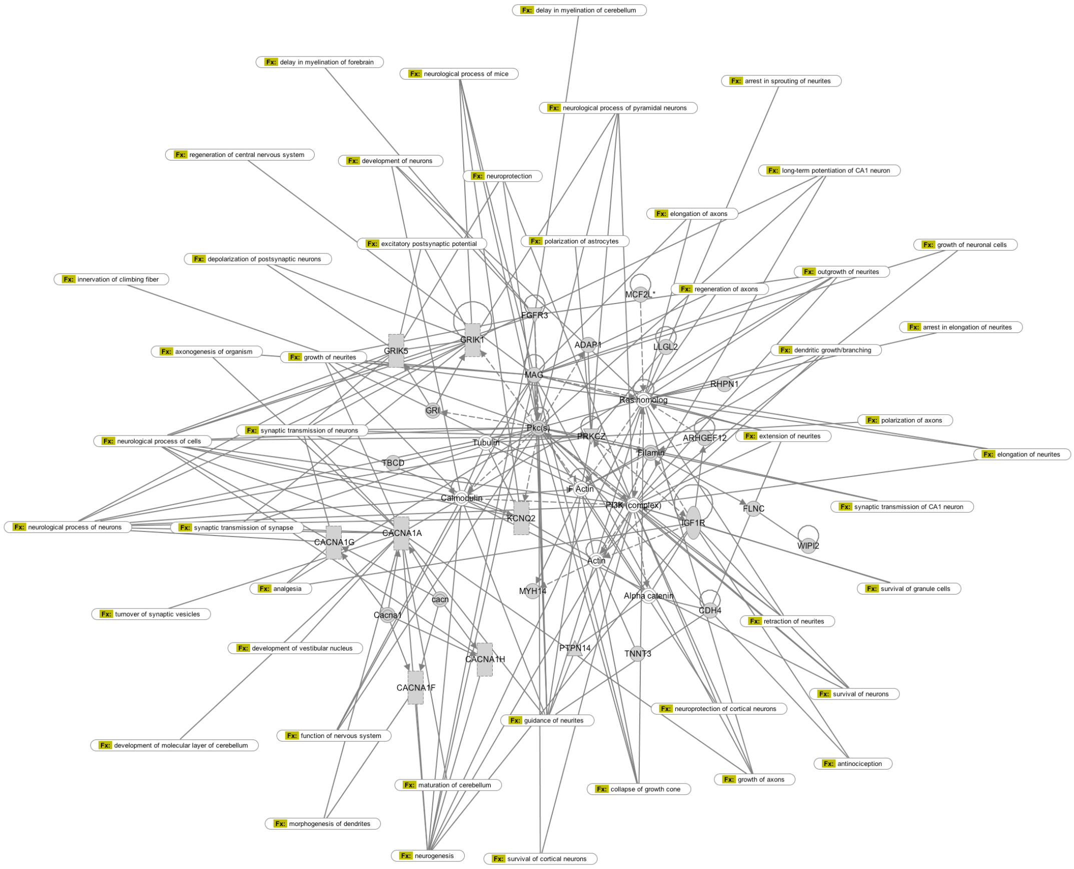

Supplementary Figure 8 – DNA methylation values estimated using bisulfite pyrosequencing is significantly correlated with MEDIPS scores derived from MeDIP-seq data. Shown is the correlation between MEDIPS scores (derived from MeDIP-seq data) and bisulfite pyrosequencing data (using an average of DNA methylation across all CpG sites interrogated in each amplicon) for the DMRs selected for verification analysis (corr = 0.58, p = 7.07 X 10^{-13}).

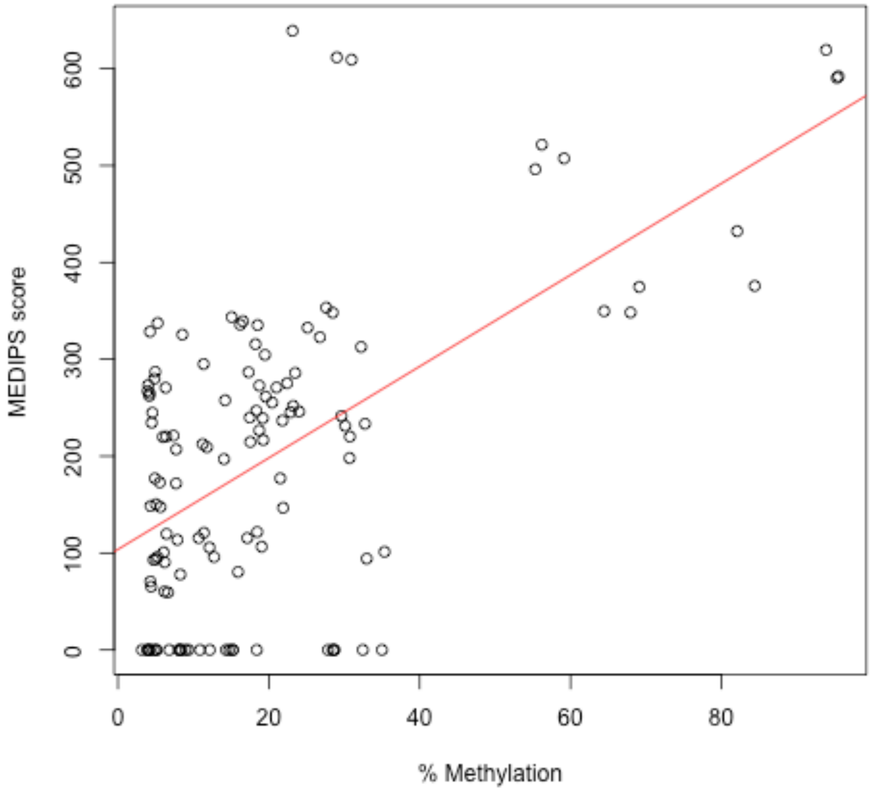

Supplementary Figure 9 - Unlike for CGIs there is no significant over-representation of intragenic location for the most tissue-variable differentially methylated CGI shores.

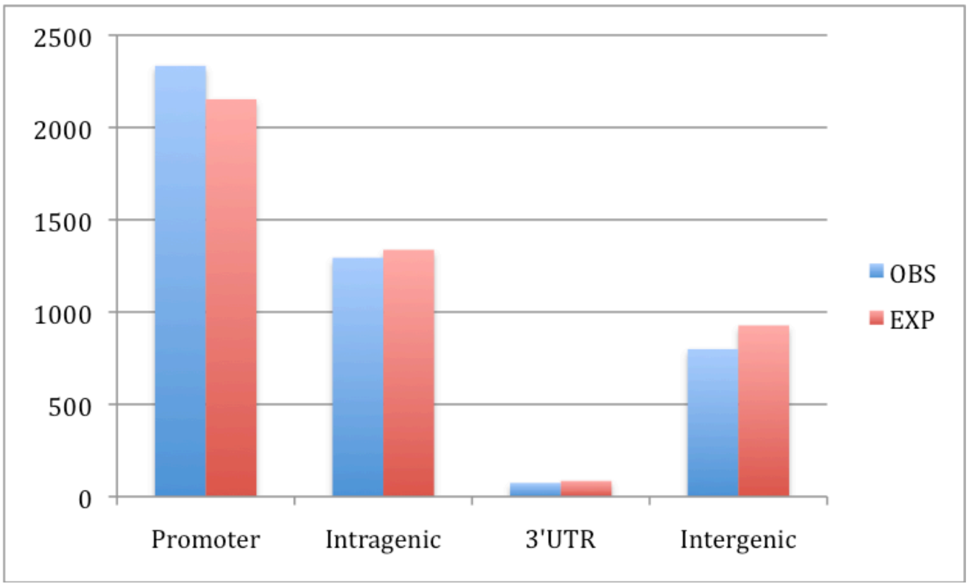

Supplementary Figure 10 - Tissue-specific DNA methylation across shores flanking an intergenic CGI on chromosome 7. A) MeDIP-seq data shows this region is hypermethylated in cerebellum DNA compared to cortex and blood from the same individuals, with higher overall DNA methylation in the shores compared to the CGI. B) The MeDIP-seq data was verified using bisulfite-Pyrosequencing on an extended set of samples with significantly elevated cerebellar methylation across both shores. Again, our pyrosequencing data showed a highly significant correlation with MEDIPS scores across each of the three amplicons (left shore: corr = 0.76, p = 5.58 X 10^{-5}; CGI: corr = 0.80, p = 1.18 X 10^{-5}; right shore: corr = 0.82, p = 4.36 X 10^{-6}) confirming the validity of the methylome data.

A

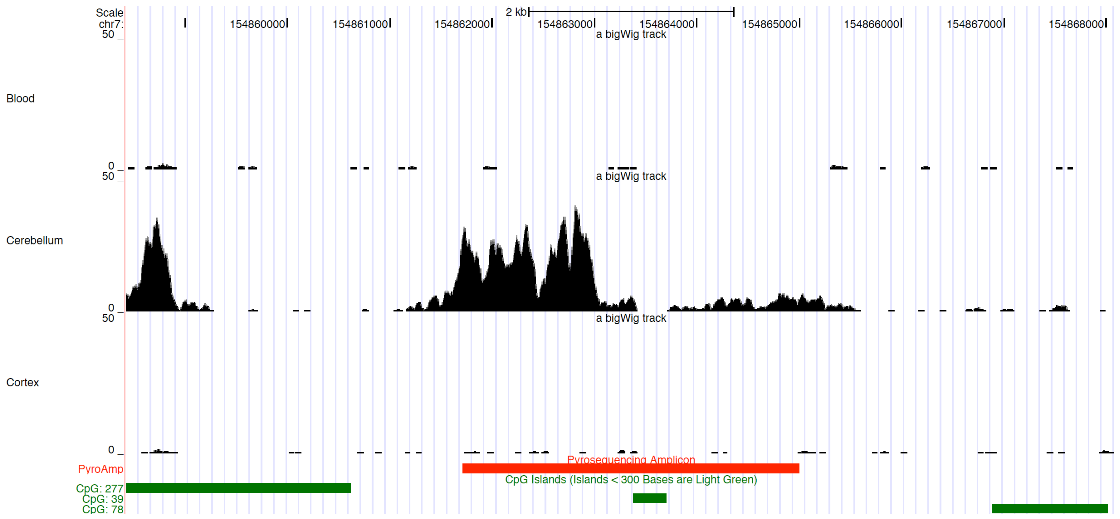

B

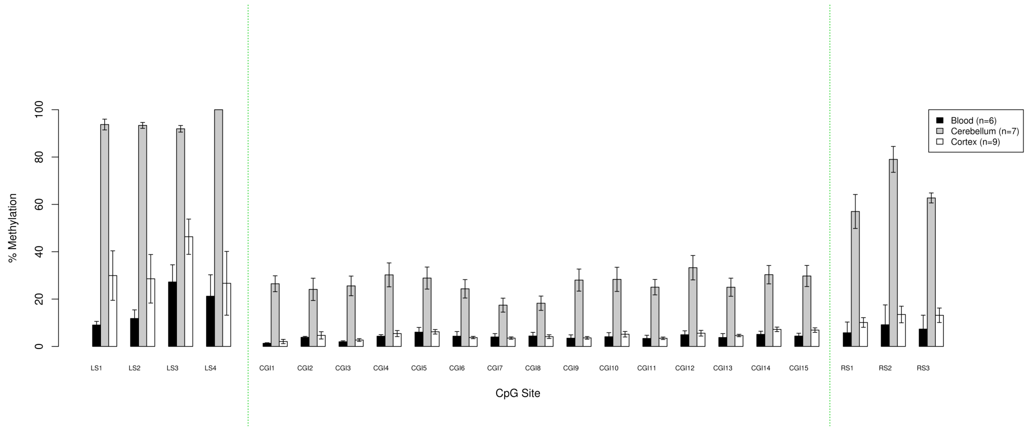

Supplementary Table 1 – Demographic information about the primary samples used in this study

Individual	Sex	Age at Time of Death	Postmortem Delay (hrs)	Tissue Type	Region	Full Tissue Description	MeDIP-seq	Pyrosequencing
1	Female	78	43	Cortex	BA9	Inferior frontal gyrus	x	x
					BA10	Inferior frontal gyrus	x	x
					BA8	Middle frontal gyrus	x	x
					Ent Cortex	Entorhinal Cortex	x	x
					STG BA21	Superior temporal gyrus of the temporal cortex	x	x
					BA 17	Visual Cortex		x
				Cerebellum	Cerebellum	Cerebellum	x	x
				Blood	Blood	Whole Blood	x	x
2	Female	92	17	Cortex	BA9	Inferior frontal gyrus	x	x
					BA10	Inferior frontal gyrus	x	x
					BA46	Dorsolateral prefrontal cortex		x
					BA8	Middle frontal gyrus	x	x
					Ent Cortex	Entorhinal Cortex	x	x
					STG BA21	Superior temporal gyrus of the temporal cortex	x	x
					BA 17	Visual Cortex	x	x
				Cerebellum	Cerebellum	Cerebellum	x	x
				Blood	Blood	Whole Blood - time 1	x	x
					Blood	Whole Blood - time 2		x
					Blood	Whole Blood - time 3		x
3	Male	80	10	Cortex	BA9	Inferior frontal gyrus	x	x
					BA10	Inferior frontal gyrus	x	x
					BA46	Dorsolateral prefrontal cortex		x
					BA8	Middle frontal gyrus	x	x
					Ent Cortex	Entorhinal Cortex	x	x
					STG BA21	Superior temporal gyrus of the temporal cortex	x	x

				Cerebellum	Cerebellum	Cerebellum	x	x
				Blood	Blood	Whole Blood		x
4	Male	80	60	Cortex	BA9	Inferior frontal gyrus		x
					BA10	Inferior frontal gyrus		x
					BA46	Dorsolateral prefrontal cortex		x
					BA8	Middle frontal gyrus		x
					Ent Cortex	Entorhinal Cortex		x
					STG BA21	Superior temporal gyrus of the temporal cortex		x
					BA 17	Visual Cortex		x
				Blood	Blood	Whole Blood		x
5	Male	86	7	Cortex	BA9	Inferior frontal gyrus		x
					Ent Cortex	Entorhinal Cortex		x
				Cerebellum	Cerebellum	Cerebellum		x
6	Female	90	50	Cortex	BA9	Inferior frontal gyrus		x
					STG BA21	Superior temporal gyrus of the temporal cortex		x
				Cerebellum	Cerebellum	Cerebellum		x
7	Female	92	53	Cortex	BA9	Inferior frontal gyrus		x
					BA8	Middle frontal gyrus		x
					Ent Cortex	Entorhinal Cortex		x
					STG BA21	Superior temporal gyrus of the temporal cortex		x
					BA 17	Visual Cortex		x
				Cerebellum	Cerebellum	Cerebellum		x
				Blood	Blood	Whole Blood - time 1		x
					Blood	Whole Blood - time 2		x
					Blood	Whole Blood - time 3		x
8	Male	94	41	Cortex	BA9	Inferior frontal gyrus		x
					BA10	Inferior frontal gyrus		x
					BA8	Middle frontal gyrus		x
					Ent Cortex	Entorhinal Cortex		x

					STG BA21	Superior temporal gyrus of the temporal cortex		x
				Cerebellum	Cerebellum	Cerebellum		x
				Blood	Blood	Whole Blood		x
9	Female	88	22	Cortex	BA9	Inferior frontal gyrus		x
					BA10	Inferior frontal gyrus		x
					BA8	Middle frontal gyrus		x
				Cerebellum	Cerebellum	Cerebellum		x

Supplementary Table 2 – The number of high-quality mapped 50bp sequencing reads produced from each sample interrogated by MeDIP-seq.

Sample	Tissue	Aligned sequences	Unaligned/Duplicates
1	*BA8*	70293901	3175487
	BA10	70258373	3211015
	BA9	70061288	5029260
	Entorhinal Cortex	67170741	6298647
	STG	70398381	3071007
	Cerebellum	69383858	4085530
	Blood	63155427	10313961
2	*BA8*	69936969	3532419
	BA10	69975804	3493584
	BA9	70224376	3245012
	Entorhinal Cortex	68188747	5280641
	STG	70461293	3008095
	Visual Cortex	69569856	3899532
	Cerebellum	68128585	5340803
	Blood	61823416	11645972
3	*BA8*	70271989	3197399
	BA10	76948447	8765837
	BA9	76990455	8723829
	Entorhinal Cortex	75335441	7199885
	STG	78632240	7082050
	Cerebellum	71587111	1882277
**Average**		70418890	5308678

Supplementary Table 3 – The top 50 annotated DMR features across blood, cerebellum and cortex. Values correspond to normalized MeDIP-seq read-counts across each feature location. Data for all annotated features is available for download from http://epigenetics.iop.kcl.ac.uk/brain.

Location	Feature Type	Gene	Cerebellum	Cortical	Blood	Mean	CV
7:154861384-154863384	Shore	AC008060.7	189.46	1.02	1.12	63.87	1.68
7:154860615-154862615	Shore	AC008060.7	136.11	0.98	1.15	46.08	1.66
14:56351720-56353720	Shore		98.30	1.30	0.37	33.32	1.64
13:76358137-76359416	HCP	KCTD12	0.00	0.68	64.92	21.87	1.63
19:4987959-4988220	Intragenic CGI	JMJD2B	1.39	6.94	241.20	83.18	1.63
12:52375478-52375680	Intergenic CGI		78.23	1.42	0.00	26.55	1.62
1:152014113-152014202	CDS	AL513523.33	0.00	0.71	59.15	19.95	1.62
9:2231337-2231659	Intergenic CGI		0.00	4.57	140.17	48.25	1.62
5:58368593-58370593	Shore	PDE4D	163.28	4.91	1.14	56.44	1.61
5:146235539-146237539	Shore	PPP2R2B	91.93	1.07	1.51	31.50	1.61
9:21445722-21445937	CDS	AL353732.14	0.00	38.17	0.00	12.72	1.61
8:67617099-67617545	Intergenic CGI		0.81	8.99	210.51	73.44	1.60
1:67924858-67926858	Shore	GADD45A	160.01	3.98	3.35	55.78	1.59
11:82211137-82211244	CDS	AP001646.5	669.95	15.78	21.36	235.69	1.59
7:10979479-10980468	HCP	PHF14	0.37	2.44	79.22	27.34	1.59
14:105523714-105524151	CDS	IGHV1-2	94.30	3.88	0.00	32.72	1.58
14:56348869-56350869	Shore		133.97	2.97	3.41	46.79	1.58
5:58371638-58373638	Shore	PDE4D	114.21	2.96	2.25	39.81	1.58
8:125808866-125810447	Promoter CGI	MTSS1	0.77	1.12	59.95	20.61	1.58
2:56266043-56268043	Shore	CCDC85A	259.55	9.40	5.69	91.55	1.57
14:56348462-56348869	Intergenic CGI		44.35	1.03	0.00	15.13	1.57
7:77813545-77814824	LCP	MAGI2	86.37	2.06	1.78	30.07	1.57
4:40447448-40447653	Intragenic CGI	NSUN7	1.76	2.31	88.00	30.69	1.57
1:20384275-20385544	ICP	UBXD3	226.70	10.66	2.98	80.11	1.57
12:123962801-123962902	CDS	UBA52	4.06	5.43	163.23	57.58	1.56
17:44054082-44056082	Shore	HOXB9	2.16	3.58	109.04	38.26	1.56

16:46952992-46953840	CDS	SIAH	0.98	2.86	79.90	27.91	1.56
3:160001747-160002976	HCP	MFSD1	0.59	1.50	55.15	19.08	1.56
8:143855281-143856413	Intergenic CGI	LYNX1	0.00	1.41	45.34	15.58	1.55
17:73389603-73390067	CDS	AC015804.14	704.07	44.11	6.49	251.56	1.55
16:46952992-46953888	CDS	SIAH	0.92	2.78	75.63	26.44	1.55
7:27121524-27121951	Intragenic CGI	AC010990.3	0.00	3.88	77.31	27.06	1.55
8:26428747-26430747	Shore		81.94	2.27	1.91	28.71	1.55
6:33156394-33156792	Intragenic CGI	HLA-DPB1	1.04	5.36	110.80	39.07	1.55
14:56348040-56350040	Shore		112.43	2.75	3.78	39.66	1.55
1:82041636-82043636	Shore		90.19	3.41	1.51	31.70	1.55
1:120707494-120707919	Intergenic CGI	AL357493.8	2.56	3.99	109.89	38.81	1.55
2:171538156-171538954	Intergenic CGI		0.00	1.17	39.64	13.60	1.54
15:88256459-88256899	CDS	C15orf38	0.00	0.16	25.62	8.59	1.54
5:59225651-59227651	Shore	PDE4D	105.72	4.83	1.91	37.49	1.54
3:50332457-50332687	Intragenic CGI	HYAL2	0.00	13.76	188.57	67.44	1.54
1:146248690-146249097	Intergenic CGI		3.96	3.29	111.98	39.74	1.54
4:53422361-53423640	HCP	RASL11B	0.57	0.66	37.84	13.02	1.53
7:140761670-140762610	CDS	AC005692.1	103.32	1.96	4.75	36.68	1.53
17:44055279-44057279	Shore	HOXB9	1.96	2.39	75.05	26.47	1.53
7:27149413-27150692	HCP	HOXA5	2.82	5.09	116.53	41.48	1.53
7:27130344-27130623	Intragenic CGI	AC010990.3	3.03	7.42	145.43	51.96	1.53
17:19712201-19712406	Promoter CGI	ULK2	3.78	6.74	146.14	52.22	1.53
13:66703460-66705460	Shore	PCDH9	111.66	3.76	3.81	39.74	1.53
14:61348790-61349092	Intergenic CGI		1.37	2.45	67.15	23.66	1.53

Supplementary Table 4 – Confirmation of the top-ranked cross-tissue DMR features identified by MeDIP-seq using the Illumina HumanMethylation 450 array in an independent sample of 90 matched cerebellum, frontal cortex and blood samples. Shown are tissue-averaged β-values ranging from 0 (unmethylated) to 1 (fully methylated) for cerebellum (CB), frontal cortex (F. CTX) and blood (BLD) at CpG sites interrogated by the array (n=206) within the broad regions spanned by the annotated features in Supplementary Table 3. P-values were generated using a mixed model test for tissue differences.

MeDIP-seq DMR rank	Hypermethylated tissue (MeDIP-seq)	Illumina 450K Probe	CB	F. CTX	BLD	Standard Deviation	p-value	CB Av	F. CTX Av	BLD Av
1	CB	cg15731180	0.76	0.30	0.22	0.29	5.89E-120	0.76	0.30	0.22
2	CB	cg10823693	0.85	0.26	0.13	0.38	6.39E-153	0.77	0.17	0.10
		cg25859141	0.70	0.07	0.06	0.36	1.43E-125			
3	CB	cg14409023	0.12	0.09	0.11	0.02	2.58E-25	0.30	0.16	0.15
		cg22967396	0.71	0.23	0.22	0.28	8.32E-127			
		cg24191463	0.22	0.18	0.16	0.03	1.83E-16			
		cg26972272	0.15	0.15	0.13	0.01	ns			
4	BLD	cg00931644	0.14	0.27	0.67	0.28	2.35E-95	0.07	0.09	0.16
		cg02481778	0.03	0.02	0.03	0.00	ns			
		cg02722456	0.04	0.04	0.04	0.00	ns			
		cg04486282	0.03	0.03	0.03	0.00	ns			
		cg09243759	0.02	0.02	0.03	0.00	ns			
		cg11004501	0.02	0.02	0.03	0.00	ns			
		cg12280407	0.11	0.09	0.10	0.01	ns			
		cg16650292	0.03	0.03	0.03	0.00	ns			
		cg19938422	0.02	0.02	0.02	0.00	ns			
		cg20248204	0.12	0.23	0.37	0.12	6.25E-80			
		cg24515970	0.04	0.04	0.04	0.00	ns			
		cg25889945	0.10	0.08	0.09	0.01	ns			
		cg25968569	0.15	0.27	0.60	0.23	3.00E-90			
		cg27275523	0.11	0.12	0.11	0.00	ns			
5	BLD	cg07505433	0.18	0.24	0.81	0.35	5.26E-176	0.17	0.31	0.83

		cg10124355	0.14	0.30	0.79	0.34	2.49E-175			
		cg14694038	0.15	0.27	0.87	0.39	1.59E-181			
		cg17767427	0.21	0.41	0.85	0.33	2.42E-160			
6	CB	cg02675414	0.45	0.17	0.12	0.18	6.66E-116	0.53	0.15	0.11
		cg16917193	0.62	0.14	0.11	0.28	8.94E-80			
7	BLD	cg21279955	0.11	0.26	0.33	0.11	6.07E-88	0.11	0.26	0.33
9	CB	cg07200957	0.35	0.23	0.08	0.13	3.44E-84	0.35	0.23	0.08
10	CB	cg19335130	0.14	0.30	0.39	0.12	ns	0.13	0.22	0.26
		cg22642777	0.12	0.15	0.13	0.02	ns			
12	BLD	cg08620329	0.13	0.18	0.69	0.31	2.33E-95	0.11	0.21	0.61
		cg10687107	0.09	0.25	0.61	0.27	2.21E-107			
		cg12109823	0.05	0.11	0.68	0.35	3.78E-102			
		cg15444185	0.14	0.20	0.46	0.17	1.66E-88			
		cg20902757	0.14	0.31	0.63	0.25	4.91E-105			
13	CB	cg20783262	0.16	0.13	0.19	0.03	ns	0.16	0.13	0.19
15	BLD	cg06715026	0.09	0.08	0.08	0.01	ns	0.13	0.12	0.12
		cg07287563	0.14	0.12	0.11	0.01	ns			
		cg07378472	0.11	0.09	0.11	0.01	ns			
		cg07574654	0.13	0.11	0.11	0.02	ns			
		cg10772238	0.07	0.08	0.09	0.01	1.93E-09			
		cg11931558	0.08	0.07	0.08	0.00	ns			
		cg13029706	0.04	0.03	0.03	0.00	ns			
		cg16800461	0.13	0.11	0.10	0.02	ns			
		cg20964328	0.04	0.03	0.04	0.00	ns			
		cg22477374	0.05	0.05	0.05	0.00	ns			
		cg22876561	0.05	0.05	0.05	0.00	ns			
		cg23083824	0.11	0.10	0.09	0.01	ns			
		cg24491907	0.11	0.09	0.09	0.01	ns			
		cg27578754	0.65	0.63	0.70	0.03	3.26E-22			
17	CB	cg02161478	0.73	0.32	0.13	0.31	6.55E-90	0.73	0.37	0.19

		cg12152002	0.76	0.50	0.24	0.26	4.66E-85			
		cg23478293	0.71	0.30	0.18	0.28	2.21E-79			
18	CB	cg09092093	0.24	0.17	0.10	0.07	4.78E-51	0.24	0.17	0.10
19	BLD	cg01013890	0.11	0.09	0.11	0.01	ns	0.25	0.20	0.31
		cg04120546	0.11	0.08	0.17	0.05	1.27E-48			
		cg06710295	0.12	0.09	0.11	0.01	ns			
		cg10408778	0.06	0.05	0.06	0.01	3.44E-15			
		cg12047218	0.04	0.03	0.04	0.00	ns			
		cg16500409	0.11	0.09	0.10	0.01	ns			
		cg18280057	0.21	0.36	0.81	0.31	2.85E-139			
		cg18893120	0.32	0.34	0.80	0.27	2.42E-119			
		cg19523819	0.33	0.33	0.74	0.23	6.39E-136			
		cg26069068	0.33	0.30	0.77	0.26	5.28E-136			
		cg27136289	0.13	0.11	0.11	0.01	ns			
20	CB	cg06716959	0.36	0.06	0.04	0.18	4.92E-111	0.56	0.24	0.17
		cg10664190	0.78	0.47	0.39	0.20	1.59E-69			
		cg14552376	0.10	0.08	0.09	0.01	ns			
		cg17501259	0.62	0.18	0.07	0.29	6.05E-127			
		cg19516982	0.85	0.53	0.31	0.27	2.36E-103			
		cg24130711	0.66	0.13	0.10	0.32	3.71E-134			
21	CB	cg09624466	0.75	0.17	0.22	0.32	1.90E-99	0.78	0.22	0.19
		cg21039708	0.80	0.13	0.15	0.38	2.10E-108			
		cg25092838	0.77	0.35	0.19	0.30	8.05E-83			
22	CB	cg09196534	0.87	0.83	0.73	0.07	5.97E-79	0.84	0.83	0.79
		cg12671090	0.81	0.84	0.84	0.02	ns			
23	BLD	cg12812233	0.15	0.20	0.46	0.17	1.78E-87	0.15	0.17	0.37
		cg24296765	0.18	0.22	0.49	0.17	1.41E-104			
		cg24621790	0.11	0.10	0.15	0.03	1.80E-28			
24	CB	cg03167763	0.50	0.30	0.14	0.18	1.43E-92	0.62	0.37	0.17
		cg06090867	0.76	0.76	0.33	0.25	1.22E-100			

		cg06911113	0.62	0.36	0.13	0.25	7.53E-125			
		cg08130175	0.89	0.77	0.44	0.23	1.14E-114			
		cg11651220	0.67	0.16	0.05	0.33	9.54E-121			
		cg19696441	0.63	0.22	0.07	0.29	1.56E-109			
		cg24479524	0.61	0.28	0.11	0.26	1.35E-110			
		cg24685601	0.24	0.10	0.06	0.10	8.55E-66			
26	BLD	cg07048527	0.21	0.28	0.87	0.36	1.91E-159	0.18	0.25	0.77
		cg12057127	0.15	0.22	0.68	0.29	9.71E-123			
27	BLD	cg02167757	0.85	0.81	0.78	0.03	1.85E-51	0.85	0.81	0.78
28	BLD	cg06667063	0.03	0.03	0.04	0.01	ns	0.08	0.07	0.11
		cg07765813	0.04	0.04	0.05	0.01	ns			
		cg09459188	0.09	0.07	0.08	0.01	ns			
		cg11632438	0.30	0.18	0.46	0.14	9.43E-90			
		cg12745387	0.11	0.08	0.09	0.01	ns			
		cg16289538	0.04	0.04	0.04	0.00	ns			
		cg19403653	0.05	0.05	0.05	0.00	ns			
		cg20216950	0.03	0.03	0.03	0.00	ns			
		cg23208397	0.10	0.07	0.08	0.01	ns			
		cg25050026	0.11	0.09	0.25	0.09	9.75E-75			
		cg26149032	0.04	0.05	0.07	0.02	4.06E-49			
29	BLD	cg03405983	0.16	0.22	0.42	0.13	1.17E-96	0.10	0.10	0.15
		cg04688051	0.04	0.05	0.09	0.03	5.04E-28			
		cg04688828	0.04	0.04	0.06	0.01	3.10E-13			
		cg04969067	0.05	0.05	0.05	0.00	ns			
		cg05072100	0.13	0.11	0.21	0.05	2.22E-44			
		cg05569086	0.15	0.14	0.18	0.02	2.60E-23			
		cg10404336	0.16	0.14	0.15	0.01	ns			
		cg12516270	0.07	0.07	0.07	0.00	ns			
		cg15511327	0.09	0.09	0.14	0.03	4.52E-57			
		cg17888033	0.15	0.17	0.46	0.18	3.82E-98			

		cg20041105	0.11	0.10	0.12	0.01	3.72E-14			
		cg20541178	0.18	0.14	0.18	0.02	6.22E-18			
		cg22687807	0.03	0.03	0.03	0.00	ns			
		cg24046110	0.12	0.09	0.10	0.01	ns			
		cg26559315	0.04	0.04	0.05	0.01	ns			
32	BLD	cg04351734	0.25	0.28	0.78	0.30	8.42E-157	0.13	0.23	0.89
		cg16406967	0.07	0.23	0.95	0.47	3.76E-165			
		cg16748008	0.08	0.15	0.91	0.46	1.65E-210			
		cg18680977	0.07	0.27	0.97	0.47	2.82E-151			
		cg19999161	0.12	0.23	0.89	0.42	2.74E-166			
		cg22798849	0.16	0.23	0.87	0.39	5.04E-169			
33	CB	cg26268277	0.47	0.19	0.23	0.15	9.09E-98	0.47	0.19	0.23
34	BLD	cg00570906	0.17	0.41	0.71	0.27	4.51E-108	0.11	0.25	0.65
		cg01132696	0.05	0.11	0.55	0.27	1.30E-93			
		cg02692313	0.09	0.18	0.68	0.32	9.07E-75			
		cg03229061	0.15	0.26	0.59	0.23	1.66E-99			
		cg03636880	0.14	0.33	0.69	0.27	6.01E-103			
		cg06437840	0.05	0.09	0.55	0.28	4.37E-79			
		cg10850215	0.06	0.18	0.59	0.28	1.40E-99			
		cg12893780	0.09	0.25	0.64	0.29	2.01E-100			
		cg13581859	0.16	0.35	0.67	0.26	6.73E-104			
		cg14801692	0.21	0.30	0.61	0.21	5.22E-97			
		cg14870156	0.11	0.18	0.71	0.33	2.19E-105			
		cg15829535	0.14	0.26	0.62	0.25	1.01E-100			
		cg17588455	0.15	0.24	0.57	0.22	8.67E-93			
		cg19990651	0.05	0.12	0.62	0.31	4.89E-98			
		cg20223237	0.07	0.38	0.77	0.35	9.01E-107			
		cg25511667	0.09	0.27	0.71	0.32	2.07E-99			
		cg26645432	0.16	0.29	0.79	0.33	7.15E-112			
35	CB	cg05241355	0.71	0.14	0.16	0.32	4.61E-95	0.71	0.14	0.16

36	CB	cg13559233	0.12	0.13	0.16	0.02	ns	0.22	0.16	0.15
		cg13980528	0.16	0.13	0.12	0.02	8.11E-22			
		cg14529474	0.38	0.22	0.17	0.11	2.30E-78			
37	BLD	cg05659741	0.13	0.20	0.61	0.26	3.81E-118	0.13	0.20	0.61
38	BLD	cg00677479	0.15	0.22	0.28	0.07	7.08E-60	0.11	0.13	0.14
		cg11422521	0.05	0.05	0.04	0.01	ns			
		cg23632706	0.13	0.10	0.11	0.02	ns			
39	BLD	cg09234378	0.04	0.04	0.06	0.01	2.48E-20	0.05	0.05	0.11
		cg13556511	0.04	0.04	0.08	0.02	1.71E-42			
		cg14292280	0.03	0.03	0.06	0.02	4.64E-31			
		cg15096085	0.10	0.08	0.23	0.08	1.25E-68			
40	CB	cg08554295	0.04	0.04	0.05	0.01	ns	0.04	0.04	0.05
41	BLD	cg00467652	0.52	0.71	0.84	0.16	1.89E-101	0.51	0.71	0.86
		cg09412061	0.46	0.68	0.87	0.20	9.99E-129			
		cg26794477	0.54	0.74	0.86	0.16	2.04E-104			
42	BLD	cg03242265	0.12	0.19	0.55	0.23	2.49E-97	0.18	0.24	0.61
		cg13067553	0.06	0.12	0.50	0.24	1.37E-89			
		cg13392957	0.31	0.38	0.73	0.23	5.50E-111			
		cg23206461	0.24	0.27	0.68	0.25	5.78E-107			
43	BLD	cg00338735	0.11	0.09	0.13	0.02	7.94E-26	0.16	0.22	0.31
		cg00791764	0.23	0.41	0.62	0.20	4.80E-78			
		cg03786743	0.24	0.42	0.48	0.12	4.43E-63			
		cg14704921	0.03	0.06	0.08	0.02	8.21E-24			
		cg21521518	0.29	0.28	0.39	0.06	1.89E-27			
		cg23337116	0.05	0.05	0.06	0.00	2.11E-08			
		cg26260408	0.16	0.26	0.38	0.11	2.82E-76			
45	BLD	cg00846995	0.13	0.13	0.12	0.00	ns	0.13	0.13	0.12
46	BLD	cg01370449	0.15	0.22	0.66	0.28	1.09E-102	0.28	0.31	0.72
		cg02005600	0.23	0.29	0.68	0.24	3.16E-78			
		cg02248486	0.19	0.23	0.65	0.26	4.61E-87			

		cg02646423	0.33	0.32	0.57	0.14	3.14E-69			
		cg02916332	0.26	0.30	0.63	0.20	3.16E-77			
		cg03207666	0.48	0.52	0.85	0.21	6.19E-119			
		cg04863892	0.15	0.22	0.65	0.27	1.25E-84			
		cg05835726	0.25	0.26	0.72	0.27	2.01E-93			
		cg08070327	0.51	0.56	0.84	0.17	1.48E-86			
		cg09549073	0.18	0.24	0.61	0.23	7.26E-69			
		cg09880291	0.38	0.38	0.85	0.27	2.23E-126			
		cg12015737	0.20	0.24	0.81	0.34	2.15E-134			
		cg12128839	0.08	0.10	0.59	0.29	4.77E-84			
		cg13512268	0.32	0.31	0.83	0.30	5.02E-121			
		cg14014955	0.37	0.37	0.68	0.18	9.43E-69			
		cg14058329	0.31	0.40	0.86	0.29	4.72E-126			
		cg14658493	0.32	0.36	0.79	0.26	1.75E-98			
		cg16997642	0.35	0.41	0.82	0.26	6.15E-125			
		cg17569124	0.16	0.17	0.60	0.25	8.95E-75			
		cg19196335	0.45	0.47	0.86	0.23	2.20E-129			
		cg19759481	0.14	0.16	0.64	0.28	5.71E-84			
		cg20517050	0.22	0.24	0.64	0.24	1.43E-88			
		cg23204968	0.31	0.34	0.75	0.25	5.63E-103			
		cg23454797	0.37	0.37	0.82	0.26	1.96E-107			
		cg23936031	0.09	0.18	0.67	0.32	6.59E-99			
		cg24389585	0.39	0.44	0.86	0.26	2.44E-149			
		cg25307665	0.05	0.09	0.59	0.30	3.05E-82			
		cg25506432	0.58	0.59	0.75	0.10	7.62E-48			
		cg25866143	0.22	0.30	0.69	0.25	6.80E-96			
47	BLD	cg03536885	0.19	0.24	0.87	0.38	6.82E-184	0.17	0.24	0.87
		cg24360871	0.15	0.25	0.86	0.39	4.19E-165			
48	BLD	cg04036329	0.26	0.51	0.82	0.28	4.57E-128	0.30	0.57	0.82
		cg10543374	0.32	0.71	0.83	0.27	1.10E-134			

		cg17598378	0.46	0.76	0.87	0.21	3.89E-130			
		cg17665699	0.15	0.32	0.78	0.33	3.65E-143			
49	CB	cg01911068	0.08	0.07	0.11	0.02	ns	0.08	0.07	0.11
50	BLD	cg01843148	0.12	0.09	0.22	0.07	2.57E-47	0.12	0.09	0.22

Supplementary Table 5 – The top 50 annotated DMR between cerebellum and cortex. Values correspond to normalized MeDIP-seq read-counts across each feature location. Data for all annotated features is available for download from http://epigenetics.iop.kcl.ac.uk/brain.

Location	Feature Type	Gene	Cerebellum	Cortical	Mean	CV
11:77202308-77202370	CDS	RSF1	0.00	138.56	69.28	1.39
7:154861384-154863384	Shore	AC008060.7	189.46	1.02	95.24	1.38
7:154860615-154862615	Shore	AC008060.7	136.11	0.98	68.55	1.37
2:236784717-236784996	Intragenic CGI	AC079135.6	0.00	57.46	28.73	1.37
19:60562204-60562413	Intergenic CGI	FAM71E2	0.00	52.87	26.43	1.36
16:85999046-85999180	CDS	AC010531.8	0.00	47.95	23.98	1.36
12:131382416-131382659	Intragenic CGI	GALNT9	0.00	44.29	22.15	1.35
5:146235539-146237539	Shore	PPP2R2B	91.93	1.07	46.50	1.35
14:56351720-56353720	Shore		98.30	1.30	49.80	1.35
11:82211137-82211244	CDS	AP001646.5	669.95	15.78	342.86	1.35
9:21445722-21445937	CDS	AL353732.14	0.00	38.17	19.08	1.34
4:6165398-6165601	Intragenic CGI	JAKMIP1	0.00	37.97	18.99	1.34
17:23598597-23598662	CDS	PPY2.	0.00	37.79	18.89	1.34
9:13313112-13313350	Intergenic CGI		0.00	37.65	18.83	1.34
3:27740200-27740679	Intergenic CGI	EOMES	90.70	1.41	46.05	1.34
16:4671562-4671836	Intragenic CGI	MGRN1	0.00	36.79	18.40	1.34
1:191416047-191417315	CDS	CDC73	75.96	1.04	38.50	1.34
1:2266218-2266517	Intragenic CGI	MORN1	0.00	36.11	18.05	1.34
7:140761670-140762610	CDS	AC005692.1	103.32	1.96	52.64	1.34
12:124144301-124144541	Intragenic CGI	AACS	0.00	33.45	16.73	1.33
14:56348869-56350869	Shore		133.97	2.97	68.47	1.33
3:124646905-124648905	Shore	ADCY5	117.26	2.50	59.88	1.33
7:100259231-100259451	Intragenic CGI	EPHB4	0.00	32.11	16.05	1.33
9:129329182-129329411	Intragenic CGI	FAM129B	0.00	31.87	15.94	1.33
12:52375478-52375680	Intergenic CGI		78.23	1.42	39.83	1.33
1:67924858-67926858	Shore	GADD45A	160.01	3.98	82.00	1.33
1:245486141-245486278	CDS	VN1R5	0.00	30.64	15.32	1.33

X:13864071-13866071	Shore	GPM6B	77.39	1.53	39.46	1.33
14:56348040-56350040	Shore		112.43	2.75	57.59	1.32
2:204011-204245	Intergenic CGI	SH3YL1	0.00	29.25	14.62	1.32
6:34111529-34112205	Intragenic CGI	GRM4	0.00	28.64	14.32	1.32
7:4136007-4136279	Intragenic CGI	SDK1	0.00	28.18	14.09	1.32
5:58371638-58373638	Shore	PDE4D	114.21	2.96	58.59	1.32
19:16039396-16039609	Intergenic CGI	TPM4	0.00	28.02	14.01	1.32
7:829041-829326	Intragenic CGI		0.00	27.84	13.92	1.32
11:1905491-1905764	Intragenic CGI	TNNT3	0.00	27.58	13.79	1.32
7:77813545-77814824	LCP	MAGI2	86.37	2.06	44.21	1.32
19:51938589-51940589	Shore	STRN4	153.03	4.52	78.77	1.32
22:48103509-48103925	Intergenic CGI		0.00	26.87	13.43	1.32
5:58368593-58370593	Shore	PDE4D	163.28	4.91	84.09	1.32
22:48986742-48987006	Intragenic CGI	AL022328.21	0.00	26.52	13.26	1.32
15:87744857-87746857	Shore		67.15	1.49	34.32	1.31
10:47199711-47199934	Intergenic CGI	AL603965.10	0.00	26.32	13.16	1.31
11:1915510-1915823	Intragenic CGI	TNNT3	0.00	25.97	12.98	1.31
12:101868528-101868770	Intergenic CGI		0.00	25.95	12.98	1.31
11:17757602-17757853	Intragenic CGI	AC124056.8	0.00	25.29	12.64	1.31
15:28045787-28047787	Shore		121.35	3.68	62.51	1.31
1:226540458-226540666	Intragenic CGI	OBSCN	0.00	25.01	12.50	1.31
16:87497865-87498100	Intragenic CGI	CBFA2T3	0.00	24.89	12.44	1.31
11:108800193-108802193	Shore	AP000775.4	152.80	5.03	78.92	1.31

Supplementary Table 6: Across brain region (cortex vs cerebellum) DMRs show a strong enrichment for functions involved in brain development and neuronal function. Shown are functional pathways identified by IPA analysis as being significantly enriched in the list of genes differentially methylated between cortex and cerebellum.

Functional Annotation	p-Value	Molecules	Number
neurogenesis	1.76E-19	ADAM10, ADCYAP1, AGT, AKT1, APLP2, ARHGEF1, ARHGEF10, ARTN, ATOH1, AXIN1, B4GALNT1, BAIAP2, BCAN, BHLHE22, BRSK2, CABLES1, CACNA1A, CACNA1H, CD44, CD9, CELSR2, CLU, CSNK1D, CTNNA2, CTNND2, CUX1, DAGLA, DBX1, DNER, DNMT3B, DRGX, DSCAM, EBF1, EBF3, EDN3, EFNB2, EMX1, EN2, ENAH, EPHA4, FABP7, FGA, FGF3, FGFR2, FGFR3, GAB1, GDF7, GET4, GFRA2, GFRA3, GNAQ, GPR6, HELT, HOXB3, ID1, ID4, JAG1, JAG2, KALRN, KCNMA1, KCTD11, KIF5C, KNDC1, LMO4, LRRK2, MAPK8IP3, MAPT, MARK2, MDK, MEIS1, METRN, MRAP, MYC, MYCN, NELF, NFATC2, NFATC4, NFIX, NGFR, NOS2, NOTCH1, NPTX1, NR2E1, NR2F1, NR4A2, NRCAM, NRTN, NTN1, NUMBL, OTX2, PAK4, PALLD, PAX6, PAX7, PLXNA3, PLXNB2, PMP22, POU3F2, POU4F1, PPP2R2C, PPP2R2D, PRDM16, PRDM8, PRKCA, PTPRF, RHOQ, RUNX3, RXRA, SEMA4C, SERPINF2, SGK223, SHC3, SLC1A2, SLIT1, SOX1, SOX9, SPR, SRC, SYNGR1, TBR1, TGFBR1, THRB, TIAM1, TIMP2, TLX1, TNK2, TUBB3, VAV2, VCAM1, VIM, VSX1, ZIC1, ZNF274, ZNF536	134
guidance of neurites	2.11E-12	ADAM10, ADCYAP1, APBB2, ARHGEF1, ARTN, ATOH1, BAIAP2, BCAN, CABLES1, CACNA1A, CD9, CDH4, CSNK1D, CTNND2, DNER, DRGX, EFNB2, EPHA4, EPHB3, FGF3, FGFR3, GAB1, GAS1, GDF7, GFRA2, GFRA3, GNAQ, GPR6, KALRN, KIF5C, KNDC1, LRRK2, MAPK8IP3, MAPT, MARK2, MDK, NEFH, NELF, NFATC2, NFATC4, NFIX, NGFR, NOTCH1, NPTX1, NRCAM, NRTN, NTN1, OTX2, PAK4, PALLD, PAX6, PLXNA1, PLXNA3, PLXNB1, POU3F2, POU4F1, PPP2R2C, PPP2R2D, PRKCA, PTPRF, PVRL1, RGMA, RHOQ, RUNX3, SERPINF2, SGK223, SLIT1, SPON2, SRC, SYNGR1, TBR1, TIAM1, TNK2, UNC5A, VAV2, VCAM1, VIM	77
development of brain	3.75E-09	ABR, APLP2, ATOH1, BCAN, CACNA1A, CBS, CTNNA2, CXCL3, DSCAML1, EBF3, EMX1, EN2, EOMES, EPHA4, FGFR2, FGFR3, GAS1, GDF7, HHEX, HSPG2, ID4, IRS2, LHX5, MAPK8IP3, MDGA1, MSX1, MYO16, NCOR2, NOS2, NOTCH1, NR1H2, NR2E1, NR2F1, NR2F2, NTN1, NUMBL, OTX2, PAX6, PLXNA3, POU3F2, PTPRS, SDF4, SHROOM2, SLC1A2, SLIT1, SMARCA4, SOX1, SRC, TBR1, UNC5C, ZEB2, ZIC1, ZIC2, ZIC5	54
differentiation of neurons	3.89E-08	ADCYAP1, AKT1, ATOH1, BHLHE22, BRSK2, CUX1, DNMT3B, DRGX, EBF1, EBF3, EDN3, EMX1, EN2, FGFR2, FGFR3, GDF7, GET4, HELT, ID4, JAG1, KCNMA1, KNDC1, MAPK8IP3, MAPT, MDK, MEIS1, METRN, MYC, NGFR, NOTCH1, NR2E1, NR4A2, OTX2, PAK4, PAX6, PLXNB2, POU3F2, POU4F1, PRKCA, SHC3, SLC1A2, SOX1, SRC, TBR1, TIMP2, TLX1, TUBB3, VSX1, ZNF536	49
neurological process of	5.56E-08	ADCY5, ADCYAP1, AGT, APBA3, BSN, CACNA1A, CACNA1H, CAST, CCK, CCKBR, CHRNA4,	67

cells		CNTNAP1, DAGLA, DLG2, DLGAP1, DNAJC5, DPYSL4, FABP7, FGFR2, FGFR3, FSTL1, GABRD, GAD2, GFRA2, GFRA3, GLP1R, GNA11, GNAQ, GRIA2, GRIK5, GRIN2B, GRM2, GRM4, ID1, JAM3, JPH3, KCNAB1, KCNMA1, KCNN3, KCNQ1, KCNQ2, KCNQ3, KISS1, MTNR1B, NCS1, NFATC4, NGFR, NMNAT3, NOTCH1, NPTX1, NPY, NRTN, NRXN2, OTX2, PAX6, PMP22, PPP2R2C, PRKAR1B, PRKCA, PRKCZ, PROC, SLC17A7, SLC1A2, SNPH, SRC, SYNGR1, VIPR1	
growth of neurites	1.73E-07	ADAM10, ADCYAP1, ARHGEF1, ARTN, BAIAP2, BCAN, CABLES1, CACNA1A, CD9, CSNK1D, CTNND2, DNER, EFNB2, FGF3, FGFR3, GAB1, GFRA2, GNAQ, GPR6, KALRN, KNDC1, LRRK2, MAPT, MARK2, MDK, NELF, NFATC2, NFATC4, NFIX, NGFR, NOTCH1, NPTX1, NRCAM, NRTN, NTN1, PAK4, PALLD, PLXNA3, POU3F2, POU4F1, PPP2R2C, PPP2R2D, PTPRF, RHOQ, SERPINF2, SGK223, SLIT1, SRC, SYNGR1, TIAM1, TNK2, VAV2, VCAM1, VIM	54
neurological process of neurons	2.05E-07	ADCY5, ADCYAP1, AGT, APBA3, BSN, CACNA1A, CAST, CCK, CCKBR, CHRNA4, DAGLA, DLG2, DLGAP1, DNAJC5, DPYSL4, FABP7, FSTL1, GABRD, GAD2, GFRA2, GFRA3, GLP1R, GRIA2, GRIK5, GRIN2B, GRM2, GRM4, JPH3, KCNMA1, KCNN3, KCNQ2, KCNQ3, KISS1, MTNR1B, NCS1, NFATC4, NGFR, NMNAT3, NOTCH1, NPTX1, NPY, NRTN, NRXN2, OTX2, PAX6, PMP22, PRKAR1B, PRKCA, PRKCZ, PROC, SLC17A7, SLC1A2, SNPH, SRC, SYNGR1, VIPR1	56
guidance of axons	2.80E-07	APBB2, ARTN, ATOH1, CDH4, DRGX, EPHA4, EPHB3, GAS1, GDF7, GFRA3, KIF5C, MAPK8IP3, NGFR, NRCAM, NTN1, OTX2, PAX6, PLXNA3, PTPRF, PVRL1, RUNX3, SLIT1, SPON2, TBR1, UNC5A	25
migration of neurons	5.90E-07	ADAM10, ADCYAP1, APBB2, ATOH1, CCK, DRGX, EBF1, EBF3, EFNB2, GFRA3, MDK, NAV1, NFIX, NGFR, NR2F1, NR2F2, NR4A2, NTN1, PAK4, PAX6, PIP5K1C, PLXNB2, POU3F2, POU4F1, SDC3, SEMA4C, SLC1A2, SLIT1, SOX1, TBR1, TIAM1	31
auditory system development	1.52E-06	ABR, AKT1, ATOH1, ATP6V1B1, CELSR1, FGF3, FGFR2, FGFR3, FRZB, FZD4, GAS1, JAG1, JAG2, KCNQ1, MSX1, MYC, NKX3-2, NOTCH1, NTN1, OTX2, PRRX2, ROR2, SHROOM2, TBX1, THRB	25
development of neurons	3.28E-06	ADCYAP1, ATOH1, DAGLA, EBF3, EN2, ENAH, FABP7, FGFR2, FGFR3, GFRA2, GFRA3, NGFR, NOTCH1, NR2F1, NR4A2, NRTN, OTX2, PAK4, PAX6, PMP22, POU4F1, SPR, SRC, TLX1, VSX1	25
quantity of neurons	4.21E-06	ADCYAP1, APLP2, ATOH1, CTSD, EN2, FGFR2, GFRA2, HELT, JAG1, JAG2, KIF5C, MAPT, NELF, NGFR, NHLH1, NKX2-8, NOTCH1, NR2F1, NR4A2, NRTN, NTN1, OTX2, PAX6, POU4F1, PRKAR1B, RUNX3, SHC3, SOX9, TBR1, TH	30
outgrowth of neurites	8.23E-06	ADAM10, ADCYAP1, BAIAP2, BCAN, CABLES1, CD9, CSNK1D, DNER, EFNB2, FGF3, FGFR3, GAB1, GFRA2, GNAQ, GPR6, LRRK2, MAPT, MARK2, MDK, NELF, NFATC2, NFATC4, NFIX, NGFR, NPTX1, NRCAM, NRTN, NTN1, PAK4, PALLD, PLXNA3, POU4F1, PPP2R2C, PTPRF, RHOQ, SERPINF2, SGK223, SRC, SYNGR1, TIAM1, TNK2, VAV2, VCAM1, VIM	44
synaptic transmission	1.32E-05	APBA3, BSN, CACNA1A, CACNB4, CAST, CCK, CCKBR, CHRNA4, DLG2, DLGAP1, DNAJC5, FSTL1, GABRD, GAD2, GRIA2, GRIK5, GRIN2B, GRM2, GRM4, HCN4, KCNMA1, KCNQ2, KCNQ3, KISS1, MTNR1B, NCS1, NPTX1, NPY, NRXN2, PMP22, PRKCZ, RAB3A, SHC3, SLC17A7, SLC1A2, SNPH, SRC, TH, VIPR1	39

neurotransmission	1.36E-05	APBA3, BSN, CACNA1A, CACNB4, CAST, CCK, CCKBR, CHRNA4, DLG2, DLGAP1, DNAJC5, FSTL1, GABRD, GAD2, GNA11, GNAQ, GRIA2, GRIK5, GRIN2B, GRM2, GRM4, HCN4, KCNMA1, KCNQ1, KCNQ2, KCNQ3, KISS1, MTNR1B, NCS1, NPTX1, NPY, NRXN2, PMP22, PRKCZ, RAB3A, SHC3, SLC17A7, SLC1A2, SNPH, SRC, TH, VIPR1	42
development of forebrain	1.48E-05	APLP2, EBF3, EPHA4, FGFR3, GDF7, HHEX, MAPK8IP3, MSX1, NCOR2, NOTCH1, NR2E1, NR2F1, NR2F2, NTN1, NUMBL, OTX2, PAX6, PTPRS, SLC1A2, SLIT1, SMARCA4, SOX1, SRC, ZIC1, ZIC5	25
synaptic transmission of neurons	2.00E-05	APBA3, BSN, CACNA1A, CAST, CCK, CCKBR, DLG2, DLGAP1, DNAJC5, FSTL1, GABRD, GAD2, GRIA2, GRIK5, GRIN2B, GRM2, GRM4, KCNMA1, KCNQ2, KCNQ3, KISS1, MTNR1B, NCS1, NPTX1, NPY, NRXN2, PMP22, PRKCZ, SLC17A7, SLC1A2, SNPH, VIPR1	32
synaptic transmission of cells	2.00E-05	APBA3, BSN, CACNA1A, CAST, CCK, CCKBR, DLG2, DLGAP1, DNAJC5, FSTL1, GABRD, GAD2, GRIA2, GRIK5, GRIN2B, GRM2, GRM4, KCNMA1, KCNQ2, KCNQ3, KISS1, MTNR1B, NCS1, NPTX1, NPY, NRXN2, PMP22, PRKCZ, SLC17A7, SLC1A2, SNPH, SRC, VIPR1	33
neurotransmission of nervous tissue	5.02E-05	APBA3, BSN, CACNA1A, CAST, CCK, CCKBR, CHRNA4, DLG2, DLGAP1, DNAJC5, FSTL1, GABRD, GAD2, GRIA2, GRIK5, GRIN2B, GRM2, GRM4, KCNMA1, KCNQ2, KCNQ3, KISS1, MTNR1B, NCS1, NPTX1, NPY, NRXN2, PMP22, PRKCZ, RAB3A, SLC17A7, SLC1A2, SNPH, VIPR1	34
development of ear	6.14E-05	ABR, AKT1, ATOH1, ATP6V1B1, CELSR1, FGF3, FGFR2, FGFR3, FRZB, FZD4, GAS1, JAG1, KCNQ1, OTX2, PRRX2, ROR2, SHROOM2, TBX1, THRB	19
development of cerebellum	8.49E-05	ATOH1, CACNA1A, CBS, EN2, FGFR3, GAS1, LHX5, MYO16, OTX2, PAX6, PTPRS, SDF4, SRC, ZIC1	14
development of rhombencephalon	9.69E-05	ATOH1, CACNA1A, CBS, EN2, FGFR3, GAS1, LHX5, MYO16, OTX2, PAX6, PTPRS, SDF4, SMARCA4, SRC, TBR1, ZIC1	16
development of cerebral cortex	1.11E-04	ATOH1, BCAN, CACNA1A, EMX1, ID4, LHX5, NOS2, NR2E1, NTN1, OTX2, PAX6, PLXNA3, POU3F2, PTPRS, SRC, ZEB2	16
loss of neurons	1.26E-04	ADCYAP1, CAST, CTSD, DBX1, GFRA2, MAPT, NGFR, NOS2, NR1H2, NR4A2, NRTN, POU4F1, SHC3, SLC1A2, THRB, VAC14	16
development of ganglion cells	1.53E-04	GFRA2, NGFR, NRTN, PMP22, POU4F1	5
formation of axons	1.71E-04	ARHGEF1, MARK2, METRN, NGFR, PAK4, PAX6, PLXNB1, PLXNB2, TIAM1	9
development of nerves	2.03E-04	ARHGEF10, B4GALNT1, CD44, DRGX, EPHA4, FGA, FGFR2, HOXB3, LMO4, NGFR, NRTN, THRB	12
outgrowth of axons	2.64E-04	MAPT, MARK2, NELF, NFATC2, NFATC4, NFIX, NGFR, NTN1, PAK4, PLXNA3, VIM	11
development of inner ear	3.73E-04	ABR, ATOH1, ATP6V1B1, CELSR1, FGF3, FGFR2, FGFR3, FRZB, JAG1, KCNQ1, OTX2, PRRX2, ROR2, TBX1, THRB	15
development of dopaminergic neurons	3.95E-04	FGFR2, FGFR3, NR4A2, OTX2	4

development of hippocampus	4.37E-04	BCAN, CACNA1A, ID4, LHX5, NOS2, NR2E1, NTN1, PLXNA3, POU3F2, PTPRS, ZEB2	11
neuritogenesis of cells	6.05E-04	ADCYAP1, CACNA1H, FGFR2, GFRA2, NRTN, PPP2R2C, PRKCA, SRC	8
proliferation of neuroblasts	6.96E-04	ARTN, GFRA3, ID1, ID4, MYCN, NOTCH1, NUMBL, PAX6, PLXNB2, SEMA4C, ZIC1	11
memory	7.27E-04	ADCYAP1, ATP1A3, CREBBP, CYP46A1, GABRB3, GABRD, GLP1R, GRIA2, GRIN2B, ITGA5, JPH3, KCNAB1, KCNH3, MAPT, NOS2, NOTCH1, NPY, PRKCZ, SLC17A7, SLC24A2, TH, TNFRSF1B	22
outgrowth of sensory axons	7.53E-04	NFATC2, NFATC4, NTN1, PLXNA3	4
development of trigeminal ganglion neurons	8.32E-04	GFRA2, NRTN, POU4F1	3
differentiation of bipolar cells	8.32E-04	PLXNB2, TBR1, VSX1	3
quantity of spinal cord cells	8.32E-04	NGFR, NRTN, PRKAR1B	3
growth of axons	8.89E-04	ARHGEF1, ARTN, CACNA1A, MAPT, MARK2, NELF, NFATC2, NFATC4, NFIX, NGFR, NTN1, PAK4, PLXNA3, POU3F2, POU4F1, VIM	16
neurological process of mice	9.04E-04	ABCA2, ADCY5, BDKRB1, CREBBP, CTNNA2, CYP46A1, FABP7, GABRB3, GAD2, GFRA2, JPH3, KCNAB1, KCNC1, KCNH3, KCNQ2, MAPT, NGFR, NOS2, NOTCH1, NR4A2, NRTN, PAX6, PDE4D, PTPRN2, RAB3A, TBX1, TH, THRB, TNFRSF1B	29
morphogenesis of middle ear	1.17E-03	GAS1, MSX1, MYC, NKX3-2, PRRX2, TBX1	6
formation of neurites	1.30E-03	ARHGEF1, MAPT, MARK2, METRN, NFIX, NGFR, NRCAM, PAK4, PAX6, PLXNB1, PLXNB2, RHOQ, TIAM1, TNK2	14
innervation	1.36E-03	GFRA2, GFRA3, GRIA2, NGFR, NR2F1, NRTN, POU4F1, VCAM1	8
development of mesencephalon	1.69E-03	APLP2, EN2, FGFR2, GDF7, MSX1, NTN1, OTX2	7
quantity of hair cells	2.05E-03	ATOH1, JAG1, JAG2, NOTCH1	4
dendritic growth/branching	2.07E-03	ADCYAP1, BSN, CTNNA2, CTNND2, DPYSL4, EPHB3, KALRN, KNDC1, MAGI2, NEFH, NGFR, NOTCH1, NUMBL, PALM, PTPRF, SLIT1	16
morphogenesis of dendrites	2.31E-03	CACNA1A, CELSR2, CTNNA2, DSCAM, KNDC1, NR2E1, NUMBL, PTPRF	8
innervation of nervous	2.64E-03	GFRA2, GFRA3, NR2F1, NRTN, POU4F1	5

tissue			
formation of neurons	3.09E-03	ATOH1, GDF7, NOTCH1, OTX2, PAX6, TBR1	6
morphogenesis of inner ear	3.28E-03	ABR, ATOH1, ATP6V1B1, CELSR1, FGFR2, FRZB, OTX2, PRRX2, ROR2, TBX1	10
branching of neurites	3.32E-03	ADCYAP1, BSN, CTNNA2, CTNND2, DPYSL4, EPHB3, FNBP1, KALRN, KNDC1, MAGI2, NEFH, NGFR, NOTCH1, NTN1, NUMBL, PALM, PTPRF, ROR2, SLIT1	19
differentiation of Cajal-Retzius neurons	3.62E-03	PLXNB2, TBR1	2
formation of subventricular zone	3.62E-03	NR2E1, PAX6	2
generation of projection neurons	3.62E-03	NR2E1, PAX6	2
quantity of dorsal root ganglion cells	3.62E-03	NGFR, NRTN	2
differentiation of amacrine cells	3.80E-03	EBF1, EBF3, NR4A2	3
innervation of cells	4.38E-03	GFRA2, GFRA3, GRIA2, NRTN	4
depolarization of postsynaptic neurons	4.49E-03	GRIA2, GRIK5, GRIN2B, GRIN2C, GRM2	5
proliferation of neuronal cells	5.21E-03	ADCYAP1, ARTN, GFRA3, ID1, ID4, MAPT, MYCN, NOTCH1, NR2E1, NUMBL, PAX6, PDGFB, PLXNB2, SEMA4C, ZIC1	15
migration of neuroblasts	5.69E-03	EFNB2, NFIX, NGFR, PLXNB2, SEMA4C	5
development of commissure	6.03E-03	EPHA4, MAPK8IP3, NR2E1, NTN1	4
morphology of dendritic spines	6.03E-03	CTNNA2, DPYSL4, EPHB3, MAGI2	4
projection of axons	6.03E-03	NGFR, NR2F1, NTN1, TBR1	4

Supplementary Table 7 – A high proportion of genes located near cerebellum-cortex DMRs are differentially expressed in the two tissues. Expression values are normalized and log2 transformed.

MeDIP-seq DMR	DMR rank	Associated gene(s)	Illumina HT-12 v4 Probe ID	Frontal cortex expression	Cerebellum expression	Mean difference	P-value
11:77202308-77202370	1	RSF1	1400246	6.68	6.76	-0.08	2.91E-03
2:236784717-236784996	4	ASB18	1190048	6.47	6.50	-0.03	3.45E-02
19:60562204-60562413	5	FAM71E2	1690731	6.53	6.52	0.02	NS
16:85999046-85999180	6	ZCCHC14	2710402	7.97	8.71	-0.73	1.97E-16
12:131382416-131382659	7	GALNT9	5340136	10.05	10.49	-0.45	7.83E-07
			5360056	6.79	7.18	-0.39	1.38E-11
			6980553	7.24	7.60	-0.37	9.49E-10
5:146235539-146237539	8	PPP2R2B	620369	8.49	8.54	-0.04	NS
			1990019	7.92	7.06	0.86	3.82E-14
			4010176	6.48	6.50	-0.02	NS
			6020523	11.38	9.98	1.40	8.30E-22
			6860564	6.43	6.42	0.02	1.05E-03
4:6165398-6165601	12	JAKMIP1	3870075	6.46	6.45	0.01	NS
3:27740200-27740679	15	EOMES	7320372	6.46	9.25	-2.79	1.90E-33
16:4671562-4671836	16	MGRN1	1510414	7.59	7.58	0.02	NS
1:191416047-191417315	17	B3GALT2	4590008	7.63	6.62	1.01	1.41E-23
1:2266218-2266517	18	MORN1	2030564	6.49	7.03	-0.54	9.76E-15
12:124144301-124144541	20	AACS	1580504	7.23	6.91	0.32	3.39E-11
3:124646905-124648905	22	ADCY5	1030187	6.55	6.50	0.05	2.35E-05
7:100259231-100259451	23	EPHB4	1260730	6.44	6.44	0.00	NS
9:129329182-129329411	24	FAM129B	3180053	7.54	8.52	-0.98	5.81E-23
			4070711	6.87	7.18	-0.31	9.57E-09
1:67924858-67926858	26	GADD45A	670255	7.05	6.70	0.36	4.19E-09

			4880673	7.55	7.06	0.49	3.52E-10
1:245486141-245486278	27	VN1R5	7650332	6.50	6.50	0.00	NS
X:13864071-13866071	28	GPM6B	2630279	10.35	9.50	0.85	2.29E-06
			3870202	12.21	11.46	0.75	9.20E-12
			5340465	7.73	7.21	0.52	2.80E-08
14:56348040-56350040	29	OTX2	1820541	6.42	6.99	-0.57	1.72E-08
			3460546	6.44	6.47	-0.03	5.19E-03
2:204011-204245	30	SH3YL1	2570288	7.70	7.44	0.26	1.35E-03
6:34111529-34112205	31	GRM4	6840255	6.79	9.78	-2.99	1.39E-34
19:16039396-16039609	34	TPM4	5910524	6.80	7.33	-0.52	1.86E-16
			6860441	6.59	7.21	-0.62	1.40E-20
7:829041-829326	35	UNC84A	4390113	7.52	8.39	-0.87	2.86E-13
7:77813545-77814824	37	STRN4	1770048	7.06	6.71	0.35	6.72E-16
			4290044	6.58	6.53	0.05	2.96E-04
		FKRP	2230189	6.89	7.11	-0.22	4.64E-04
			3520521	6.79	6.57	0.21	1.30E-13
5:58368593-58370593	40	SELO	7570437	7.09	7.94	-0.84	2.78E-20
12:101868528-101868770	45	KCNC1	4880114	6.47	6.52	-0.06	3.62E-06
15:28045787-28047787	47	OBSCN	1690040	6.54	6.94	-0.40	6.74E-14
1:226540458-226540666	48	CBFA2T3	630053	6.53	6.76	-0.23	1.88E-04
			2070427	6.44	6.46	-0.02	1.62E-02

Supplementary Table 8 - The top 50 within-cortex TS-DMRs. Values correspond to normalized MeDIP-seq read-counts across each feature location. (* The list is filtered to include features with a mean score > 10 to minimize noise).

Location	Feature	Gene	Ent Ctx	Frontal Ctx	STG	Vis Ctx	Ctx Mean*	CV
11:89320429-89320673	Intergenic CGI		3.06	37.82	8.48	0.00	12.34	1.30
11:119866524-119866616	Genebody	AP000758.5	30.85	11.83	0.00	0.00	10.67	1.25
15:94688797-94689034	Intergenic CGI		29.59	3.12	8.72	0.00	10.36	1.17
7:4842550-4842755	Intragenic CGI	AC092610.10	10.93	31.39	3.24	0.00	11.39	1.14
1:2442655-2442867	Intragenic CGI	PANK4	5.56	10.39	3.31	42.75	15.51	1.11
5:176793468-176793704	Intragenic CGI	GRK6	12.24	29.09	3.15	0.00	11.12	1.08
X:47581036-47581308	Promoter CGI		9.25	5.05	0.00	29.19	10.87	1.08
16:32594416-32594919	Genebody	AC138907.2	6.26	5.11	3.39	31.62	11.59	1.06
1:1434451-1434712	Intergenic CGI	ATAD3A	11.32	12.41	9.34	60.83	23.47	1.02
7:577390-577603	Intragenic CGI	PRKAR1B	5.11	17.69	1.74	37.24	15.45	0.98
17:75529234-75529477	Intragenic CGI	TBC1D16	18.41	36.22	1.53	4.67	15.21	0.98
17:76551754-76552009	Intragenic CGI	AC127496.5	6.11	25.35	9.71	0.00	10.29	0.96
1:3145020-3145221	Intragenic CGI	PRDM16	3.40	9.73	3.68	28.18	11.25	0.95
12:31660564-31660767	Intergenic CGI		10.41	30.18	8.93	0.00	12.38	0.95
21:43465251-43465453	Intragenic CGI	CRYAA	4.01	11.25	3.28	28.04	11.65	0.91
15:94699050-94701050	Intergenic Shore		28.47	4.06	10.43	3.98	11.74	0.91
15:94699146-94701146	Intergenic Shore		28.27	4.06	10.24	3.98	11.64	0.91
10:134590791-134591023	Intergenic CGI	C10orf93	7.36	23.99	1.43	39.08	17.97	0.90
X:47227948-47228232	Genebody	CXorf24	15.16	10.50	9.13	51.92	21.68	0.90
19:5025591-5025814	Intragenic CGI	JMJD2B	7.23	11.60	4.79	35.57	14.80	0.89
8:684469-684697	Intergenic CGI		9.27	35.82	9.24	4.97	14.82	0.89
15:94701648-94703648	Intergenic Shore		25.75	5.46	5.20	5.69	10.52	0.88
15:94698305-94700305	Intergenic Shore		26.42	3.87	10.04	3.98	11.08	0.88
1:1016240-1016488	Intragenic CGI	C1orf159	7.52	25.39	4.32	4.57	10.45	0.88
2:105252327-105252560	Intragenic CGI	TGFBRAP1	9.62	23.15	7.46	0.00	10.06	0.87
19:13226922-13227158	Intragenic CGI	CACNA1A	35.54	21.59	9.41	0.00	16.64	0.87
19:52627295-52627527	Intragenic CGI	SLC8A2	19.68	21.91	4.63	0.00	11.55	0.87

7:2026433-2026657	Intragenic CGI	MAD1L1	5.14	12.57	1.65	25.30	11.16	0.86
19:8372976-8373180	Intragenic CGI	RAB11B	5.96	14.50	1.63	27.76	12.46	0.86
2:11668217-11668523	Intragenic CGI	AC110754.4	16.17	39.52	10.15	3.71	17.39	0.85
14:103639252-103639533	Intragenic CGI	AL136001.6	15.96	21.43	4.99	0.00	10.60	0.85
17:77954903-77955440	Intragenic CGI	AC132938.9	11.50	32.07	5.24	6.35	13.79	0.84
14:102500287-102500540	Intragenic CGI	CDC42BPB	15.60	25.13	6.86	0.00	11.90	0.84
12:131375309-131375579	Intragenic CGI	GALNT9	9.67	28.75	6.59	4.20	12.30	0.84
1:21802766-21802967	Intragenic CGI	RAP1GAP	27.77	24.35	7.37	0.00	14.87	0.84
X:44901921-44902122	Intragenic CGI	CXorf36	5.10	24.23	3.50	33.81	16.66	0.84
10:133890188-133890408	Intragenic CGI	STK32C	15.84	21.01	0.00	5.15	10.50	0.84
X:70204615-70205084	Promoter CGI	SNX12	8.03	9.87	4.74	31.49	13.53	0.84
8:605143-605352	Intragenic CGI	ERICH1	5.34	11.28	3.54	27.10	11.81	0.84
13:22320687-22320896	Intergenic CGI		18.91	21.08	5.31	0.00	11.33	0.83
16:82556385-82556640	Intragenic CGI	OSGIN1	13.06	39.31	6.96	8.89	17.06	0.83
1:2836916-2837148	Intergenic CGI		3.22	14.18	5.89	29.31	13.15	0.83
20:3162696-3162927	Intragenic CGI	SLC4A11	21.52	21.73	6.08	0.00	12.33	0.83
5:172319499-172319708	Intragenic CGI	RPL26L1	10.85	6.37	3.17	27.10	11.87	0.83
7:55776206-55776495	Intergenic CGI		4.73	32.85	5.87	15.70	14.79	0.82
X:47815579-47816087	Promoter CGI		8.22	5.92	5.11	26.84	11.52	0.82
19:45882089-45882300	Intragenic CGI	NUMBL	10.92	25.61	3.32	5.37	11.30	0.82
X:48974153-48974464	Intragenic CGI	CACNA1F	10.44	23.26	5.94	47.43	21.77	0.82
19:47238487-47238736	Intragenic CGI	GRIK5	16.12	31.15	1.49	9.11	14.47	0.82
22:19044640-19046114	Genebody	AC007731.16	7.79	29.57	5.38	8.49	12.81	0.82

Supplementary Table 9 - Across cortex DMRs are significantly enriched for functions involved in neurogenesis and nervous system function. Shown are functional pathways identified by IPA analysis on the most 500 most variably-methylated features across different cortical regions.

Functional Annotation	p-Value	Molecules	Number
development of central nervous system	3.83E-08	CELSR1, FGFR3, FTO, GRIK1, HSPG2, IGF1R, IRX6, NOTCH1, NOTCH3, NPTX1, NR1H2, NUMBL, OTX1, SHROOM2, SMARCA4, TBR1, VAX1, ZIC1	18
development of brain	7.74E-08	FGFR3, FTO, HSPG2, IGF1R, IRX6, NOTCH1, NOTCH3, NR1H2, NUMBL, OTX1, SHROOM2, SMARCA4, TBR1, VAX1, ZIC1	15
development of forebrain	6.15E-07	FGFR3, FTO, IRX6, NOTCH1, NOTCH3, NUMBL, OTX1, SMARCA4, ZIC1	9
development of nervous system	6.66E-07	AKT1, CELSR1, FGFR3, FTO, GRIK1, HDAC4, HSPG2, IGF1R, IRX6, KCNQ2, NOTCH1, NOTCH3, NPTX1, NR1H2, NUMBL, OTX1, PRPS1, SHROOM2, SMARCA4, TBR1, VAX1, ZIC1	22
Neurogenesis	6.29E-05	CACNA1A, CACNA1F, FGFR3, GALNS, KNDC1, MAG, NOTCH1, NOTCH3, NUMBL, PRDM16, TSC2, VAX1, ZIC1	13
turnover of synaptic vesicles	1.28E-04	AGRN, CACNA1A	2
morphology of brain	1.62E-04	FTO, GAK, IRX6	3
expansion of neurons	4.76E-04	FTO, GET4, IRX6	3
proliferation of neuroblasts	5.42E-04	NOTCH1, NUMBL, TSC2, VAX1, ZIC1	5
quantity of neuroglia	7.33E-04	FGFR3, GALNS, NOTCH1, SOX9, TSC2	5
quantity of oligodendrocytes	8.67E-04	FGFR3, NOTCH1, SOX9	3
neurological process of axons	9.41E-04	CDH4, CSPG4, GALNS, KIF5C, MAG, TBR1, UNC5A, VAX1	8
branching of dendrites	9.65E-04	AGRN, KNDC1, NOTCH1, PALM	4
morphogenesis of dendrites	9.65E-04	CACNA1A, CACNA1F, KNDC1, NUMBL	4
retraction of axons	1.25E-03	GALNS, OTX1	2
quantity of astrocytes	1.42E-03	GALNS, SOX9, TSC2	3
growth of neurons	1.52E-03	FTO, GET4, IRX6, MAG	4
generation of neurons	1.66E-03	FTO, IRX6, NOTCH1, TBR1	4
synaptic transmission of neurons	1.96E-03	AGRN, CACNA1A, CACNA1G, GRIK1, GRIK5, KCNQ2, NPTX1, NTSR2, PRKCZ	9
neurological process of cells	1.97E-03	AGRN, CACNA1A, CACNA1G, CACNA1H, FGFR3, GALNS, GRIK1, GRIK5, GRIN2D, KCNQ2, NOTCH1, NPTX1, NTSR2, PRKAR1B, PRKCZ	15
neurological process of	2.36E-03	AGRN, CACNA1A, CACNA1G, CACNA1H, FGFR3, GALNS, GRIK1, GRIK5, KCNQ2, NOTCH1,	14

eukaryotic cells		NPTX1, NTSR2, PRKAR1B, PRKCZ	
neurological process of CA1 neuron	2.44E-03	GRIK1, PRKAR1B, PRKCZ	3
synaptic transmission	3.79E-03	AGRN, CACNA1A, CACNA1G, GRIK1, GRIK5, GRIN2D, KCNQ2, NPTX1, NTSR2, PRKCZ	10
guidance of axons	3.95E-03	CDH4, GALNS, KIF5C, TBR1, UNC5A, VAX1	6
neurological process of normal cells	4.25E-03	AGRN, CACNA1A, CACNA1G, FGFR3, GALNS, GRIK1, GRIK5, KCNQ2, NOTCH1, NPTX1, NTSR2, PRKAR1B, PRKCZ	13
axonogenesis	4.65E-03	CACNA1A, CSPG4, MAG	3
differentiation of neurons	5.20E-03	AGRN, AKT1, BRSK2, FGFR3, GALNS, GET4, KNDC1, NOTCH1, NOTCH3, TBR1	10
quantity of hair cells	5.42E-03	JAG2, NOTCH1	2
morphogenesis of forebrain	7.83E-03	FTO, IRX6	2
quantity of neurons	7.88E-03	JAG2, KIF5C, NOTCH1, PRKAR1B, SOX9, TBR1, VAX1	7

Supplementary Table 10 – The top 50 CGI TS-DMRs across blood, cerebellum and cortex. Values correspond to normalized MeDIP-seq read-counts across each feature location. Data for all annotated features is available for download from http://epigenetics.iop.kcl.ac.uk/brain.

Location	CGI type	Gene	Blood	Cerebellum	Cortical	Mean	CV
19:4987959:4988220	Intragenic	JMJD2B	241.20	1.39	6.94	83.18	1.63
12:52375478:52375680	Intergenic		0.00	78.23	1.42	26.55	1.62
9:2231337:2231659	Intergenic		140.17	0.00	4.57	48.25	1.62
8:67617099:67617545	Intergenic		210.51	0.81	8.99	73.44	1.60
8:125808866:125810447	Promoter	MTSS1	59.95	0.77	1.12	20.61	1.58
14:56348462:56348869	Intergenic		0.00	44.35	1.03	15.13	1.57
4:40447448:40447653	Intragenic	NSUN7	88.00	1.76	2.31	30.69	1.57
8:143855281:143856413	Intergenic	LYNX1	45.34	0.00	1.41	15.58	1.55
7:27121524:27121951	Intragenic	AC010990.3	77.31	0.00	3.88	27.06	1.55
6:33156394:33156792	Intragenic	HLA-DPB1	110.80	1.04	5.36	39.07	1.55
1:120707494:120707919	Intergenic	AL357493.8	109.89	2.56	3.99	38.81	1.55
2:171538156:171538954	Intergenic		39.64	0.00	1.17	13.60	1.54
3:50332457:50332687	Intragenic	HYAL2	188.57	0.00	13.76	67.44	1.54
1:146248690:146249097	Intergenic		111.98	3.96	3.29	39.74	1.54
7:27130344:27130623	Intragenic	AC010990.3	145.43	3.03	7.42	51.96	1.53
17:19712201:19712406	Promoter	ULK2	146.14	3.78	6.74	52.22	1.53
14:61348790:61349092	Intergenic		67.15	1.37	2.45	23.66	1.53
1:37795039:37795306	Promoter	DNALI1	120.60	4.07	4.39	43.02	1.53
11:30562134:30562376	Intergenic	MPPED2	155.53	1.71	10.05	55.76	1.52
15:54812639:54813442	Promoter	SUHW4.	82.61	2.46	2.96	29.34	1.52
19:1311038:1311347	Intragenic	MUM1	196.12	1.17	14.60	70.63	1.52
14:53488427:53488631	Intragenic	BMP4	135.31	3.79	6.49	48.53	1.52
6:26333365:26333769	Promoter	HIST1H3E	170.49	1.79	12.08	61.45	1.51
2:2752407:2752625	Intergenic		143.53	0.00	11.99	51.84	1.51
17:73548669:73548918	Intragenic		126.57	4.85	5.27	45.56	1.51
19:19590127:19590814	Promoter	PBX4	86.18	2.20	4.10	30.83	1.51
2:119485613:119485866	Intergenic		56.12	0.00	3.50	19.87	1.51

9:115900800:115901412	Promoter	KIF12	90.74	2.75	4.04	32.51	1.50
12:93104787:93105005	Intragenic	PLXNC1	112.75	0.00	9.38	40.71	1.50
13:99440775:99441095	Intergenic	ZIC2	0.00	0.00	19.18	6.39	1.50
13:112725628:112725837	Intragenic	MCF2L	118.43	1.73	8.48	42.88	1.49
19:18180145:18180363	Intragenic	PDE4C	31.33	0.00	1.31	10.88	1.49
10:88285185:88285572	Intergenic		80.11	2.94	3.45	28.83	1.49
7:27116555:27116943	Intragenic	HOXA3	44.24	1.07	1.62	15.64	1.49
5:42980023:42980339	Intergenic		57.00	0.00	4.12	20.37	1.49
12:56416095:56416628	Intragenic	CENTG1	57.59	0.80	3.34	20.58	1.49
16:29892165:29892960	Promoter	AC093512.2	39.01	0.99	1.18	13.73	1.49
11:128065313:128065747	Intergenic	FLI1	96.26	0.84	7.47	34.85	1.49
10:43138080:43138610	Intergenic		36.92	0.78	1.19	12.96	1.49
13:83351665:83351898	Intragenic	SLITRK1	29.07	0.00	1.17	10.08	1.48
8:19504185:19504417	Intragenic	AC116376.4	29.19	0.00	1.19	10.13	1.48
19:3995917:3996154	3' end	AC016586.9	116.92	1.53	9.05	42.50	1.48
3:27740200:27740679	Intergenic	EOMES	6.35	90.70	1.41	32.82	1.48
17:14142451:14142777	Intergenic	HS3ST3B1	94.23	2.38	5.73	34.11	1.48
9:98488931:98489399	Intergenic		41.55	0.00	2.55	14.70	1.48
11:27698635:27698849	Intragenic	BDNF	35.76	1.93	0.00	12.56	1.48
4:657273:657601	Intragenic	ATP5I	29.66	0.00	1.30	10.32	1.48
7:27112594:27113125	3' end	HOXA3	97.50	5.32	3.29	35.37	1.48
19:15429027:15430227	Intragenic	AC011492.8	57.39	0.66	3.83	20.63	1.47
9:35780823-35781149	Intergenic	NPR2	43.68	0.00	3.00	15.56	1.47

Supplementary Table 11: Cross-tissue variable CGIs show a strong enrichment for functions involved in tissue development, nervous system function, and hematopoiesis. Shown are the top 25 biological functions identified by IPA analysis on the most variable CGIs.

Functional Annotation	p-value	Molecules	Number
activation of DNA	5.46E-16	ABL1, ABLIM1, ABT1, AKAP12, AKT1, APBA2, APBB1IP, APLP2, ARHGEF17, ATXN1, BANP, BARHL2, BCL3, BDNF, BMP4, BMP7, BRCA1, CACNA1H, CARM1, CCND1, CD247, COMP, CREBBP, CRTC1, CTBP1, CTBP2, CTF1, CUX1, DAXX, DNMT3A, DVL3, EBF1, EDNRB, EEF1D, EHMT1, ELL, EN1, ETS1, FASN, FLI1, FLII, FOXA2, FOXA3, FOXJ1, FOXK1, FOXK2, FOXO3, FOXO4, FOXP4, GFI1, GRIN1, GTF2H4, GZF1, HDAC4, HELT, HHEX, HIF3A, HNF1B, HOXA1, HOXA5, HOXA9, HOXB1, HOXB7, HOXB9, HOXD13, HYAL2, ID4, IGF2BP1, INSR, JAK3, JARID2, KDM6B, KLF13, KLF16, KLF6, LCK, LPIN1, LRP1, MAP4K1, MBD3 (includes EG:17192), MDFI, MKL1, MLL2, MLLT1, MPL, MTA1, MYF6, NAD+, NCOR2, NEUROD2, NFATC1, NFIC, NFIX, NKX2-3, NOLC1, NOS3, NR2E1, NR2F1, NR5A1, OGT, PAX6, PBX1, PDPK1, PER3, PGR, PIAS4, PITX2, POLR2F, POU4F1, PPP2R5C, PRDM16, PRKCZ, PRMT2, PTBP1, PTGFRN, RAC1, RBAK, RBM14, RGMA, RTKN, RUNX1, RUNX3, S100A1, SGK1, SIM1 (includes EG:20464), SKAP1, SMAD7, SMARCA2, SMARCC2, SOX8, SPEN, SPHK2, SPI1, SSBP3, TAF1, TAF4, TBKBP1, TCF25, TEF, TFAP2D, TGFB1, THRA, TOLLIP, TRRAP, TSC2, TXNRD1, UBE2I, USF2, VAV2, WNT5A, WT1, ZBTB7A, ZFPM1, ZIC3, ZNF238, ZNF536	156
tissue development	8.02E-16	AACS, ABL1, ABLIM1, ABR, AKT1, APLP2, ARX, BAIAP2, BARHL2, BCL3, BDNF, BMP4, BMP7, BRCA1, BRD4, CACNA1H, CALCA, CALCB, CARM1, CCND1, CD247, CD47, CDH23, CIT, CNP, CNTNAP1, COL4A1, COL5A1, COMP, CREBBP, CTBP1, CTBP2, CTF1, CTGF, CUX1, DEF6, DHH, DMD, DNMT3A, DVL3, EBF1, EBF3, EDNRB, EIF2C2, EN1, EOMES, EPHA2, EPHB4, F12, FGF19, FLI1, FOXA2, FOXB1, FOXP4, FURIN, GAA, GAMT, GBX2, GFI1, GNA11, GNPDA1, GPC3, GRIN1, GRM4, GZF1, HDAC4, HHEX, HHIP, HIF3A, HNF1B, HOXA1, HOXA3, HOXA4, HOXA5, HOXA9, HOXB1, HOXB3, HOXC4, HOXD13, ID4, IGF1R, IGF2BP1, IGFBP4, IGSF10, INSR, JAK3, JARID2, KCNA3, KCNQ1, KLF6, KNDC1, LAMA4, LFNG, LHX6, LRPAP1, LSR, LY6E, MAG, MAP7, MAPK8IP3, MAPT, MBD3 (includes EG:17192), MBP, MBTPS1, MEIS1, MKL1, MPL, MTA1, MYF6, NCOR2, NDEL1, NEUROD2, NFATC1, NFIC, NFIX, NKX2-3, NOS2, NOS3, NPR2, NR2E1, NR2F1, NR5A1, NRN1, NRTN, PACSIN1, PAX6, PBX1, PDPK1, PDPN, PEX7, PHOX2B, PIP5K1C, PITX2, POU4F1, PPP1CA, PRKAR1B, PRKCZ, PRMT1, PTPRS, RAB3A, RAC1, ROR2, RUNX3, S1PR4, SDF4, SGK1, SHROOM2, SIM1 (includes EG:20464), SLITRK1, SMAD7, SMTN, SOX8, SPHK2, SPI1, TAF4, TBX15, TGFB1, THRA, TRIO, TSC2, TSHZ3, TULP1, UNC5C, USH1C, WNT10A, WNT5A, WT1, ZBTB7A, ZFP36L1, ZFPM1, ZIC2, ZIC3	172
activation of DNA endogenous promoter	9.16E-16	ABL1, ABLIM1, ABT1, AKT1, ATXN1, BARHL2, BCL3, BDNF, BMP4, BMP7, BRCA1, CREBBP, CRTC1, CTBP1, CUX1, DAXX, DNMT3A, EDNRB, EHMT1, EN1, ETS1, FOXA2, FOXA3, FOXJ1, FOXK1, FOXK2, FOXO3, FOXO4, FOXP4, GFI1, GRIN1, GTF2H4, GZF1, HDAC4, HELT, HHEX, HIF3A, HNF1B, HOXA1, HOXA5, HOXB1, HOXB9, HOXD13, ID4, JAK3, JARID2, KDM6B, KLF13, KLF16, KLF6, LPIN1, MBD3	102

		(includes EG:17192), MDFI, MKL1, MLL2, MLLT1, MYF6, NCOR2, NEUROD2, NFATC1, NFIC, NFIX, NKX2-3, NR2E1, NR2F1, NR5A1, PAX6, PBX1, PER3, PGR, PIAS4, PITX2, POLR2F, POU4F1, PRDM16, PRKCZ, RAC1, RBM14, RUNX1, RUNX3, S100A1, SKAP1, SMAD7, SMARCA2, SMARCC2, SOX8, SPI1, SSBP3, TAF1, TAF4, TCF25, TEF, TGFB1, THRA, UBE2I, USF2, WNT5A, WT1, ZBTB7A, ZFPM1, ZIC3, ZNF238	
differentiation	1.10E-15	ABL1, AKT1, ARX, BAIAP2, BARHL2, BCL3, BDNF, BMP4, BMP7, BRCA1, BRSK1, BRSK2, BSG, CALCA, CAND2, CARD11, CARM1, CBFA2T3, CCND1, CD1D, CD47, CDH23, CIT, COL4A1, CR1L, CREBBP, CTBP1, CTBP2, CTF1, CTGF, CUX1, DEF6, DHH, DPYSL4, EBF1, EBF3, EDNRB, EN1, EOMES, EPHA2, EPHB4, ETS1, FAM20C, FLI1, FLNC, FOXA2, FOXA3, FOXJ1, FOXO3, FOXO4, GET4, GFI1, GNA11, GPC3, HDAC4, HELT, HHEX, HNF1B, HOXA1, HOXA5, HOXA9, HOXB1, HOXB7, HSPA2, ICAM5, ID4, IGF1R, INSR, ITGAL, JAK3, KLF13, KLF6, KNDC1, LAMA4, LAMP1, LCK, LIMK2, LPIN1, LY6E, MAD1L1, MAGI2, MAP4K1, MAPK8IP3, MAPT, MBP, MDFI, MEIS1, MKL1, MLF1, MLLT1, MPL, MTA1, MYF6, NCOR2, NEUROD2, NFATC1, NFIC, NKX2-3, NOS2, NOS3, NR2E1, NR2F1, NR5A1, NRTN, OGT, OPRM1, PACSIN1, PALM, PAX6, PBX1, PDPK1, PGR, PHOX2B, PIK3CD, PIK3R2, PITX2, PLXNB2, POU4F1, PPP1CA, PRDM16, PRKCZ, PRKX, RAC1, RASA3, RND2, RNF128, ROR2, RUNX1, RUNX3, S1PR4, SBF1, SEMA4C, SLC9A3, SMAD7, SMARCA2, SOX8, SPI1, SPN, TBX15, TGFB1, THRA, TRAPPC9, TSC2, TSHZ3, TXNRD1, UBE2I, UHRF2, ULK2, USH1C, WNT5A, WT1, ZBTB7A, ZFPM1, ZIC2, ZIC3, ZNF238, ZNF536	157
expression of DNA	4.82E-15	ABCG1, ABL1, ABLIM1, ABT1, AFF3, AKAP12, AKT1, APBB1IP, ARHGEF17, ATXN1, BANP, BARHL2, BCL3, BDNF, BMP4, BMP7, BRCA1, CACNA1H, CALCA, CARM1, CCND1, CD247, COMP, CREBBP, CRTC1, CTBP1, CTBP2, CTF1, CUX1, CUX2, CXXC1, DAXX, DMD, DNMT3A, DVL3, EBF1, EBF3, EBF4, EDNRB, EEF1D, EHMT1, ELL, EN1, ETS1, FASN, FGR, FLI1, FLII, FOXA2, FOXA3, FOXD2, FOXJ1, FOXK1, FOXK2, FOXO3, FOXO4, FOXP4, GBX2, GFI1, GRIN1, GTF2H4, GZF1, HDAC4, HELT, HHEX, HIF3A, HNF1B, HOXA1, HOXA5, HOXA9, HOXB1, HOXB7, HOXB9, HOXD13, HYAL2, ID4, IGF2BP1, INSR, JAK3, JARID2, KCNQ1, KDM6B, KLF11, KLF13, KLF16, KLF6, LCK, LPIN1, LRP1, MAP3K11, MBD3 (includes EG:17192), MDFI, MEIS1, MKL1, MLF1, MLL2, MLLT1, MPL, MTA1, MTERF, MYF6, NAD+, NCOR2, NEUROD2, NFATC1, NFIC, NFIX, NKX2-3, NOLC1, NOS3, NR2E1, NR2F1, NR5A1, OGT, PAX6, PBX1, PDPK1, PER3, PGR, PHOX2B, PIAS4, PITX2, POLR2F, POU4F1, PPP2R5C, PRDM16, PRKCZ, PRMT2, PTBP1, PTGFRN, RAC1, RBAK, RBM14, RGMA, RSF1, RTKN, RUNX1, RUNX3, S100A1, SBNO2, SGK1, SIM1 (includes EG:20464), SKAP1, SMAD7, SMARCA2, SMARCC2, SOX8, SPEN, SPI1, SSBP3, TAF1, TAF4, TBKBP1, TCF25, TEF, TFAP2D, TGFB1, THRA, TOLLIP, TRRAP, TSC2, TSHZ3, UBE2I, USF2, VAV2, WDR75, WNT5A, WT1, XAB2, ZBTB7A, ZFPM1, ZGPAT, ZIC3, ZNF219, ZNF238, ZNF274, ZNF282, ZNF536	178
differentiation of cells	6.58E-15	ABL1, AKT1, ARX, BAIAP2, BARHL2, BCL3, BDNF, BMP4, BMP7, BRCA1, BRSK1, BRSK2, BSG, CALCA, CAND2, CARD11, CARM1, CBFA2T3, CCND1, CD1D, CD47, CDH23, CIT, COL4A1, CR1L,	148

		CREBBP, CTBP1, CTBP2, CTF1, CTGF, CUX1, DEF6, DHH, EBF1, EBF3, EDNRB, EN1, EOMES, EPHA2, EPHB4, ETS1, FAM20C, FLI1, FLNC, FOXA2, FOXA3, FOXJ1, FOXO3, FOXO4, GET4, GFI1, GNA11, GPC3, HDAC4, HELT, HHEX, HNF1B, HOXA1, HOXA5, HOXA9, HOXB1, HOXB7, HSPA2, ID4, IGF1R, INSR, ITGAL, JAK3, KLF13, KNDC1, LAMA4, LAMP1, LCK, LIMK2, LPIN1, LY6E, MAD1L1, MAP4K1, MAPK8IP3, MAPT, MBP, MDFI, MEIS1, MKL1, MLF1, MLLT1, MPL, MYF6, NCOR2, NEUROD2, NFATC1, NFIC, NKX2-3, NOS2, NOS3, NR2E1, NR2F1, NR5A1, NRTN, OGT, PACSIN1, PAX6, PBX1, PDPK1, PGR, PHOX2B, PIK3CD, PIK3R2, PITX2, PLXNB2, POU4F1, PPP1CA, PRDM16, PRKCZ, PRKX, RAC1, RASA3, RNF128, ROR2, RUNX1, RUNX3, S1PR4, SBF1, SEMA4C, SLC9A3, SMAD7, SMARCA2, SOX8, SPI1, SPN, TBX15, TGFB1, THRA, TRAPPC9, TSC2, TSHZ3, TXNRD1, UBE2I, UHRF2, USH1C, WNT5A, WT1, ZBTB7A, ZFPM1, ZIC2, ZIC3, ZNF238, ZNF536	
transcription of DNA	7.07E-15	ABCG1, ABL1, ABLIM1, ABT1, AFF3, AKAP12, AKT1, APBB1IP, ARHGEF17, ATXN1, BANP, BARHL2, BCL3, BDNF, BMP4, BMP7, BRCA1, CACNA1H, CALCA, CARM1, CCND1, CD247, COMP, CREBBP, CRTC1, CTBP1, CTBP2, CTF1, CUX1, CUX2, CXXC1, DAXX, DMD, DNMT3A, DVL3, EBF1, EBF3, EBF4, EDNRB, EEF1D, EHMT1, ELL, EN1, ETS1, FASN, FGR, FLI1, FLII, FOXA2, FOXA3, FOXD2, FOXJ1, FOXK1, FOXK2, FOXO3, FOXO4, FOXP4, GBX2, GFI1, GRIN1, GTF2H4, GZF1, HDAC4, HELT, HHEX, HIF3A, HNF1B, HOXA1, HOXA5, HOXA9, HOXB1, HOXB7, HOXB9, HOXD13, HYAL2, ID4, IGF2BP1, INSR, JAK3, JARID2, KDM6B, KLF11, KLF13, KLF16, KLF6, LCK, LPIN1, LRP1, MAP3K11, MBD3 (includes EG:17192), MDFI, MEIS1, MKL1, MLF1, MLL2, MLLT1, MPL, MTA1, MTERF, MYF6, NAD+, NCOR2, NEUROD2, NFATC1, NFIC, NFIX, NKX2-3, NOLC1, NOS3, NR2E1, NR2F1, NR5A1, OGT, PAX6, PBX1, PDPK1, PER3, PGR, PHOX2B, PIAS4, PITX2, POLR2F, POU4F1, PRDM16, PRKCZ, PRMT2, PTBP1, PTGFRN, RAC1, RBAK, RBM14, RGMA, RSF1, RTKN, RUNX1, RUNX3, S100A1, SBNO2, SGK1, SIM1 (includes EG:20464), SKAP1, SMAD7, SMARCA2, SMARCC2, SOX8, SPEN, SPI1, SSBP3, TAF1, TAF4, TBKBP1, TCF25, TEF, TFAP2D, TGFB1, THRA, TOLLIP, TRRAP, TSC2, TSHZ3, UBE2I, USF2, VAV2, WDR75, WNT5A, WT1, XAB2, ZBTB7A, ZFPM1, ZGPAT, ZIC3, ZNF219, ZNF238, ZNF274, ZNF282, ZNF536	176
transcription	1.26E-14	ABCG1, ABL1, ABLIM1, ABT1, AFF3, AKAP12, AKT1, AP2A2, APBB1IP, ARHGEF17, ATXN1, BANP, BARHL2, BCL3, BDNF, BMP4, BMP7, BRCA1, BRF1 (includes EG:2972), CACNA1H, CALCA, CARM1, CCND1, CD247, COMP, CREBBP, CRTC1, CTBP1, CTBP2, CTF1, CUX1, CUX2, CXXC1, DAXX, DMD, DNMT3A, DVL3, EBF1, EBF3, EBF4, EDNRB, EEF1D, EHMT1, ELL, EN1, ETS1, FASN, FGR, FLI1, FLII, FOXA2, FOXA3, FOXD2, FOXJ1, FOXK1, FOXK2, FOXO3, FOXO4, FOXP4, GBX2, GFI1, GRIN1, GTF2H4, GZF1, HDAC4, HELT, HHEX, HIF3A, HNF1B, HOXA1, HOXA5, HOXA9, HOXB1, HOXB7, HOXB9, HOXD13, HYAL2, ID4, IGF2BP1, INSR, JAK3, JARID2, KDM6B, KLF11, KLF13, KLF16, KLF6, LCK, LPIN1, LRP1, MAGI2, MAP3K11, MBD3 (includes EG:17192), MDFI, MEIS1, MKL1, MLF1, MLL2, MLLT1, MPL, MTA1, MTERF, MYF6, NAD+, NCOR2, NEUROD2, NFATC1, NFIC, NFIX, NKX2-3, NOLC1, NOS3, NR2E1, NR2F1, NR5A1, OGT, PAX6, PBX1, PDPK1, PER3, PGR, PHOX2B, PIAS4, PITX2,	180

		POLR2F, POU4F1, PRDM16, PRKCZ, PRMT2, PSMB10, PTBP1, PTGFRN, RAC1, RBAK, RBM14, RGMA, RSF1, RTKN, RUNX1, RUNX3, S100A1, SBNO2, SGK1, SIM1 (includes EG:20464), SKAP1, SMAD7, SMARCA2, SMARCC2, SOX8, SPEN, SPI1, SSBP3, TAF1, TAF4, TBKBP1, TCF25, TEF, TFAP2D, TGFB1, THRA, TOLLIP, TRRAP, TSC2, TSHZ3, UBE2I, USF2, VAV2, WDR75, WNT5A, WT1, XAB2, ZBTB7A, ZFPM1, ZGPAT, ZIC3, ZNF219, ZNF238, ZNF274, ZNF282, ZNF536	
developmental process of organism	2.89E-14	AACS, ABL1, ABLIM1, ABR, AFF3, AKT1, APBA2, APLP2, ARX, ATP6V0C, BCL3, BDNF, BMP4, BMP7, BRCA1, BRD4, CALCA, CAMK2B, CARM1, CATSPER1, CCND1, CD247, CDH23, CIT, COL4A1, COL5A1, COMP, CR1L, CREBBP, CSNK1D, CTBP1, CTBP2, CTF1, CTGF, CUX1, CXXC1, DAXX, DHH, DNALI1, DNMT3A, DVL3, EBF3, EDNRB, EHMT1, ELL, EN1, EOMES, EPHA2, EPHB4, ETS1, FBN2, FGF19, FLI1, FLII, FOXA2, FOXB1, FOXJ1, FOXK1, FOXP4, FURIN, GAA, GAMT, GBX2, GFI1, GNA11, GNPDA1, GPC3, GRIN1, GZF1, HELT, HHEX, HHIP, HIF3A, HNF1B, HOXA1, HOXA3, HOXA4, HOXA5, HOXA9, HOXB1, HOXB3, HOXB7, HOXB9, HOXC4, HOXD13, HOXD4, ID4, IDH3G, IGF1R, IGF2BP1, IGFBP4, INSR, JAK3, JARID2, KCNQ1, KLF6, LFNG, LSR, LY6E, MAP7, MAPK8IP3, MAPT, MBD3 (includes EG:17192), MDFI, MEIS1, MLL2, MYF6, NCOR2, NDEL1, NEUROD2, NFATC1, NFIC, NKX2-3, NOS2, NOS3, NR2E1, NR2F1, NR5A1, PAX6, PBX1, PDPK1, PDPN, PIP5K1C, PITX2, PLXNA1, PLXNB2, POU4F1, PPP1CA, PRKAR1B, PRKCZ, PRMT1, PTPRS, RAB3A, RAC1, RGMA, ROR2, RUNX3, S1PR4, SDF4, SF1, SHROOM2, SHROOM3, SIM1 (includes EG:20464), SMAD7, SOX8, SPHK2, SPI1, SSBP3, TAF4, TGFB1, THRA, TRIO, TSC2, TSHZ3, TXNRD1, UNC5C, USF2, USH1C, WHSC2, WNT10A, WNT5A, WT1, XAB2, ZFPM1, ZIC2, ZIC3	166
neurogenesis	9.88E-14	ABL1, AKT1, APBA2, APLP2, ARX, BAIAP2, BARHL2, BDNF, BMP4, BMP7, BRSK1, BRSK2, CACNA1H, CD47, CDH23, CIT, CNP, CSNK1D, CTF1, CUX1, DHH, EBF1, EBF3, EN1, EPHA2, FOXA2, FOXB1, FZR1, GET4, GFI1, GRIN1, HELT, HHIP, HOXA1, HOXA3, HOXB1, HOXB3, ICAM5, ID4, IGF1R, KCNA3, KIAA1279, KNDC1, LFNG, LHX6, MAG, MAOA, MAPK8IP3, MAPT, MARK2, MEIS1, NDEL1, NELF, NEUROD2, NFIX, NOS2, NR2E1, NR2F1, NRTN, PACSIN1, PAX6, PBX1, PHOX2B, PLXNB2, POU4F1, PRDM13, PRDM16, PRDM8, RAC1, RUNX3, S1PR4, SEMA4C, SGK1, SHC2, SPHK2, SYN1, TGFB1, THRA, TNK2, TRIO, TSC2, ULK2, USH1C, VAV2, WNT5A, WT1, ZIC3, ZNF274, ZNF536	89
organogenesis	3.14E-13	AACS, ABL1, ABLIM1, ABR, AKT1, APLP2, ARX, BCL3, BDNF, BMP4, BMP7, BRD4, CARM1, CCND1, CD247, CDH23, COL5A1, COMP, CTBP1, CTBP2, CTGF, CUX1, DHH, DNMT3A, DVL3, EBF3, EDNRB, EN1, EOMES, EPHA2, EPHB4, FGF19, FLI1, FOXA2, FOXB1, FOXJ1, FOXP4, FURIN, GAA, GAMT, GBX2, GFI1, GNA11, GPC3, GRIN1, GZF1, HHEX, HHIP, HIF3A, HNF1B, HOXA1, HOXA3, HOXA5, HOXA9, HOXB3, HOXC4, HOXD13, ID4, IGF1R, IGF2BP1, INSR, JAK3, JARID2, KCNQ1, KLF6, LFNG, LSR, LY6E, MAPK8IP3, MAPT, MEIS1, NCOR2, NDEL1, NEUROD2, NFATC1, NFIC, NKX2-3, NOS2, NOS3, NR2E1, NR2F1, NR5A1, PAX6, PBX1, PDPK1, PDPN, PIP5K1C, PITX2, PPP1CA, PRKAR1B, PRKCZ, PTPRS, RAB3A, RAC1, ROR2, RUNX3, S1PR4, SDF4, SHROOM2, SIM1 (includes EG:20464), SMAD7, SOX8, SPHK2, SPI1, TAF4, TGFB1, THRA, TRIO, TSC2, TSHZ3, UNC5C, USH1C, WNT10A,	118

		WNT5A, WT1, ZFPM1, ZIC2, ZIC3	
development of organ	1.13E-12	AACS, ABL1, ABLIM1, ABR, AKT1, APLP2, ARX, BCL3, BDNF, BMP4, BMP7, BRD4, CCND1, CD247, CDH23, COL5A1, COMP, CTBP1, CTBP2, CTGF, CUX1, DHH, DNMT3A, DVL3, EBF3, EDNRB, EN1, EOMES, EPHA2, EPHB4, FGF19, FLI1, FOXA2, FOXB1, FOXP4, FURIN, GAA, GAMT, GBX2, GFI1, GNA11, GPC3, GRIN1, GZF1, HHEX, HHIP, HIF3A, HNF1B, HOXA1, HOXA3, HOXA5, HOXA9, HOXB3, HOXC4, HOXD13, ID4, IGF1R, IGF2BP1, INSR, JAK3, JARID2, KCNQ1, KLF6, LFNG, LSR, LY6E, MAPK8IP3, MAPT, MEIS1, NCOR2, NDEL1, NEUROD2, NFATC1, NKX2-3, NOS2, NOS3, NR2E1, NR2F1, NR5A1, PAX6, PBX1, PDPK1, PDPN, PIP5K1C, PITX2, PPP1CA, PRKAR1B, PRKCZ, PTPRS, RAB3A, RAC1, ROR2, RUNX3, S1PR4, SDF4, SHROOM2, SIM1 (includes EG:20464), SMAD7, SOX8, SPHK2, SPI1, TAF4, TGFB1, THRA, TRIO, TSC2, TSHZ3, UNC5C, USH1C, WNT10A, WNT5A, WT1, ZFPM1, ZIC2, ZIC3	115
genetic disorder	1.57E-11	AACS, ABCB6, ABCC5, ABCG1, ABL1, ABLIM1, ABTB2, ACCN4, ACTN4, ADAMTS13, ADARB2, ADCY9, AFAP1, AFF3, AGAP1, AHNAK, AKAP12, AKAP8, AKT1, ALDH5A1, ALPK1, ALS2CR11, ANKRD44, AP2A2, APBA2, APLP2, ARHGEF17, ARX, ATP6AP2, ATXN1, AUTS2, BAI3, BAIAP2, BANP, BCL3, BDNF, BEX4, BMP4, BMP7, BNIP3, BRCA1, BRD1, BRSK1, BRSK2, BSG, C10orf107, C11orf41, C17orf57, C1QL3, C9orf25, C9orf3, C9orf86, CACNA1H, CALCA, CALCB, CAMK2B, CAND2, CAPN1, CARD11, CBFA2T3, CC2D1A, CCDC27, CCND1, CD1D, CD247, CD47, CD58, CDH23, CELF4, CFP, CIT, CKAP4, CKM, CLCN1, CLCN6, CLSTN1, CLYBL, CNNM1, CNP, CNTNAP1, COL4A1, COL5A1, COMP, CPEB3, CPT1B, CREBBP, CTBP2, CTGF, CTIF, CUX1, CUX2, CXXC5, CYP1A1, DCDC2, DCUN1D2, DDX25, DDX39B, DEF8, DFFB, DGKA, DHH, DHX40, DIP2C, DMD, DNMT3A, DYNC1H1, EBF1, EBF3, EBF4, EDNRB, EEF2, EFR3B, EHMT1, EIF2C2, ENO2, EPHA2, EPHB4, F12, FAM125B, FAM19A5, FAM20C, FASN, FBN2, FCHO1, FGF19, FLCN, FLI1, FLII, FLNC, FMNL1, FOXA2, FOXJ1, FOXK1, FOXO3, FOXP4, FURIN, FZR1, GAA, GCK, GDPD2, GFI1, GLA, GLRA3, GNA11, GNG7, GPC3, GPC4, GPC5, GPD1, GPR123, GPR50, GPRC5C, GPX4, GRAMD1B, GRIN1, GRIN2C, GRM2, GRM4, GUCA1A, HDAC4, HHIP, HIF3A, HIST1H3A (includes others), HLA-DPB1, HLA-DRB5, HNF1B, HOXA1, HOXA3, HOXA5, HOXA9, HOXB3, HOXC4, HOXD13, HSPA2, HYAL2, ICAM5, ID4, IFT140, IGF1R, IGFBP4, INPP5A, INSR, IQCE, ITGAL, JAK3, JARID2, KCNA1, KCNA3, KCNC3, KCNC4, KCND2, KCNIP4, KCNK9, KCNQ1, KCNQ2, KDM2B, KDM4B, KIAA0146, KIAA0907, KIAA1279, KIF1B, KLF11, KLF13, KLF16, KLF6, KNDC1, KRBA1, LAMA4, LAMP1, LCK, LETM1, LFNG, LMF1, LPIN1, LRP1, LRPAP1, LY6E, MAD1L1, MAG, MAGI2, MAN1C1, MAN2B1, MAOA, MAP3K11, MAP4K1, MAPK8IP3, MAPT, MBNL2, MBP, MBTPS1, MCF2L, MDFI, MEIS1, METTL15, MGAT4B, MGRN1, MKL1, MKNK2, MLC1, MLF1, MLL2, MLLT1, MPL, MPPED2, MSRA, MT1E, MT1M, MT1X, MTA1, MTAP, MUM1, MVD, MYF6, MYH14, MYO1D, MYO1F, NAV1, NCOR2, NDUFS7, NEDD4L, NELF, NFIC, NFIX, NHLRC1, NKX2-3, NMNAT2, NOL4, NOS2, NOS3, NPLOC4, NPR2, NR2E1, NR2F1, NR5A1, NRTN, OAZ1, OGFR, OGT, OLFM2, OLFML2B, OPRD1, OPRM1, PACSIN2, PAH, PAX6, PBX1, PCDHA1, PCDHB7, PCDHGA3,	420

		PCNT, PDE4C, PDE6B, PDPK1, PENK, PER3, PEX7, PGAM2, PGR, PHOX2B, PIK3CD, PIP5K1C, PITX2, PKHD1L1, PKLR, PLEK2, PLEKHA6, PLXNA1, PLXNC1, PMS2, POLG, PON1, PPFIA3, PPP2R5C, PRDM16, PRKCZ, PRKX, PRLHR, PRRC2A, PRRT1, PSMB10, PTCHD1, PTGFRN, PTGIS, PTPRG, PTPRN2, PTRF, RAB3A, RAB40B, RAC1, RIMS1, RNF220, ROR2, RPL39, RPS9, RSF1, RUNX1, RUNX3, S100A1, S100A11, S1PR4, SAT1, SBNO2, SDF4, SEC31B, SETBP1, SF1, SFSWAP, SGK1, SH3PXD2A, SH3YL1, SHROOM2, SHROOM3, SIM1 (includes EG:20464), SKAP1, SLC25A6, SLC2A9, SLC7A1, SLC7A5, SLC8A3, SLITRK1, SMARCA2, SMTN, SMU1, SPG7, SPI1, SPTLC1, SSBP3, ST14, STXBP1, SULT1A2, SULT2B1, SUN1, SYN1, SYP, TAF1, TAOK2, TBCD, TBX15, TGFB1, THRA, TMCO3, TMEM47, TNRC6C, TRAPPC9, TRIO, TSC2, TSHZ3, TSNARE1, TSSC1, TTLL11, TUBB1, TULP1, UBE2I, UHRF2, ULK2, UNC5C, UPP1, USH1C, USP20, USP42, VAV2, VGLL4, WNK4, WNT10A, WNT5A, WT1, ZBTB46, ZFAT, ZFP36L1, ZGPAT, ZIC2, ZIC3, ZMYND8, ZNF274, ZNF454, ZNF490, ZNF536, ZNF608, ZNF613, ZNF649	
developmental process of neurons	3.63E-11	ABL1, AKT1, ARX, BARHL2, BDNF, BMP4, BMP7, BRSK1, BRSK2, CDH23, CIT, CNP, CTF1, CUX1, DHH, EBF1, EBF3, EN1, EPHA2, FOXA2, GET4, GFI1, HELT, HOXA1, HOXB1, ID4, KCNA3, KNDC1, LHX6, MAG, MAPK8IP3, MAPT, MEIS1, NEUROD2, NR2E1, NR2F1, NRTN, PACSIN1, PAX6, PBX1, PHOX2B, PLXNB2, POU4F1, RAC1, S1PR4, TGFB1, THRA, USH1C, WNT5A, ZIC3, ZNF536	51
differentiation of neurons	3.78E-11	ABL1, AKT1, ARX, BARHL2, BDNF, BMP4, BMP7, BRSK1, BRSK2, CDH23, CIT, CTF1, CUX1, EBF1, EBF3, EN1, EPHA2, FOXA2, GET4, GFI1, HELT, HOXA1, HOXB1, ID4, KNDC1, MAPK8IP3, MAPT, MEIS1, NR2E1, PACSIN1, PAX6, PBX1, PHOX2B, PLXNB2, POU4F1, RAC1, S1PR4, TGFB1, THRA, USH1C, WNT5A, ZIC3, ZNF536	43
neurological disorder	3.97E-11	AACS, ABCC5, ABL1, ABLIM1, ABR, ABTB2, ADARB2, ADCY9, AGAP1, AKAP12, AKT1, ALDH5A1, APBA2, APLP2, ARX, ATP6AP2, ATXN1, AUTS2, BAI3, BAIAP2, BANP, BCL3, BDNF, BEX4, BRCA1, BRD1, BRSK1, BTN1A1, C11orf41, C17orf57, CACNA1H, CALCA, CAMK2B, CAPN1, CC2D1A, CCDC27, CCND1, CD1D, CD247, CD47, CD58, CDH23, CELF4, CIT, CKAP4, CLCN6, CLK2, CLYBL, CNNM1, CNP, CNTNAP1, COL4A1, CPT1B, CREBBP, CTBP2, CTGF, CTIF, CUX2, CYP1A1, DCDC2, DCUN1D2, DDX25, DEF8, DIP2C, DMD, DYNC1H1, EBF1, EBF3, EDNRB, EEF2, EFR3B, EHMT1, ENO2, EOMES, EPHA2, EPHB4, FAM19A5, FASN, FMNL1, FOXK1, FOXO3, FURIN, FZR1, GAA, GDPD2, GFI1, GNA11, GNG7, GPC3, GPC4, GPC5, GPD1, GPR50, GPRC5C, GPX4, GRIN1, GRIN2C, GRM2, GRM4, HCN2, HDAC4, HIST1H3A (includes others), HNF1B, HOXA1, HOXA3, HOXA9, HSPA2, ICAM5, ID4, IGF1R, INPP5A, INSR, IQCE, ITGAL, JARID2, KCNA1, KCNC3, KCNC4, KCND2, KCNIP4, KCNQ1, KCNQ2, KDM4B, KIAA0907, KIAA1279, KIF1A, KIF1B, KLF13, KLF16, KRBA1, LAMA4, LAMP1, LCK, LPIN1, LRP1, LRPAP1, MAD1L1, MAG, MAGI2, MAOA, MAPK8IP3, MAPT, MBNL2, MBP, MBTPS1, MDFI, MEIS1, METTL15, MGRN1, MLC1, MLF1, MLL2, MLLT1, MRPL23, MT1E, MT1M, MT1X, MYH14, MYO1D, MYO1F, NAD+, NAV1, NDUFS7, NELF, NFIX, NHLRC1, NMNAT2, NOS2, NOS3, NPLOC4, NR2E1, NR2F1, NRTN, OBSCN, OGT, OLFM2, OLFML2B, OPRD1, OPRM1, PAH, PAX6, PBX1, PCDHA1,	271

		PCDHGA3, PDE4C, PDE6B, PDPN, PENK, PER3, PEX7, PGAM2, PGR, PHOX2B, PIK3CD, PIP5K1C, PLXNC1, POLG, PON1, PPP2R5C, PRDM16, PRKCZ, PRKX, PRRC2A, PRRT1, PTCHD1, PTGFRN, PTGIS, PTPRG, PTPRN2, RAB3A, RAB40B, RAC1, RIMS1, RNF128, RNF220, RPL39, RPS9, RUNX1, RUNX3, S100A1, S1PR4, SAT1, SETBP1, SGK1, SHROOM2, SHROOM3, SKAP1, SLC25A6, SLC2A9, SLC8A3, SLITRK1, SMARCA2, SMTN, SMU1, SPG7, SPHK2, SPI1, SPTLC1, SSBP3, SUN1, SYN1, SYP, TAF1, TAOK2, TBCD, TGFB1, THRA, TMEM47, TRAPPC9, TRIO, TSC2, TSHZ3, TSNARE1, TTLL11, TUBB1, TULP1, ULK2, UNC5C, USH1C, VAC14, VAV2, VGLL4, WNT10A, WT1, ZFP36L1, ZGPAT, ZIC2, ZMYND8, ZNF274, ZNF490, ZNF608	
development of nervous tissue	2.66E-10	ARX, BAIAP2, BARHL2, BDNF, BMP4, BMP7, CIT, CNP, CNTNAP1, CTF1, DHH, EBF3, EN1, FOXB1, GRIN1, GRM4, HOXA1, HOXA3, HOXA4, HOXB1, HOXB3, IGF1R, KCNA3, KNDC1, LHX6, MAG, MAPK8IP3, MAPT, MBP, NDEL1, NEUROD2, NFIX, NR2E1, NR2F1, NRN1, NRTN, PACSIN1, PAX6, PBX1, PHOX2B, PIP5K1C, POU4F1, PRMT1, PTPRS, RAB3A, RAC1, RUNX3, SGK1, SLITRK1, TULP1, ZIC2, ZIC3	52
proliferation of cells	3.67E-09	ABCG1, ABL1, AFAP1, AKAP12, AKT1, ARX, BCL3, BDNF, BMP4, BMP7, BOP1/LOC727967, BRCA1, BRD4, BRF1 (includes EG:2972), BSG, CALCA, CALCB, CAPN1, CARD11, CARM1, CBFA2T3, CCND1, CD1D, CD247, CD47, CD58, CNP, COL4A1, COMP, CR1L, CREBBP, CTBP1, CTBP2, CTF1, CTGF, DAXX, DEF6, DGKA, DHH, EDNRB, EPHA2, EPHB4, ETS1, F12, FASN, FGF19, FLI1, FOXA2, FOXA3, FOXD2, FOXJ1, FOXK1, FOXO3, FOXO4, FURIN, FZR1, GBX2, GFI1, GNA11, GNG7, GPC3, GPC4, GRM4, HDAC4, HHEX, HHIP, HLA-DPB1, HNF1B, HOXA1, HOXA3, HOXA5, HOXA9, HOXB1, HOXB3, HOXB7, HOXB9, HOXD13, ID4, IGF1R, IGF2BP1, IGFBP4, INSR, ITGAL, JAK3, JARID2, KCNA3, KLB, KLF13, KLF6, LAMA4, LCK, LEPREL2, LPIN1, LRP1, LRPAP1, MAD1L1, MAP3K11, MAP4K1, MAP7, MAPT, MBP, MCF2L, MEIS1, MLL2, MLLT1, MPL, MT1E, MTA1, MVD, MYH14, NAD+, NCOR2, NFATC1, NFIC, NKX2-3, NOLC1, NOS2, NOS3, NR2E1, NR5A1, OAZ1, OPRM1, PAX6, PBX1, PDPK1, PDPN, PENK, PGAM2, PGR, PHOX2B, PIK3CD, PIK3R2, PIP5K1C, PITX2, PLXNB2, PPP2R5C, PRDM16, PRKAR1B, PRKCZ, PTPRG, RAC1, RNF128, ROR2, RPS9, RUNX1, RUNX3, S100A11, S1PR4, SAT1, SEMA4C, SF1, SGK1, SH2D3C, SKAP1, SLC25A6, SLC7A5, SMAD7, SMARCA2, SOX8, SPHK2, SPI1, SPN, SSBP3, TGFB1, THRA, TRIO, TRRAP, TSC2, TXNRD1, UBE2I, UBE2J2, UHRF2, VAV2, WNT5A, WT1, ZBTB7A, ZFP36L1	177
morphogenesis of organ	8.36E-09	ABLIM1, BMP4, BMP7, COL5A1, COMP, DVL3, EPHB4, FLI1, FOXA2, FURIN, GAA, GAMT, GPC3, HHEX, HHIP, HOXA3, HOXA5, HOXC4, HOXD13, IGF1R, INSR, KLF6, MAPK8IP3, NCOR2, NKX2-3, NR2E1, PAX6, PBX1, PITX2, PRKAR1B, SHROOM2, TGFB1, THRA, TSHZ3, WNT10A, WNT5A, WT1, ZIC3	38
quantity of cells	1.38E-08	ABL1, ACTN4, AKT1, APLP2, ARX, BARHL2, BDNF, BMP4, BMP7, BRCA1, BSG, CALCA, CAPN1, CARD11, CARM1, CCND1, CD1D, CD247, CD47, CIT, CREBBP, CTF1, CTGF, DEF6, DGKZ, DMD, EDNRB, ELL, EN1, ETS1, FLI1, FLNC, FURIN, GFI1, GNG7, HELT, HOXA1, HOXA3, HOXA5, HOXA9,	95

		HOXB1, HOXB3, HOXB7, IGF1R, INSR, ITGAL, JAK3, JARID2, LCK, LFNG, LSP1, MAEA, MAP7, MAPT, MEIS1, MPL, MYBPC1, NAD+, NELF, NEUROD2, NFATC1, NKX2-3, NOS2, NOS3, NR2F1, NR5A1, NRTN, OPRM1, PAX6, PBX1, PGR, PIK3CD, PITX2, POU4F1, PRKAR1B, PRKCZ, PSMB10, RAC1, RSPH1, RUNX1, RUNX3, SGK1, SH2D3C, SHC2, SIM1 (includes EG:20464), SPI1, SPN, TGFB1, THRA, TSC2, TXNRD1, VAV2, WNT5A, WT1, ZBTB7A	
development of brain	1.44E-08	ABL1, ABR, APLP2, ARX, BDNF, BMP4, BMP7, CCND1, EBF3, EN1, EOMES, FOXA2, FOXB1, GBX2, GRIN1, HHEX, HNF1B, HOXA1, ID4, IGF1R, MAPK8IP3, NCOR2, NDEL1, NEUROD2, NOS2, NR2E1, NR2F1, NR5A1, PAX6, PDPK1, PTPRS, RAC1, SDF4, SHROOM2, SIM1 (includes EG:20464), SPHK2, TRIO, UNC5C, WNT5A, ZIC2	40
development of body axis	2.43E-08	BMP4, CSNK1D, EPHA2, FOXA2, GPC3, HHEX, HNF1B, HOXA1, HOXA3, HOXA4, HOXA5, HOXA9, HOXB1, HOXB3, HOXB7, HOXB9, HOXC4, HOXD13, HOXD4, MDFI, NFIC, PAX6, PBX1, PITX2, SSBP3, WNT5A, ZIC3	27
patterning of rostrocaudal axis	2.58E-08	FOXA2, HHEX, HNF1B, HOXA1, HOXA3, HOXA4, HOXA5, HOXA9, HOXB1, HOXB3, HOXB7, HOXB9, HOXC4, HOXD13, HOXD4, PAX6, PBX1, WNT5A, ZIC3	19
hematopoiesis	4.59E-08	ABL1, AKT1, BCL3, BMP4, BRCA1, BSG, CALCB, CARD11, CBFA2T3, CCND1, CD1D, CD247, CR1L, CREBBP, CTBP1, CTF1, DEF6, EBF1, EIF2C2, EOMES, EPHA2, ETS1, FLI1, FOXO3, GFI1, HHEX, HOXA5, HOXA9, HOXB3, HOXB7, IGF1R, ITGAL, JAK3, KLF13, LAMP1, LCK, LFNG, MAD1L1, MAEA, MAP4K1, MBP, MEIS1, MLF1, MLLT1, MPL, NFATC1, OGT, PBX1, PDPK1, PENK, PIK3CD, PIK3R2, PRDM16, PRKCZ, RAC1, RNF128, ROR2, RUNX1, RUNX3, SPI1, SPN, TGFB1, THRA, TSC2, VAV2, WT1, ZFPM1	67

Supplementary Table 12 - Differentially methylated CGIs between cerebellum and cortex are significantly associated with neurodevelopment and neurobiological function.

Functions Annotation	p-Value	Molecules
neurogenesis	3.07E-09	AKT1, APLP2, ARHGEF1, ARHGEF10, AXIN1, B4GALNT1, BAIAP2, BHLHE22, BRSK2, CACNA1A, CACNA1H, CELSR2, CSNK1D, DRGX, EBF3, FGF3, FGFR3, GET4, GFRA3, HELT, HOXB3, JAG2, KIF5C, KNDC1, MAPK8IP3, MAPT, MARK2, MEIS1, METRN, NFATC2, NFATC4, NFIX, NGFR, NOTCH1, NPTX1, NR2E1, NR2F1, NR4A2, NRTN, NTN1, NUMBL, PAK4, PALLD, PAX6, PAX7, PLXNA3, PLXNB2, POU4F1, PPP2R2C, PPP2R2D, PRDM16, PRDM8, RUNX3, SEMA4C, SERPINF2, SGK223, SYNGR1, TBR1, TIAM1, TNK2, TUBB3, VAV2, ZIC1, ZNF274, ZNF536
guidance of neurites	9.86E-08	ARHGEF1, BAIAP2, CACNA1A, CDH4, CSNK1D, DRGX, EPHB3, FGF3, FGFR3, GFRA3, KIF5C, KNDC1, MAPK8IP3, MAPT, MARK2, NFATC2, NFATC4, NFIX, NGFR, NOTCH1, NPTX1, NRTN, NTN1, PAK4, PALLD, PAX6, PLXNA1, PLXNA3, POU4F1, PPP2R2C, PPP2R2D, RGMA, RUNX3, SERPINF2, SGK223, SYNGR1, TBR1, TIAM1, TNK2, UNC5A, VAV2
formation of axons	7.83E-06	ARHGEF1, MARK2, METRN, NGFR, PAK4, PAX6, PLXNB2, TIAM1
development of brain	1.62E-05	APLP2, CACNA1A, CBS, EBF3, EOMES, FGFR3, HHEX, HSPG2, MAPK8IP3, MDGA1, MSX1, MYO16, NCOR2, NOTCH1, NR1H2, NR2E1, NR2F1, NTN1, NUMBL, PAX6, PLXNA3, PTPRS, SDF4, SHROOM2, SMARCA4, TBR1, ZIC1, ZIC2
outgrowth of axons	2.76E-05	MAPT, MARK2, NFATC2, NFATC4, NFIX, NGFR, NTN1, PAK4, PLXNA3
development of forebrain	3.79E-05	APLP2, EBF3, FGFR3, HHEX, MAPK8IP3, MSX1, NCOR2, NOTCH1, NR2E1, NR2F1, NTN1, NUMBL, PAX6, PTPRS, SMARCA4, ZIC1
quantity of neurons	4.63E-05	APLP2, CTSD, HELT, JAG2, KIF5C, MAPT, NGFR, NHLH1, NOTCH1, NR2F1, NR4A2, NRTN, NTN1, PAX6, POU4F1, PRKAR1B, RUNX3, TBR1
growth of neurites	4.70E-05	ARHGEF1, BAIAP2, CACNA1A, CSNK1D, FGF3, FGFR3, KNDC1, MAPT, MARK2, NFATC2, NFATC4, NFIX, NGFR, NOTCH1, NPTX1, NRTN, NTN1, PAK4, PALLD, PLXNA3, POU4F1, PPP2R2C, PPP2R2D, SERPINF2, SGK223, SYNGR1, TIAM1, TNK2, VAV2
outgrowth of sensory axons	5.34E-05	NFATC2, NFATC4, NTN1, PLXNA3
quantity of spinal cord cells	1.09E-04	NGFR, NRTN, PRKAR1B
formation of neurites	1.17E-04	ARHGEF1, MAPT, MARK2, METRN, NFIX, NGFR, PAK4, PAX6, PLXNB2, TIAM1, TNK2
growth of axons	1.28E-04	ARHGEF1, CACNA1A, MAPT, MARK2, NFATC2, NFATC4, NFIX, NGFR, NTN1, PAK4, PLXNA3, POU4F1
guidance of axons	1.92E-04	CDH4, DRGX, EPHB3, GFRA3, KIF5C, MAPK8IP3, NGFR, NTN1, PAX6, PLXNA3, RUNX3, TBR1, UNC5A
differentiation of	2.12E-04	AKT1, BHLHE22, BRSK2, DRGX, EBF3, FGFR3, GET4, HELT, KNDC1, MAPK8IP3, MAPT, MEIS1, METRN,

neurons		NGFR, NOTCH1, NR2E1, NR4A2, PAK4, PAX6, PLXNB2, POU4F1, TBR1, TUBB3, ZNF536
migration of neurons	2.94E-04	DRGX, EBF3, GFRA3, NFIX, NGFR, NR2F1, NR4A2, NTN1, PAK4, PAX6, PIP5K1C, PLXNB2, POU4F1, SEMA4C, TBR1, TIAM1
outgrowth of neurites	3.34E-04	BAIAP2, CSNK1D, FGF3, FGFR3, MAPT, MARK2, NFATC2, NFATC4, NFIX, NGFR, NPTX1, NRTN, NTN1, PAK4, PALLD, PLXNA3, POU4F1, PPP2R2C, SERPINF2, SGK223, SYNGR1, TIAM1, TNK2, VAV2
development of rhombencephalon	4.34E-04	CACNA1A, CBS, FGFR3, MYO16, PAX6, PTPRS, SDF4, SMARCA4, TBR1, ZIC1
auditory system development	4.52E-04	AKT1, CELSR1, FGF3, FGFR3, JAG2, KCNQ1, MSX1, NOTCH1, NTN1, PRRX2, ROR2, SHROOM2, TBX1
projection of axons	4.83E-04	NGFR, NR2F1, NTN1, TBR1
neurological process of cells	4.88E-04	APBA3, BSN, CACNA1A, CACNA1H, CCKBR, CHRNA4, CNTNAP1, DLGAP1, DNAJC5, FGFR3, GABRD, GFRA3, GNA11, GRIK5, GRM4, JPH3, KCNN3, KCNQ1, KCNQ2, NFATC4, NGFR, NOTCH1, NPTX1, NRTN, NRXN2, PAX6, PPP2R2C, PRKAR1B, PRKCZ, SLC17A7, SYNGR1, VIPR1
differentiation of Cajal-Retzius neurons	9.20E-04	PLXNB2, TBR1
formation of subventricular zone	9.20E-04	NR2E1, PAX6
generation of projection neurons	9.20E-04	NR2E1, PAX6
quantity of dorsal root ganglion cells	9.20E-04	NGFR, NRTN
neurological process of neurons	1.42E-03	APBA3, BSN, CACNA1A, CCKBR, CHRNA4, DLGAP1, DNAJC5, GABRD, GFRA3, GRIK5, GRM4, JPH3, KCNN3, KCNQ2, NFATC4, NGFR, NOTCH1, NPTX1, NRTN, NRXN2, PAX6, PRKAR1B, PRKCZ, SLC17A7, SYNGR1, VIPR1
innervation of nervous tissue	1.46E-03	GFRA3, NR2F1, NRTN, POU4F1
development of cerebellum	1.49E-03	CACNA1A, CBS, FGFR3, MYO16, PAX6, PTPRS, SDF4, ZIC1
proliferation of neuroblasts	1.99E-03	GFRA3, NOTCH1, NUMBL, PAX6, PLXNB2, SEMA4C, ZIC1
migration of granule cell precursors	2.04E-03	NFIX, PLXNB2, SEMA4C
orientation of axons	2.70E-03	NTN1, PRKCZ
projection of thalamocortical axons	2.70E-03	NR2F1, NTN1

migration of neuroblasts	2.76E-03	NFIX, NGFR, PLXNB2, SEMA4C
development of ganglion cells	2.85E-03	NGFR, NRTN, POU4F1
proliferation of neuronal cells	3.39E-03	GFRA3, MAPT, NOTCH1, NR2E1, NUMBL, PAX6, PDGFB, PLXNB2, SEMA4C, ZIC1
neurotransmission of nervous tissue	4.39E-03	APBA3, BSN, CACNA1A, CCKBR, CHRNA4, DLGAP1, DNAJC5, GABRD, GRIK5, GRM4, KCNQ2, NPTX1, NRXN2, PRKCZ, RAB3A, SLC17A7, VIPR1
innervation	4.95E-03	GFRA3, NGFR, NR2F1, NRTN, POU4F1
proliferation of cerebral cortex cells	4.99E-03	MAPT, PAX6, SLC2A8
development of trigeminal ganglion neurons	5.30E-03	NRTN, POU4F1
morphology of inner ear	5.30E-03	KCNQ1, PRRX2
neurological process of photoreceptors	5.30E-03	BSN, SLC17A7
neurological process of mice	5.65E-03	ABCA2, CREBBP, GABRB3, JPH3, KCNC1, KCNQ2, MAPT, NGFR, NOTCH1, NR4A2, NRTN, PAX6, PTPRN2, RAB3A, TBX1, TNFRSF1B
development of neurons	6.12E-03	EBF3, FGFR3, GFRA3, NGFR, NOTCH1, NR2F1, NR4A2, NRTN, PAK4, PAX6, POU4F1
development of commissure	6.34E-03	MAPK8IP3, NR2E1, NTN1
synaptic transmission of neurons	6.75E-03	APBA3, BSN, CACNA1A, CCKBR, DLGAP1, DNAJC5, GABRD, GRIK5, GRM4, KCNQ2, NPTX1, NRXN2, PRKCZ, SLC17A7, VIPR1
morphogenesis of dendrites	6.93E-03	CACNA1A, CELSR2, KNDC1, NR2E1, NUMBL
loss of neurons	7.31E-03	CTSD, MAPT, NGFR, NR1H2, NR4A2, NRTN, POU4F1, VAC14
differentiation of radial glial cells	8.66E-03	METRN, NOTCH1
exit from cell cycle progression of neurons	8.66E-03	PAX6, ZIC1
size of lens	8.66E-03	MEIS1, PAX6
development of	8.81E-03	EBF3, MAPK8IP3, NCOR2, NR2E1, NTN1, PAX6, PTPRS

telencephalon		
development of ear	8.86E-03	AKT1, CELSR1, FGF3, FGFR3, KCNQ1, PRRX2, ROR2, SHROOM2, TBX1
development of nerves	9.53E-03	ARHGEF10, B4GALNT1, DRGX, HOXB3, NGFR, NRTN
quantity of ganglion cells	9.64E-03	NGFR, NRTN, POU4F1
neurotransmission	1.03E-02	APBA3, BSN, CACNA1A, CCKBR, CHRNA4, DLGAP1, DNAJC5, GABRD, GNA11, GRIK5, GRM4, KCNQ1, KCNQ2, NPTX1, NRXN2, PRKCZ, RAB3A, SLC17A7, VIPR1
proliferation of brain cells	1.11E-02	MAPT, PAX6, PLXNB2, SEMA4C, SLC2A8, ZIC1
proliferation of granule cell precursors	1.16E-02	PLXNB2, SEMA4C, ZIC1
motor function	1.18E-02	DLL4, GABRB3, JPH3, KCNC1, KCNC3, MAPT, NR4A2, NRTN
quantity of sensory neurons	1.23E-02	JAG2, NGFR, NOTCH1, RUNX3
differentiation of amacrine cells	1.27E-02	EBF3, NR4A2
differentiation of glial progenitor cells	1.27E-02	NOTCH1, PLXNB2
proliferation of hippocampal cells	1.27E-02	MAPT, SLC2A8
quantity of oligodendrocytes	1.38E-02	FGFR3, NOTCH1, PAX6
synaptic transmission	1.54E-02	APBA3, BSN, CACNA1A, CCKBR, CHRNA4, DLGAP1, DNAJC5, GABRD, GRIK5, GRM4, KCNQ2, NPTX1, NRXN2, PRKCZ, RAB3A, SLC17A7, VIPR1
axonogenesis of organism	1.62E-02	ARHGEF1, CACNA1A, NGFR
chemorepulsion of axons	1.75E-02	NTN1, UNC5A
development of dopaminergic neurons	1.75E-02	FGFR3, NR4A2
innervation of axons	1.75E-02	NR2F1, POU4F1
development of inner ear	2.19E-02	CELSR1, FGF3, FGFR3, KCNQ1, PRRX2, ROR2, TBX1
differentiation of	2.20E-02	FGFR3, METRN, NOTCH1, NR2E1, PAX6

astrocytes		
transport of synaptic vesicles	2.20E-02	LPHN1, PIP5K1C, RAB3A, SCRIB, SPTBN2
differentiation of neurosphere cells	2.28E-02	METRN, NGFR
firing of dopaminergic neurons	2.28E-02	CHRNA4, KCNN3
branching of neurites	2.34E-02	BSN, EPHB3, FNBP1, KNDC1, NGFR, NOTCH1, NTN1, NUMBL, PALM, ROR2
morphogenesis of middle ear	2.46E-02	MSX1, PRRX2, TBX1
development of hippocampus	2.72E-02	CACNA1A, NR2E1, NTN1, PLXNA3, PTPRS
survival of ganglion cells	2.79E-02	EHD4, NRTN, POU4F1
function of nervous system	2.82E-02	DLL4, GABRB3, JPH3, KCNC1, KCNC3, MAPT, NHLH1, NR4A2, NRTN
development of anterior commissure	2.88E-02	NR2E1, NTN1
development of cerebellar cortex	2.88E-02	CACNA1A, ZIC1
innervation of neurons	2.88E-02	GFRA3, NRTN
patterning of telencephalon	2.88E-02	NR2E1, PAX6
quantity of amacrine cells	2.88E-02	NR4A2, PAX6
regulation of neurons	2.88E-02	NGFR, NRTN
S phase of neuronal progenitor cells	3.04E-02	PAX6
branching of trigeminal ganglion neurons	3.04E-02	POU4F1

Supplementary Table 13 – Genes in the blue intragenic CGI methylation module are significantly enriched for in relevant gene expression modules from published datasets.

Dataset Reference	Module	Enrichment P-value
Cahoy et al (2008)	Neuron	0.00006
Miller et al (2010)	Astrocytes (brown)	0.00016
Miller et al (2010)	Neuron (tan)	0.00112
Oldham et al (2008)	Cortex (salmon)	0.00135
Lein et al (2007)	Neuron	0.00249
Miller et al (2010)	Microglia (magenta)	0.00328
Winden et al (2009)	Telencephalic Interneurons (light yellow)	0.00361
Oldham et al (2008)	Astrocytes (brown)	0.00756
Oldham et al (2006)	Cortex (brown)	0.00853
Winden et al (2009)	Cingulate Interneurons (orange)	0.00951

Cahoy JD, et al. A transcriptome database for astrocytes, neurons, and oligodendrocytes: a new resource for understanding brain development and function. J Neurosci. 2008 Jan 2;28(1):264-78.

Lein ES, et al. Genome-wide atlas of gene expression in the adult mouse brain. Nature. 2007 Jan 11;445(7124):168-76.

Miller JA, Horvath S, Geschwind DH. Divergence of human and mouse brain transcriptome highlights Alzheimer disease pathways. Proc Natl Acad Sci U S A. 2010 Jul 13;107(28):12698-703

Oldham MC, Konopka G, Iwamoto K, Langfelder P, Kato T, Horvath S, Geschwind DH. Functional organization of the transcriptome in human brain. Nat Neurosci. 2008 Nov;11(11):1271-82.

Winden KD, Oldham MC, Mirnics K, Ebert PJ, Swan CH, Levitt P, Rubenstein JL, Horvath S, Geschwind DH. The organization of the transcriptional network in specific neuronal classes. Mol Syst Biol. 2009;5:291

Supplementary Table 14 – The top 50 CGI shore DMRs across blood, cerebellum and cortex. Values correspond to normalized MeDIP-seq read-counts across each feature location. Data for all annotated features is available for download from http://epigenetics.iop.kcl.ac.uk/brain.

Location	Gene	Blood	Cerebellum	Cortical	Mean	CV
7:154861384-154863384		1.12	189.46	1.02	63.87	1.68
7:154860615-154862615		1.15	136.11	0.98	46.08	1.66
14:56351720-56353720		0.37	98.30	1.30	33.32	1.64
5:58368593-58370593	PDE4D	1.14	163.28	4.91	56.44	1.61
5:146235539-146237539	PPP2R2B	1.51	91.93	1.07	31.50	1.61
1:67924858-67926858	GADD45A	3.35	160.01	3.98	55.78	1.59
14:56348869-56350869		3.41	133.97	2.97	46.79	1.58
5:58371638-58373638	PDE4D	2.25	114.21	2.96	39.81	1.58
2:56266043-56268043	CCDC85A	5.69	259.55	9.40	91.55	1.57
17:44054082-44056082	HOXB9	109.04	2.16	3.58	38.26	1.56
8:26428747-26430747		1.91	81.94	2.27	28.71	1.55
14:56348040-56350040		3.78	112.43	2.75	39.66	1.55
1:82041636-82043636		1.51	90.19	3.41	31.70	1.55
5:59225651-59227651	PDE4D	1.91	105.72	4.83	37.49	1.54
17:44055279-44057279	HOXB9	75.05	1.96	2.39	26.47	1.53
13:66703460-66705460	PCDH9	3.81	111.66	3.76	39.74	1.53
15:50881306-50883306		3.01	124.55	5.86	44.48	1.53
20:34922415-34924415	C20orf117	50.05	0.73	1.94	17.57	1.51
2:183831588-183833588		0.75	156.69	11.98	56.47	1.51
3:124646905-124648905	ADCY5	6.74	117.26	2.50	42.17	1.51
17:44930315-44932315	NGFR	3.78	165.62	10.08	59.83	1.51
5:73973307-73975307		2.66	106.51	5.57	38.25	1.51
1:41605129-41607129	AC093151.2	3.01	111.05	5.76	39.94	1.50
2:144998739-145000739		2.63	129.90	8.18	46.90	1.50
4:96690017-96692017	UNC5C	0.75	74.92	4.93	26.87	1.50
14:69304421-69306421	SFRS5	119.78	2.66	7.69	43.38	1.49
7:69798623-69800623	AUTS2	7.21	340.01	26.51	124.58	1.49

2:172805728-172807728		3.38	276.03	24.05	101.15	1.49
1:210796291-210798291		69.71	1.97	3.45	25.04	1.49
9:89303637-89305637	DAPK1	3.81	162.22	11.43	59.16	1.49
15:87744857-87746857		3.77	67.15	1.49	24.14	1.48
7:69699392-69701392	AUTS2	5.62	392.19	35.12	144.31	1.48
15:50861479-50863479	ONECUT1	5.31	165.39	10.62	60.44	1.48
4:82609634-82611634	RASGEF1B	1.52	56.95	2.79	20.42	1.48
5:77977624-77979624		4.46	195.06	15.14	71.55	1.48
1:199886411-199888411	NAV1	6.80	127.82	5.31	46.64	1.48
7:27120172-27122172	AC010990.3	54.31	1.36	2.72	19.46	1.48
7:6408567-6410567	RAC1	188.31	9.01	10.28	69.20	1.47
12:118724304-118726304	CIT	97.18	2.63	6.49	35.43	1.47
3:185215681-185217681	ABCC5	47.84	0.99	2.56	17.13	1.47
22:49516885-49518885	SHANK3	44.38	1.02	2.13	15.84	1.47
10:98793424-98795424	SLIT1	4.15	105.93	6.03	38.70	1.47
7:27121951-27123951	AC010990.3	53.17	1.80	2.39	19.12	1.47
17:44026531-44028531	HOXB6	50.91	0.83	3.20	18.32	1.46
1:20382947-20384947		11.70	287.10	19.74	106.18	1.46
11:33844933-33846933	LMO2	3.38	110.34	7.59	40.44	1.46
1:85815523-85817523	CYR61	3.74	87.04	4.52	31.76	1.46
17:44026457-44028457	HOXB6	50.14	0.83	3.20	18.06	1.46
6:32227807-32229807	PRRT1	58.74	2.20	2.83	21.26	1.46
2:62277962-62279962		9.07	198.25	12.48	73.27	1.46

Supplementary Table 15 - Cross-tissue variable CGI shores show a strong enrichment for functions involved in tissue development and nervous system function. Shown are the top 25 biological functions identified by IPA analysis on the most variable CGI shores.

Functional Annotation	p-Value	Molecules	Number
genetic disorder	7.06E-25	ABCB10, ABCC5, ABCG1, ABL1, ACACA, ACADM, ACADVL, ACTL8, ACYP2, ADAM17, ADAR, ADARB2, ADCK4, ADCY3, ADCY5, ADCY9, ADD1, ADRA2C, ADRBK2, AHR, AIM1, AK8, AKAP10, AKAP12, AKAP13, AKAP8, AKAP8L, ALDOA, ALG1, ALOX5, ALX4, AMDHD1, ANKFY1, ANKRD11, ANKRD52, ANKS1B, AP1S1, AP2A2, AP3B2, APBA2, APBB2, APLP1, APLP2, AQP3, ARFGAP3, ARHGAP20, ARHGEF10, ARHGEF6, ARHGEF7, ARNT, ASAP3, ASB3/GPR75-ASB3, ASCL1, ASPH, ASPM, ASTN2, ASXL1, ATAD2, ATIC, ATP2A1, ATP2A3, ATP2B2, ATP5A1, ATP5H, ATP5O, ATP6V1E1, ATP9A, ATR, ATRN, ATRNL1, ATXN2L, ATXN3, ATXN7, AUH, AUTS2, AXIN2, B3GALTL, BAALC, BAD, BAIAP2, BANP, BBS10, BCAN, BCL10, BCL2L1, BCR, BCS1L, BDNF, BEX4, BMP4, BRD1, BRSK1, BRSK2, BSCL2, BSG, C10orf72, C12orf41, C12orf57, C13orf15, C17orf57, C17orf76, C18orf8, C1orf135, C1QBP, C21orf91, C4orf22, C6orf125, C6orf170, C9orf86, CA11, CA8, CACNA1B, CACNA1C, CACNA1G, CACNA1I, CACNA2D3, CACNG3, CACNG4, CADPS, CALML5, CAMK2B, CAMK2N1, CAMTA1, CAPN1, CAPNS1, CARTPT, CASP9, CAST, CCDC3, CCDC33, CCDC85A, CCDC86, CCK, CCNB1, CCT4, CD44, CD81, CD8A, CD9, CDC123, CDC14A, CDC20, CDK5, CDK5R1, CDK9, CDYL, CELSR2, CERKL, CETN2, CHCHD6, CHFR, CHKA, CHRNA4, CHRNB4, CHST11, CHUK, CIITA, CIRBP, CIT, CKS1B, CLCN2, CLCN7, CLDN1, CLN5, CLSTN1, CLSTN2, CLU, CNNM2, CNNM4, CNP, CNTNAP1, CNTNAP5, COG1, COIL, COL5A1, COL7A1, COPG, COX5B, CPEB3, CPNE5, CPT1C, CRAMP1L, CREB3L2, CREBBP, CRELD1, CRHR2, CRIM1, CRK, CRLF1, CSDA, CSMD2, CSRP1, CSTB, CTBP2, CTDP1, CTNNA2, CTNNAL1, CTNND2, CTR9, CTTNBP2, CUL3, CUX1, CXCL3, CXXC5, CYB5B, CYC1, CYP46A1, CYP4V2, CYP51A1, CYR61, CYTL1, DAB2IP, DAPK1, DBNL, DBP, DCLK1, DCTN4, DCTN5, DCUN1D2, DDHD2, DDX56, DEAF1, DEF8, DGCR2, DHCR7, DHPS, DHX40, DIABLO, DIP2C, DKK2, DLEC1, DLG4, DLX4, DNAJA1, DNAJB1, DNAJB12, DNAJB2, DNER, DNM1L, DNMT3A, DOCK1, DOCK2, DOCK9, DOLK, DOPEY2, DPAGT1, DSCAM, DSCAML1, DSP, DTD1, DUSP1, DUSP3, DUSP6, DYNC1H1, DYX1C1, EBF1, EBF3, ECE2, ECHDC1, EDIL3, EDNRA, EEF1A2, EEF1G, EEF2, EEPD1, EFNB2, EGR1, EHBP1, EHD2, EIF2AK1, EIF4A1, EIF4A3, EIF5, EIF5A, ELL2, EML1, EMX2, ENC1, ENO2, ENSA, ENTPD3, ENTPD4, EPAS1, EPB41L3, EPDR1, EPHA4, EPHA7, EPHB2, ERCC1, ERCC2, ERI1, ESF1, ESRRG, EXD1, EXOSC3, FADD, FAIM2, FAM125B, FAM129A, FAM13C, FAM172A, FAM19A5, FAM20C, FAM69B, FAM78B, FAN1, FBL, FBN2, FDFT1, FEM1A, FGF14, FGF19, FGFR2, FHOD3, FKBP4, FKRP, FLCN, FLI1, FNDC1, FNTA, FOXE1, FOXF2, FOXK1, FOXN3, FOXO3, FOXP1, FRMPD4, FRZB, FSTL1, FTL, FUBP1, FXYD1, FZD4, FZR1, G0S2, GAB1, GAD1, GAD2, GADD45A, GALNS, GALNT10, GALNT2, GALNTL4, GALT, GAN, GAS1, GATA6, GBAS, GDF5, GDF6, GDNF, GFRA2, GGA1, GGA2, GGA3, GGCX, GGNBP2, GIPR, GLE1, GLI2, GLIPR2, GLIS2, GLP1R, GMNN, GNA11, GNAL, GNB2L1, GNG3, GNG7, GNPTG,	1070

		GOT1, GPC1, GPC6, GPD1, GPM6B, GPR123, GPR6, GPT2, GPX4, GRM2, GRSF1, GSTA4, GSTT1, GTF3C4, GYS1, HAGH, HAX1, HBA1/HBA2, HCN4, HDAC11, HDAC3, HECTD3, HERC2, HEY2, HGS, HIST1H2AB/HIST1H2AE, HIST1H3A (includes others), HIST1H4A (includes others), HMGN1, HMGXB4, HN1L, HOXA1, HOXA10, HOXA3, HOXA5, HOXA9, HOXB3, HOXC4, HOXC5, HOXC8, HPS4, HPSE2, HS6ST3, HSF1, HSPA12A, HSPA6, HSPA8, HTATIP2, HYAL2, ICAM5, ID1, ID4, IDE, IFITM1, IGF1R, IGFBP1, IGFBPL1, IHH, IKBKG, IL12A, IL17RB, IMPDH1, IMPDH2, INHBB, INPP5A, INSIG1, INSR, INTS3, IQGAP1, IRF8, ITGA1, ITGA5, ITGAL, ITM2B, ITPKB, JAG1, JAG2, JAK3, JAKMIP1, JAM3, JARID2, JPH3, JPH4, JUND, KALRN, KCNA3, KCNAB1, KCNB1, KCNC3, KCND2, KCNG2, KCNH2, KCNIP1, KCNK15, KCNMA1, KCNQ2, KCNQ4, KCTD13, KCTD8, KDELR2, KDM2B, KDM4B, KEAP1, KIAA0146, KIAA0368, KIAA1279, KIAA1467, KIF1B, KLF10, KLF11, KLF16, KLF6, KPNA6, KREMEN1, KRT86, KY, LAMB3, LAMP1, LASP1, LBR, LETM1, LGR4, LHFPL3, LIG4, LIMCH1, LIN7B, LIPE, LMF1, LMNB2, LMO4, LMTK2, LMX1B, LONP2, LPIN1, LRCH1, LRFN2, LRP11, LRPAP1, LRRC7, LRRK1, LRRK2, LSM14A, LTBP2, LYN, MAF, MAGI2, MAL2, MAMDC2, MANF, MAP1B, MAP3K11, MAP3K14, MAP4K3, MAP4K4, MAP6, MAPK8IP1, MAPK8IP3, MAPKAPK2, MAST4, MCF2L, MCF2L2, MCM7, MDC1, MDGA1, MED16, MED24, MED27, MEIS1, MEIS2, MFI2, MFSD5, MGAT4B, MGRN1, MICALL1, MKNK2, MLF1IP, MLF2, MLLT1, MMD, MMP16, MMP2, MMP28, MNT, MON2, MPHOSPH6, MRAP, MRPS30, MSH6, MSRA, MSRB2, MT1X, MTA1, MTAP, MTHFD1L, MTHFSD, MTMR2, MTMR7, MTMR9, MTRR, MTUS2, MUM1, MXD1, MYCN, MYH6, MYLK2, MYO1F, MYO5A, MYO5B, NAGK, NAPA, NAV1, NBEA, NCOR2, NCS1, NDUFA13, NDUFA7, NDUFAB1, NDUFB2, NDUFB8, NDUFC1, NDUFS7, NEBL, NEDD4L, NEK4, NFIB, NFIC, NFIX, NGFR, NGFRAP1, NGRN, NHEDC2, NIPA1, NKAIN3, NKX2-8, NLGN2, NOP14, NOS2, NPHS2, NPLOC4, NPM1, NPR2, NPY, NR1D2, NR2E1, NR4A2, NR4A3, NRG1 (includes EG:112400), NSD1, NSF, NSMCE1, NTN1, NUMBL, NUP210, NXPH1, OAS3, OCIAD1, ODZ3, ODZ4, OLFM1, ONECUT2, OPA1, OPA3, OPRD1, OSBPL10, OTOA, OTUB1, OTX2, P2RX4, PACRG, PAH, PALLD, PAN3, PAPPA, PAWR, PAX5, PAX6, PAX7, PCBD1, PCDH9, PCDHB7, PCGF3, PCMTD2, PCNT, PCP2, PDCD6, PDE10A, PDE1C, PDE2A, PDE4D, PDE4DIP, PDE8A, PDGFC, PDGFRL, PDHX, PDIK1L, PDLIM4, PDPK1, PDXK, PEBP1, PELI2, PENK, PEX26, PEX5, PFKFB2, PFKP, PFN2, PGAM2, PGK1, PGM1, PGM3, PHB2, PHF21B, PHYH, PICK1, PIGU, PIK3R1, PIP5K1C, PITPNA, PKD1, PLAUR, PLCD1, PLEC, PLEKHA8, PLEKHB1, PLIN1, PLK2, PLOD2, PLXDC1, PLXDC2, PLXNC1, PMEPA1, PMP22, PMPCA, PMVK, PNPLA7, POC1B, POLG, POLR2A, POLR3A, POLRMT, POMP, PON1, PORCN, POU3F4, PPAP2A, PPM1H, PPP1R14C, PPP2R2B, PPP2R5C, PPP3CB, PRDM2, PRDX2, PRDX6, PRKACA, PRKAR2B, PRKCSH, PRKCZ, PRPF3, PRPF8, PRPSAP1, PRRC2A, PRRC2B, PRRT1, PSAP, PSMA7, PSMB10, PSMB8, PSMC6, PSMD1, PSMD13, PSMD14, PTCH1, PTCHD1, PTCHD2, PTEN, PTGER2, PTHLH, PTOV1, PTPLB, PTPN11, PTPN21, PTPRG, PTPRM, PTPRN2, PTPRU, PURA, PXDNL, PYGB, PYGM, RAB11FIP4, RAB12, RAB22A, RAB28, RAB3A, RAB40B, RAC1, RAE1, RAI1, RAMP1, RANBP1, RANBP3, RAP1GAP, RAPGEF6, RARRES1, RASGEF1B, RASGRP1, RB1, RBL2, RBM10, RBM15, RBM8A, RBMS1, RBP1, RECK, RER1, RERE, RETSAT, RFX2, RGL1, RGS12, RGS20, RGS3, RHCG, RIC8B, RIMS2, RIOK3,	

		RNF123, RNF220, ROM1, RORA, RPL13, RPL3, RPRD2, RPS11, RPS19BP1, RPS6KA2, RPS6KA3, RPS6KA5, RPS6KB1, RRP1B, RSF1, RTN3, RUNX1, RUSC1, RXRA, S100A1, SACM1L, SALL4, SARDH, SBF2, SCAF1, SCAF8, SCAPER, SCNN1G, SCOC, SCUBE2, SDF4, SDK1, SEC16A, SEC61B, SEC63, SEMA3C, SEPHS1, SERF1A/SERF1B, SESN3, SF1, SFSWAP, SGCB, SGIP1, SH3GL1, SH3KBP1, SHANK2, SHANK3, SHB, SHC4, SHE, SIM2, SIRPA, SIX3, SKAP1, SKP2, SLC11A2, SLC12A8, SLC15A4, SLC17A7, SLC22A5, SLC24A2, SLC25A1, SLC25A16, SLC25A24, SLC25A6, SLC26A4, SLC27A1, SLC2A13, SLC35B3, SLC35C1, SLC35F1, SLC37A1, SLC37A4, SLC38A10, SLC39A3, SLC41A1, SLC46A1, SLC5A1, SLC5A5, SLC9A1, SLC9A3R1, SLCO2A1, SLMAP, SMAD6, SMARCA2, SMARCA4, SMC4, SMEK2, SMOC1, SMU1, SMURF1, SMURF2, SNAI2, SND1, SNRNP25, SNTB1, SNTG2, SNX27, SNX30, SOD1, SORBS2, SORCS2, SORT1, SOST, SOX1, SOX9, SP8, SPAG16, SPAST, SPATA2, SPEG, SPG7, SPOCK3, SPTBN1, SREBF1, SRF, SRGAP3, SRPK2, SRSF9, SSBP3, ST6GALNAC5, ST6GALNAC6, ST8SIA3, STAM, STK11, STK25, STK39, STMN3, STOX1, STRN4, STX2, STXBP1, SUB1, SUCLA2, SUMF1, SUMO3, SUN1, SUOX, SYN1, SYNGR1, SYNJ2, SYT9, SYVN1, TACC1, TAF1A, TAF1C, TAOK2, TARDBP, TBC1D1, TBC1D17, TBC1D22A, TBCD, TBX4, TCF7, TCOF1, TCTA, TGFBR1, TGFBRAP1, THBD, THOC1, THOP1, THRA, THY1, TJP1, TK2, TLL2, TMC8, TMCO3, TMED4, TMEFF2, TMEM104, TMEM120B, TMEM165, TMEM87B, TNFRSF11A, TNFRSF11B, TNFRSF6B, TNFSF13, TNIK, TNNI3K, TNRC6C, TOMM20, TOPORS, TPCN2, TPD52L2, TRAP1, TRAPPC9, TRIM44, TRIM9, TRIO, TRPM3, TSC2, TSHZ3, TSSC1, TTC7B, TTC8, TUBA1A, TUBB1, TUBB2B, TUBB4, TULP3, TWF1, TWSG1, UBAC1, UBB, UBE2G2, UBIAD1, UNC5B, UNC5C, UPF1, UPF3B, UQCRC2, UQCRFS1, USP2, USP36, USP42, USP7, VAMP2, VAT1L, VAV3, VDAC2, VGLL4, VHL, VIM, VSTM2L, VSX1, WAC, WARS2, WDR19, WDR37, WDR41, WNK4, WNT10A, WNT5A, WNT5B, WNT7B, WWOX, XKR6, XPO5, YWHAG, YWHAQ, ZBTB16, ZBTB4, ZBTB43, ZBTB46, ZC3H7B, ZCCHC2, ZCCHC24, ZDHHC17, ZEB2, ZFYVE28, ZGPAT, ZIC3, ZKSCAN2, ZNF136, ZNF195, ZNF225, ZNF230, ZNF274, ZNF284, ZNF33A, ZNF398, ZNF423, ZNF43, ZNF440/ZNF808, ZNF444, ZNF532, ZNF614, ZNF615, ZNF649, ZNF747, ZNF767, ZNF829, ZNRF3	
neurogenesis	8.59E-20	ABL1, ADAM17, ADCYAP1, ADRA2C, AFG3L2, AHR, APBA2, APLP1, APLP2, ARHGEF10, ARHGEF7, ASCL1, ASPM, ATF5, ATOH1, ATP2B2, BAIAP2, BCAN, BCL2L1, BCR, BDNF, BEX2, BHLHE22, BMI1, BMP4, BRSK1, BRSK2, CABLES1, CASP9, CD44, CD9, CDK5, CDK5R1, CELSR2, CIT, CLU, CNP, CRK, CRLF1, CSNK1D, CTNNA2, CTNND2, CUX1, DAGLB, DAPK3, DCLK1, DNER, DOCK1, DSCAM, DUSP1, DVL1, EBF1, EBF3, EFNB2, ELAVL3, EMX1, EMX2, EN1, EPHA4, EPHA7, EPHB2, EPN1, ERCC2, FGF5, FGF8, FGFR2, FKBP4, FZR1, GAB1, GALNS, GDF5, GDF6, GDF7, GDNF, GET4, GFRA2, GLI2, GMNN, GNAQ, GPR12, GPR6, HBA1/HBA2, HEY2, HOXA1, HOXA2, HOXA3, HOXB3, HOXC8, HSF1, ICAM5, ID1, ID2, ID4, IGF1R, IHH, IQGAP1, ITGA1, JAG1, JAG2, KALRN, KCNA3, KCNMA1, KIAA1279, LBX1, LIG4, LMO4, LMX1A, LMX1B, LRRK2, LZTS1, MAP1B, MAPK8IP3, MATK, MDK, MEIS1, METRN, MMP2, MRAP, MYCN, MYO5A, NAPA, NDEL1, NFIX, NGFR, NGFRAP1, NOS2, NR2E1, NR4A2, NRG1 (includes	192

		EG:112400), NTN1, NTN3, NUMBL, OLIG1, OTX2, PACSIN1, PALLD, PAX5, PAX6, PAX7, PFN2, PIP5K1A, PLXNA3, PMP22, POU3F4, PPP2R2D, PRDM8, PRKACA, PSAP, PTEN, PTPN11, PTPRM, RAC1, RALGDS, RAP1GAP, RB1, RBPJ, RUSC1, RXRA, SEMA4C, SHC3, SIRT1, SIX3, SLC11A2, SLC9A1, SLIT1, SOX1, SOX9, SPR, SRF, STAM, SYN1, SYNGR1, TACC1, TARDBP, TESK1, TGFBR1, THRA, TLX1, TNK2, TRIO, TSC2, TUBA1A, ULK1, VAV3, VHL, VIM, VSX1, WNT5A, WNT7B, YWHAG, ZIC3, ZNF274	
transcription	1.92E-18	ABCG1, ABL1, ACVR1B, ADCYAP1, AES, AHR, AKAP12, AKAP13, ALX4, AP2A2, APBB2, ARHGEF7, ARNT, ASCL1, ASXL1, ATF5, ATOH1, ATXN3, ATXN7, BAD, BANP, BCL10, BCL2L1, BCR, BDNF, BEX2, BHLHA15, BHLHE22, BLOC1S2, BMI1, BMP4, BRF1 (includes EG:2972), BSX, CAMTA2, CARM1, CBX2, CBX7, CCNB1, CCNH, CD44, CD9, CDCA7L, CDK9, CDX2, CHD4, CHUK, CIITA, CLU, CREB3L2, CREBBP, CRK, CRLF3, CRTC1, CRY1, CSDA, CSRNP1, CTBP1, CTBP2, CTDSP2, CUL1, CUX1, CXCL3, CXXC1, CYTL1, DAB2IP, DBP, DCLK1, DDB1, DDX17, DEAF1, DLX4, DLX6, DNAJB5, DNMT3A, DNTTIP1, DTX3, DUSP1, DUSP3, DVL1, DVL2, E4F1, EAPP, EBF1, EBF3, EGR1, ELL, ELP2, ELP4, EN1, EPAS1, ERCC2, ERF, ESRRG, FADD, FGFR2, FLI1, FOXD4, FOXE1, FOXF2, FOXK1, FOXK2, FOXL1, FOXN3, FOXO3, FOXP1, FUBP1, FXYD1, FZD1, FZD4, FZD5, GADD45A, GATA6, GDF6, GDF7, GDNF, GLI2, GLIS2, GLP1R, GMNN, GNAQ, GNB2L1, GRHL3, GTF2H2/LOC100510744, GZF1, H1F0, HCFC2, HDAC3, HES6, HEY2, HGS, HINFP, HLF, HLTF, HMGN1, HOXA1, HOXA10, HOXA2, HOXA5, HOXA7, HOXA9, HOXB4, HOXB7, HOXB9, HOXC5, HOXC8, HSF1, HSPA8, HTATIP2, HYAL2, ID1, ID2, ID4, IHH, IKBKG, IL12A, ING2, ING5, INHBB, INSM2, INSR, IQGAP1, IRF8, ITGA5, JAG1, JAG2, JAK3, JARID2, JUND, KAT2A, KAT5, KCTD13, KDM5B, KDM6B, KEAP1, KLF10, KLF11, KLF16, KLF6, LBX1, LHX5, LMO2, LMO4, LMX1A, LMX1B, LPIN1, LYN, LZTS1, MAF, MAGI2, MAP2K7, MAP3K10, MAP3K11, MAP3K14, MAP4K4, MAPK13, MAPK8IP1, MAPKAPK2, MBD3 (includes EG:17192), MCM7, MED16, MED24, MED26, MED27, MED28, MEIS1, MEIS2, MLLT1, MNT, MSRB2, MTA1, MXD1, MYCN, MYST1, MYST2, MZF1, NCOR2, NDUFA13, NFATC1, NFIB, NFIC, NFIX, NKX2-8, NOC2L, NPM1, NPM3, NR1D2, NR1H2, NR2E1, NR2F2, NR4A2, NR4A3, NRG1 (includes EG:112400), NSD1, NTN3, NUMBL, OLIG1, ONECUT1, ONECUT2, OTUB1, OTX2, PAWR, PAX5, PAX6, PAX7, PCBD1, PCGF6, PDPK1, PEBP1, PHB2, PIAS4, PIK3R1, PKD1, PLK2, POLR1E, POLR2A, POLR2H, POLR3C, POLRMT, POU3F4, PPAP2B, PPIE, PPP4C, PRDM2, PREB, PRKACA, PRKAR2B, PRKCZ, PRKD1, PRMT2, PRMT5, PRPF6, PRPF8, PSMB10, PSMD14, PTBP1, PTEN, PTGER2, PTHLH, PTPN11, PTPN22, PTPRU, PURA, RAC1, RAI1, RASD1, RASGRP1, RB1, RBL2, RBM10, RBM14, RBM15, RBPJ, RBX1, REEP5, RFC1, RGS12, RGS20, RORA, RPS6KA3, RPS6KA5, RSF1, RUNX1, RXRA, S100A1, SALL4, SCAF1, SCAF8, SETD8, SIM2, SIRPA, SIRT1, SIX3, SKAP1, SKI, SLU7, SMAD6, SMAD7, SMARCA2, SMARCA4, SMARCC1, SMURF1, SMURF2, SNAI2, SNAI3, SNAPC2, SND1, SNIP1, SOST, SOX12, SOX7, SOX9, SRCAP, SREBF1, SRF, SRSF6, SS18L1, SSBP3, ST3GAL3, STAP2, STRAP, SUB1, SUMO1, SUMO3, TAF1A, TAF1C, TAF4, TARDBP, TBKBP1, TBX4, TCF25, TCF7, TCOF1, TEF, TGFBR1, TGFBRAP1, THAP1, THOC1, THRA,	400

		TIAL1, TICAM2, TJP1, TLX1, TNFSF13, TOLLIP, TOM1L1, TOPORS, TRIM13, TRIM17, TRRAP, TSC2, TSHZ3, USF2, USP16, USP2, USP22, VAV3, VHL, WHSC1, WNT5A, WWOX, YWHAQ, ZBTB16, ZBTB4, ZBTB7A, ZFP36L2, ZGPAT, ZIC3, ZNF136, ZNF238, ZNF256, ZNF263, ZNF274, ZNF354C, ZNF398, ZNF417/ZNF587, ZNF423, ZNF440/ZNF808, ZNF444, ZNF628, ZXDA	
neurological disorder	6.80E-18	ABCB10, ABCC5, ABL1, ACADM, ACTL8, ACYP2, ADAM17, ADARB2, ADCK4, ADCY5, ADCY9, ADCYAP1, ADRA2C, ADRBK2, AHR, AIM1, AK8, AKAP10, AKAP12, AKAP13, AKAP8L, ALOX5, AMDHD1, ANKRD11, AP1S1, AP3B2, APBA2, APBB2, APLP1, APLP2, ARHGEF10, ARHGEF6, ARHGEF7, ARNT, ASB3/GPR75-ASB3, ASCL1, ASPH, ASPM, ASTN2, ATAD2, ATOH1, ATP2A1, ATP2A3, ATP2B2, ATP5A1, ATP5H, ATP5O, ATP6V1E1, ATRNL1, ATXN2L, ATXN3, ATXN7, AUH, AUTS2, B3GALTL, BAALC, BAD, BAG5, BAIAP2, BANP, BBS10, BCAN, BCL2L1, BCR, BCS1L, BDNF, BEX4, BMI1, BRD1, BRSK1, BSCL2, C12orf41, C17orf57, C17orf76, C21orf91, C4orf22, CA11, CA8, CACNA1B, CACNA1C, CACNA1G, CACNA2D3, CACNG4, CADPS, CAMK2B, CAMTA1, CAPN1, CAPNS1, CARTPT, CASP9, CAST, CCDC3, CCDC85A, CCDC86, CCK, CCNB1, CD2BP2, CD44, CD9, CDC123, CDCA7L, CDK5, CDK5R1, CETN2, CHCHD6, CHRNA4, CHRNB4, CHST11, CIITA, CIRBP, CIT, CLCN2, CLCN7, CLK2, CLN5, CLSTN2, CLU, CNP, CNTNAP1, CNTNAP5, COL7A1, COPG, COX5B, CPNE5, CRAMP1L, CREB3L2, CREBBP, CRIM1, CRLF1, CSDA, CSMD2, CSRP1, CSTB, CTBP2, CTDP1, CTNNA2, CTNND2, CTR9, CTTNBP2, CUL3, CXCL3, CYB5B, CYC1, CYP46A1, CYP4V2, CYP51A1, DAPK1, DBNL, DBP, DCLK1, DCTN5, DCUN1D2, DEAF1, DEF8, DGCR2, DHCR7, DHPS, DIABLO, DIP2C, DLG4, DNAJA1, DNAJB1, DNAJB12, DNAJB2, DNAJC5, DNER, DNM1L, DOCK1, DOCK2, DSCAM, DSCAML1, DSP, DTD1, DUSP1, DUSP6, DYNC1H1, DYX1C1, EBF1, EBF3, ECE2, ECHDC1, EDNRA, EEF1A2, EEF1G, EEF2, EGR1, EHD2, EML1, EMX2, ENC1, ENO2, ENSA, EOMES, EPAS1, EPDR1, EPHA4, EPHB2, ERCC1, ERCC2, ESRRG, EXD1, FADD, FAM172A, FAM19A5, FAN1, FBL, FDFT1, FEM1A, FGF14, FGF8, FHOD3, FKBP4, FKRP, FNTA, FOXF2, FOXK1, FOXN3, FOXO3, FOXP1, FRMPD4, FRZB, FTL, FXYD1, FZD4, FZR1, GAB1, GAD1, GAD2, GADD45A, GALNT2, GALNTL4, GAN, GAS1, GDNF, GFRA2, GGA1, GGA2, GGA3, GLE1, GLI2, GLP1R, GNA11, GNAL, GNAQ, GNB2L1, GNG3, GNG7, GOT1, GPC1, GPD1, GPR6, GPX4, GRM2, HGS, HIST1H2AB/HIST1H2AE, HIST1H3A (includes others), HIST1H4A (includes others), HMGN1, HN1L, HOXA1, HOXA3, HOXA9, HPSE2, HS6ST3, HSF1, HSPA12A, HSPA6, HSPA8, ICAM5, ID2, ID4, IDE, IGF1R, IGFBP1, IHH, IKBKG, IL12A, IMPDH1, IMPDH2, INPP5A, INSR, INTS3, IQGAP1, IRF8, ITGAL, ITM2B, ITPKB, JAG1, JAM3, JARID2, JPH3, KALRN, KAT2A, KCNAB1, KCNB1, KCNC3, KCND2, KCNH2, KCNIP1, KCNK15, KCNMA1, KCNQ2, KCNQ4, KCTD13, KCTD8, KDM4B, KHSRP, KIAA1279, KIF1B, KLF16, KPNA6, LAMP1, LASP1, LHFPL3, LIG4, LIN7B, LMNB2, LMO4, LONP2, LPIN1, LRCH1, LRFN2, LRPAP1, LRRC7, LRRK2, LTBP2, LYN, MAGI2, MAL2, MANF, MAP1B, MAP4K3, MAP4K4, MAP6, MAPK8IP1, MAPK8IP3, MAST4, MCF2L2, MDGA1, MDH2, MED16, MEIS1, MEIS2, MFI2, MFSD5, MGRN1, MICALL1, MLF2, MLLT1, MMD, MMP2, MPHOSPH6, MSRB2, MT1X, MTHFD1L, MTMR2, MTMR7, MTMR9, MTRR, MTUS2, MXD1, MYCN, MYO1F, MYO5A, MYO5B, NAPA, NAV1, NBEA, NCS1,	657

		NDUFA13, NDUFA7, NDUFAB1, NDUFB2, NDUFB8, NDUFC1, NDUFS7, NEK4, NFIB, NFIX, NGFR, NGFRAP1, NGRN, NIPA1, NKAIN3, NOS2, NPLOC4, NPM1, NPY, NR2E1, NR4A2, NR4A3, NRG1 (includes EG:112400), NSD1, NSF, NTN1, NUMBL, NXPH1, OBSCN, OCIAD1, ODZ3, ODZ4, OLFM1, OLIG1, OPA1, OPA3, OPRD1, OSBPL10, OTOA, OTX2, P2RX4, PACRG, PAH, PALLD, PAN3, PAWR, PAX5, PAX6, PAX7, PCDH9, PCGF3, PCP2, PDE10A, PDE2A, PDE4D, PDE4DIP, PDE8A, PDGFC, PDHX, PDXK, PEBP1, PENK, PEX26, PEX5, PFKP, PFN2, PGAM2, PGK1, PGM3, PHF21B, PHYH, PICK1, PIK3R1, PIP5K1C, PITPNA, PLAUR, PLCD1, PLK2, PLOD2, PLXDC1, PLXDC2, PLXNC1, PMP22, POLG, POLR3A, POLRMT, POMP, PON1, POU3F4, PPM1H, PPP1R14C, PPP2R2B, PPP2R5C, PPP3CB, PRDM2, PRDX2, PRDX6, PRKACA, PRKAR2B, PRKCZ, PRRC2A, PRRT1, PSAP, PSMB8, PSMC6, PSMD1, PSMD13, PTCH1, PTCHD1, PTEN, PTGER2, PTPRG, PTPRM, PTPRN2, PTPRU, PXDNL, RAB11FIP4, RAB28, RAB3A, RAB40B, RAC1, RAI1, RANBP1, RANBP3, RAP1GAP, RAPGEF6, RASGEF1B, RB1, RBL2, RBM10, RBM8A, RBMS1, RECK, RERE, RFX2, RNF220, ROM1, RORA, RPL13, RPL3, RPRD2, RPS19BP1, RPS6KA3, RPS6KA5, RPS6KB1, RRP1B, RTN3, RUNX1, RUSC1, RXRA, S100A1, SACM1L, SBF2, SCAF8, SCOC, SCUBE2, SDK1, SEPHS1, SERF1A/SERF1B, SESN3, SH3KBP1, SHC3, SHC4, SIRT1, SKAP1, SKP2, SLC12A8, SLC17A7, SLC22A5, SLC24A2, SLC25A1, SLC25A16, SLC25A6, SLC26A4, SLC27A1, SLC2A13, SLC35C1, SLC35F1, SLC39A3, SLC9A1, SLC9A3R1, SLCO2A1, SLMAP, SMAD6, SMARCA2, SMOC1, SMU1, SMURF1, SND1, SNTG2, SNX27, SOCS7, SOD1, SORBS2, SORCS2, SORT1, SOX12, SOX9, SP8, SPAG16, SPAST, SPATA2, SPEG, SPG7, SPOCK3, SREBF1, SRF, SRGAP3, SSBP3, ST8SIA3, STK11, STK25, STK39, STRN4, STX2, SUB1, SUCLA2, SUMO3, SUN1, SYN1, SYNGR1, SYNJ2, SYVN1, TAF1A, TAF1C, TAOK2, TARDBP, TBC1D22A, TBCD, TCF7, TGFBRAP1, THOC1, THOP1, THRA, THY1, TLL2, TMEM165, TNFRSF11B, TNFRSF21, TNFRSF6B, TNIK, TOMM20, TPD52L2, TRAPPC9, TRIM44, TRIO, TSC2, TSHZ3, TTC7B, TTC8, TUBA1A, TUBB1, TUBB2B, TUBB4, TULP3, UBAC1, UBB, UNC5B, UNC5C, UPF3B, UQCRC2, USP2, USP36, VAMP2, VAT1L, VAV3, VDAC2, VGLL4, VHL, VIM, WDR37, WNT10A, WWOX, ZBTB16, ZBTB4, ZBTB43, ZCCHC2, ZCCHC24, ZEB2, ZGPAT, ZNF225, ZNF230, ZNF274, ZNF284, ZNF33A, ZNF423, ZNF440/ZNF808, ZNF829	
developmental process of organism	2.77E-17	AARS, ABL1, ACACA, ACADM, ACVR1B, ADAM17, ADAR, ADD1, ADRA2C, AES, AHR, ALG5, ALX3, ALX4, ANKRD11, ANKRD17, APBA2, APLP1, APLP2, AQP11, ARNT, ASB1, ASCL1, ASPH, ATOH1, ATP2B2, ATP5A1, ATP6V0C, ATR, AXIN2, BCAN, BCL10, BCL2L1, BCR, BDNF, BMI1, BMP4, CALML5, CAMK2B, CAPNS1, CARM1, CASP9, CBX2, CD44, CD81, CD9, CDK2AP1, CDK5, CDK5R1, CDX2, CHST11, CHUK, CIT, CLCN2, CLDN1, CLU, COL5A1, COL7A1, COPS3, CRABP1, CREBBP, CRK, CSDA, CSNK1D, CSRNP1, CTBP1, CTBP2, CTHRC1, CTNNA2, CUL1, CUL3, CUX1, CXCL3, CXXC1, CYR61, DCLK1, DEAF1, DGCR2, DHCR7, DLX6, DNMT3A, DOCK1, DOT1L, DSCAML1, DSP, DVL1, DVL2, E4F1, EBF3, ECE2, EDIL3, EDNRA, EFNB2, EGR1, ELAVL1, ELL, EMX1, EMX2, EN1, ENDOG, EOMES, EPAS1, EPHA4, EPHB2, ERCC1, ERCC2, ERF, EZR, FADD, FBN2, FDFT1, FGF19, FGF5, FGF8, FGFR2, FKBP4,	364

		FLI1, FOXE1, FOXF2, FOXK1, FOXP1, FRZB, FZD4, FZD5, GAB1, GAS1, GATA6, GDF5, GDF6, GDF7, GDNF, GFRA2, GGCX, GLI2, GMNN, GNA11, GNAQ, GRHL3, GRSF1, GYS1, GZF1, HBA1/HBA2, HCN4, HGS, HLF, HOXA1, HOXA10, HOXA2, HOXA3, HOXA4, HOXA5, HOXA7, HOXA9, HOXB3, HOXB4, HOXB6, HOXB7, HOXB8, HOXB9, HOXC4, HOXC5, HOXC8, HSF1, ID1, ID2, ID4, IDE, IDH3G, IGF1R, IGFBP1, IHH, IKBKG, IL12A, ILDR2, ING5, INHBB, INSIG1, INSR, INTS1, ITGA1, ITGA5, JAG1, JAG2, JAK3, JAM3, JARID2, JMJD6, JUND, KAT2A, KCNH2, KCNQ4, KEAP1, KISS1, KLF6, LAMB3, LBX1, LGR4, LHX5, LIG3, LIG4, LMO2, LMO4, LMX1A, LMX1B, LTBP4, MAF, MAP1B, MAP1S, MAP3K14, MAP4K4, MAPK8IP3, MATK, MBD3 (includes EG:17192), MDGA1, MDK, MED24, MEIS1, MEIS2, MMP2, MNT, MORF4L1, MXD1, MYCN, MYH6, MYLK2, MYO5A, NAPA, NCOR2, NDEL1, NFATC1, NFIB, NFIC, NGFR, NGFRAP1, NKX2-8, NOS2, NR1H2, NR2E1, NR2F2, NR4A3, NRG1 (includes EG:112400), NSD1, NTN1, NUMBL, ONECUT1, ONECUT2, OPA1, OSR1, OTX2, PALLD, PAX5, PAX6, PAX7, PDE4D, PDGFC, PDPK1, PGM3, PIP5K1C, PKD1, PLEC, PLK2, PLXNA3, PNKP, POFUT1, POU3F4, PPP1CA, PPP2R1A, PPP3CB, PRDX2, PRKACA, PRKAR1B, PRKAR2B, PRKCZ, PRMT1, PSME3, PTCH1, PTEN, PTGER2, PTHLH, PTPN11, RAB3A, RAC1, RANBP3, RB1, RBL2, RBM15, RBP1, RBPJ, RIC8A, RORA, RPS6KA2, RPS6KB1, RXRA, SALL4, SDF4, SEMA3C, SF1, SGPL1, SIM2, SIRT1, SIX3, SKI, SLC22A5, SLC26A4, SLC30A1, SLC35C1, SLIT1, SMAD6, SMAD7, SMARCA4, SMARCC1, SMURF1, SNAI2, SNX27, SOD1, SOST, SOX1, SOX7, SOX9, SP8, SPA17, SPTBN1, SREBF1, SRF, SRSF5, SSBP3, STAM, STK11, STX2, SYVN1, TACC1, TAF4, TARDBP, TBX4, TCF7, TCL1A, TGFBR1, THBD, THRA, TJP1, TLX1, TNFRSF11A, TRIO, TSC2, TSHZ3, TULP3, TWSG1, TXNRD1, TXNRD2, UNC5C, USF2, UTP3, VHL, WHSC2, WNT10A, WNT5A, WNT5B, WNT7B, ZBTB16, ZEB2, ZIC3, ZIC5, ZNF256, ZNF423	
transcription of DNA	3.79E-17	ABCG1, ABL1, ACVR1B, ADCYAP1, AES, AHR, AKAP12, AKAP13, ALX4, APBB2, ARHGEF7, ARNT, ASCL1, ASXL1, ATF5, ATOH1, ATXN3, ATXN7, BAD, BANP, BCL10, BCL2L1, BCR, BDNF, BEX2, BHLHA15, BHLHE22, BLOC1S2, BMI1, BMP4, BSX, CAMTA2, CARM1, CBX2, CBX7, CCNH, CD44, CD9, CDCA7L, CDK9, CDX2, CHD4, CHUK, CIITA, CLU, CREB3L2, CREBBP, CRK, CRLF3, CRTC1, CRY1, CSDA, CSRNP1, CTBP1, CTBP2, CUL1, CUX1, CXCL3, CXXC1, CYTL1, DAB2IP, DBP, DCLK1, DDB1, DDX17, DEAF1, DLX4, DLX6, DNAJB5, DNMT3A, DNTTIP1, DTX3, DUSP1, DUSP3, DVL1, DVL2, E4F1, EAPP, EBF1, EBF3, EGR1, ELL, ELP2, ELP4, EN1, EPAS1, ERCC2, ERF, ESRRG, FADD, FGFR2, FLI1, FOXD4, FOXE1, FOXF2, FOXK1, FOXK2, FOXL1, FOXN3, FOXO3, FOXP1, FUBP1, FXYD1, FZD1, FZD4, FZD5, GADD45A, GATA6, GDF6, GDF7, GDNF, GLI2, GLIS2, GLP1R, GMNN, GNAQ, GNB2L1, GRHL3, GTF2H2/LOC100510744, GZF1, H1F0, HCFC2, HDAC3, HES6, HEY2, HGS, HINFP, HLF, HLTF, HMGN1, HOXA1, HOXA10, HOXA2, HOXA5, HOXA7, HOXA9, HOXB4, HOXB7, HOXB9, HOXC5, HOXC8, HSF1, HSPA8, HTATIP2, HYAL2, ID1, ID2, ID4, IHH, IKBKG, IL12A, ING2, ING5, INHBB, INSM2, INSR, IQGAP1, IRF8, ITGA5, JAG1, JAG2, JAK3, JARID2, JUND, KAT2A, KAT5, KCTD13, KDM5B, KDM6B, KEAP1, KLF10, KLF11, KLF16, KLF6, LBX1, LHX5, LMO2, LMO4, LMX1A, LMX1B, LPIN1, LYN, LZTS1, MAF, MAP2K7, MAP3K10, MAP3K11, MAP3K14, MAP4K4, MAPK13, MAPK8IP1, MAPKAPK2, MBD3 (includes	383

		EG:17192), MCM7, MED16, MED24, MED26, MED27, MED28, MEIS1, MEIS2, MLLT1, MNT, MSRB2, MTA1, MXD1, MYCN, MYST1, MYST2, MZF1, NCOR2, NDUFA13, NFATC1, NFIB, NFIC, NFIX, NKX2-8, NOC2L, NR1D2, NR1H2, NR2E1, NR2F2, NR4A2, NR4A3, NRG1 (includes EG:112400), NSD1, NTN3, OLIG1, ONECUT1, ONECUT2, OTUB1, OTX2, PAWR, PAX5, PAX6, PAX7, PCBD1, PCGF6, PDPK1, PEBP1, PHB2, PIAS4, PIK3R1, PKD1, PLK2, POLR2A, POLR2H, POLR3C, POLRMT, POU3F4, PPAP2B, PPIE, PPP4C, PRDM2, PREB, PRKACA, PRKAR2B, PRKCZ, PRKD1, PRMT2, PRMT5, PRPF6, PSMD14, PTBP1, PTEN, PTGER2, PTHLH, PTPN11, PTPN22, PTPRU, PURA, RAC1, RAI1, RASD1, RASGRP1, RB1, RBL2, RBM14, RBM15, RBPJ, RBX1, REEP5, RFC1, RGS12, RGS20, RORA, RPS6KA3, RPS6KA5, RSF1, RUNX1, RXRA, S100A1, SALL4, SCAF1, SCAF8, SETD8, SIM2, SIRPA, SIRT1, SIX3, SKAP1, SKI, SMAD6, SMAD7, SMARCA2, SMARCA4, SMARCC1, SMURF1, SMURF2, SNAI2, SNAI3, SNAPC2, SND1, SNIP1, SOST, SOX12, SOX7, SOX9, SRCAP, SREBF1, SRF, SS18L1, SSBP3, STAP2, STRAP, SUB1, SUMO1, SUMO3, TAF1A, TAF1C, TAF4, TARDBP, TBKBP1, TBX4, TCF25, TCF7, TEF, TGFBR1, TGFBRAP1, THAP1, THOC1, THRA, TIAL1, TICAM2, TJP1, TLX1, TNFSF13, TOLLIP, TOM1L1, TOPORS, TRIM13, TRIM17, TRRAP, TSC2, TSHZ3, USF2, USP16, USP2, USP22, VAV3, VHL, WHSC1, WNT5A, WWOX, YWHAQ, ZBTB16, ZBTB4, ZBTB7A, ZFP36L2, ZGPAT, ZIC3, ZNF136, ZNF238, ZNF256, ZNF263, ZNF274, ZNF354C, ZNF398, ZNF423, ZNF440/ZNF808, ZNF444, ZNF628, ZXDA	
expression of DNA	1.52E-16	ABCG1, ABL1, ACVR1B, ADCYAP1, AES, AHR, AKAP12, AKAP13, ALX4, APBB2, ARHGEF7, ARNT, ASCL1, ASXL1, ATF5, ATOH1, ATXN3, ATXN7, BAD, BANP, BCL10, BCL2L1, BCR, BDNF, BEX2, BHLHA15, BHLHE22, BLOC1S2, BMI1, BMP4, BSX, CAMTA2, CARM1, CBX2, CBX7, CCNH, CD44, CD9, CDCA7L, CDK9, CDX2, CHD4, CHUK, CIITA, CLU, CREB3L2, CREBBP, CRK, CRLF3, CRTC1, CRY1, CSDA, CSRNP1, CTBP1, CTBP2, CUL1, CUX1, CXCL3, CXXC1, CYTL1, DAB2IP, DBP, DCLK1, DDB1, DDX17, DEAF1, DLX4, DLX6, DNAJB5, DNMT3A, DNTTIP1, DTX3, DUSP1, DUSP3, DVL1, DVL2, E4F1, EAPP, EBF1, EBF3, EGR1, ELL, ELP2, ELP4, EN1, EPAS1, ERCC2, ERF, ESRRG, FADD, FGFR2, FLI1, FOXD4, FOXE1, FOXF2, FOXK1, FOXK2, FOXL1, FOXN3, FOXO3, FOXP1, FUBP1, FXYD1, FZD1, FZD4, FZD5, GADD45A, GATA6, GDF6, GDF7, GDNF, GLI2, GLIS2, GLP1R, GMNN, GNAQ, GNB2L1, GRHL3, GTF2H2/LOC100510744, GZF1, H1F0, HCFC2, HDAC3, HES6, HEY2, HGS, HINFP, HLF, HLTF, HMGN1, HOXA1, HOXA10, HOXA2, HOXA5, HOXA7, HOXA9, HOXB4, HOXB7, HOXB9, HOXC5, HOXC8, HSF1, HSPA8, HTATIP2, HYAL2, ID1, ID2, ID4, IHH, IKBKG, IL12A, ING2, ING5, INHBB, INSM2, INSR, IQGAP1, IRF8, ITGA5, JAG1, JAG2, JAK3, JARID2, JUND, KAT2A, KAT5, KCTD13, KDM5B, KDM6B, KEAP1, KLF10, KLF11, KLF16, KLF6, LBX1, LHX5, LMO2, LMO4, LMX1A, LMX1B, LPIN1, LYN, LZTS1, MAF, MAP2K7, MAP3K10, MAP3K11, MAP3K14, MAP4K4, MAPK13, MAPK8IP1, MAPKAPK2, MBD3 (includes EG:17192), MCM7, MED16, MED24, MED26, MED27, MED28, MEIS1, MEIS2, MLLT1, MNT, MSRB2, MTA1, MXD1, MYCN, MYST1, MYST2, MZF1, NCOR2, NDUFA13, NFATC1, NFIB, NFIC, NFIX, NKX2-8, NOC2L, NR1D2, NR1H2, NR2E1, NR2F2, NR4A2, NR4A3, NRG1 (includes EG:112400), NSD1, NTN3, OLIG1, ONECUT1, ONECUT2, OTUB1, OTX2, PAWR, PAX5, PAX6, PAX7, PCBD1, PCGF6, PDPK1,	384

		PEBP1, PHB2, PIAS4, PIK3R1, PKD1, PLK2, POLR2A, POLR2H, POLR3C, POLRMT, POU3F4, PPAP2B, PPIE, PPP2R5C, PPP4C, PRDM2, PREB, PRKACA, PRKAR2B, PRKCZ, PRKD1, PRMT2, PRMT5, PRPF6, PSMD14, PTBP1, PTEN, PTGER2, PTHLH, PTPN11, PTPN22, PTPRU, PURA, RAC1, RAI1, RASD1, RASGRP1, RB1, RBL2, RBM14, RBM15, RBPJ, RBX1, REEP5, RFC1, RGS12, RGS20, RORA, RPS6KA3, RPS6KA5, RSF1, RUNX1, RXRA, S100A1, SALL4, SCAF1, SCAF8, SETD8, SIM2, SIRPA, SIRT1, SIX3, SKAP1, SKI, SMAD6, SMAD7, SMARCA2, SMARCA4, SMARCC1, SMURF1, SMURF2, SNAI2, SNAI3, SNAPC2, SND1, SNIP1, SOST, SOX12, SOX7, SOX9, SRCAP, SREBF1, SRF, SS18L1, SSBP3, STAP2, STRAP, SUB1, SUMO1, SUMO3, TAF1A, TAF1C, TAF4, TARDBP, TBKBP1, TBX4, TCF25, TCF7, TEF, TGFBR1, TGFBRAP1, THAP1, THOC1, THRA, TIAL1, TICAM2, TJP1, TLX1, TNFSF13, TOLLIP, TOM1L1, TOPORS, TRIM13, TRIM17, TRRAP, TSC2, TSHZ3, USF2, USP16, USP2, USP22, VAV3, VHL, WHSC1, WNT5A, WWOX, YWHAQ, ZBTB16, ZBTB4, ZBTB7A, ZFP36L2, ZGPAT, ZIC3, ZNF136, ZNF238, ZNF256, ZNF263, ZNF274, ZNF354C, ZNF398, ZNF423, ZNF440/ZNF808, ZNF444, ZNF628, ZXDA	
tissue development	1.56E-16	AARS, ABL1, ACADM, ACVR1B, ADAM17, ADAR, ADCYAP1, ADRA2C, AES, AFG3L2, AHR, ALX4, AMIGO1, ANKRD11, ANKRD17, APLP1, APLP2, AQP11, ARHGEF10, ARHGEF7, ARNT, ASB1, ASCL1, ATOH1, ATP2B2, ATP5A1, AXIN2, BAI1, BAIAP2, BCAN, BCL2L1, BCR, BDNF, BMI1, BMP4, CALML5, CAPNS1, CARM1, CARTPT, CASP9, CAST, CBX2, CCK, CD44, CD81, CD9, CDC20, CDK5, CDK5R1, CDX2, CELSR2, CHST11, CHST13, CHUK, CIITA, CIT, CLCN2, CLDN1, CLEC11A, CLU, CNP, CNTNAP1, COL5A1, COL7A1, CREB3L2, CREBBP, CRK, CRLF1, CSDA, CSK, CSRNP1, CSTB, CTBP1, CTBP2, CTHRC1, CTNNA2, CUL1, CUX1, CXCL3, CYR61, CYTH2, DAGLB, DCLK1, DEF6, DGCR2, DHCR7, DLG4, DLX6, DNMT3A, DOCK1, DSCAM, DSCAML1, DSP, DUSP1, DVL1, DVL2, EBF1, EBF3, ECE2, EDIL3, EDNRA, EFNB2, EGR1, ELAVL1, EMX1, EMX2, EN1, ENC1, EOMES, EPAS1, EPHA4, EPHA7, EPHB2, ERCC1, ERCC2, ERF, ESRRG, EZR, FADD, FBXO45, FDFT1, FGF19, FGF8, FGFR2, FKBP4, FLI1, FOXE1, FOXF2, FOXP1, FRZB, FZD1, FZD4, FZD5, GAB1, GALNS, GAS1, GATA6, GDF5, GDF6, GDF7, GDNF, GFRA2, GLI2, GMNN, GNA11, GNAQ, GRHL3, GRSF1, GYS1, GZF1, HEY2, HOXA1, HOXA10, HOXA2, HOXA3, HOXA4, HOXA5, HOXA9, HOXB3, HOXB4, HOXB8, HOXC4, HOXC8, HSF1, ID1, ID2, ID4, IGF1R, IGFBP1, IHH, ILDR2, INHBB, INSIG1, INSR, ITGA1, ITGA5, JAG1, JAG2, JAK3, JAM3, JARID2, JMJD6, JUND, KALRN, KAT2A, KCNA3, KCNH2, KCNQ4, KIAA1598, KISS1, KLF10, KLF6, KREMEN1, KY, LAMB3, LBX1, LGR4, LHX5, LMO4, LMX1A, LMX1B, LRPAP1, LYN, LZTS1, MAF, MAP1B, MAP1S, MAP2K7, MAP3K14, MAP4K4, MAPK8IP3, MATK, MBD3 (includes EG:17192), MDGA1, MDK, MEIS1, MEIS2, MMP2, MTA1, MYCN, MYH6, MYLK2, MYO5A, NAPA, NCKIPSD, NCOR2, NDEL1, NFATC1, NFIB, NFIC, NFIX, NGFR, NKX2-8, NOS2, NPM1, NPR2, NR1H2, NR2E1, NR2F2, NR4A2, NR4A3, NRG1 (includes EG:112400), NTN1, NTN3, NUMBL, ONECUT1, ONECUT2, OSR1, OTX2, PACSIN1, PALLD, PAPPA, PAX5, PAX6, PAX7, PDGFC, PDPK1, PIK3R1, PIP5K1C, PKD1, PLAUR, PLEC, PLXNA3, PMP22, POFUT1, POU3F4, PPP1CA, PPP3CB, PRDX2, PRKACA, PRKAR1B, PRKAR2B, PRKCZ, PRMT1, PTCH1, PTEN, PTGER2, PTHLH, PTPN11, PTPRM, RAB3A, RAC1, RAI1, RB1, RBL2,	364

		RBM15, RBP1, RBPJ, RECK, RORA, RPS6KA2, RPS6KA3, RPS6KB1, RXRA, SALL4, SDF4, SEMA3C, SGPL1, SIM2, SIX3, SKI, SLC11A2, SLC22A5, SLC26A4, SLIT1, SMAD6, SMAD7, SMARCA4, SMARCC1, SMURF1, SNAI2, SOD1, SOST, SOX1, SOX9, SPEG, SPR, SPTBN1, SREBF1, SRF, SRSF5, SS18L1, STAM, STK11, STMN3, STX2, TACC1, TAF4, TBX4, TCF7, TCOF1, TGFBR1, THAP1, THRA, THY1, TLX1, TNFRSF11A, TNFRSF11B, TRIO, TSC2, TSHZ3, TULP3, TWSG1, TXNRD2, ULK1, UNC5C, UTP3, VHL, VSX1, WARS2, WDR5, WHSC1, WNT10A, WNT5A, WNT5B, WNT7B, WWOX, YWHAG, ZBTB16, ZBTB7A, ZEB2, ZIC3, ZIC5, ZNF423	
organogenesis	9.06E-15	AARS, ABL1, ACADM, ACVR1B, ADAM17, ADRA2C, AES, AHR, ALX4, APLP1, APLP2, AQP11, ARNT, ASB1, ASCL1, ATOH1, ATP2B2, AXIN2, BCAN, BCL2L1, BCR, BDNF, BMI1, BMP4, CALML5, CARM1, CASP9, CD44, CDK5, CDK5R1, CDX2, CHUK, CLCN2, CLDN1, CLU, COL5A1, COL7A1, CRK, CSDA, CTBP1, CTBP2, CTHRC1, CTNNA2, CUL1, CUX1, CXCL3, CYR61, DCLK1, DGCR2, DHCR7, DLX6, DNMT3A, DSCAML1, DSP, DVL1, DVL2, EBF3, ECE2, EDNRA, EFNB2, EGR1, EMX1, EMX2, EN1, EOMES, EPAS1, EPHA4, EPHB2, ERCC1, ERCC2, ERF, EZR, FADD, FGF19, FGF8, FGFR2, FKBP4, FLI1, FOXE1, FOXF2, FOXP1, FRZB, FZD4, FZD5, GAB1, GAS1, GATA6, GDF6, GDF7, GDNF, GFRA2, GLI2, GMNN, GNA11, GNAQ, GRHL3, GYS1, GZF1, HOXA1, HOXA3, HOXA5, HOXA9, HOXB3, HOXB4, HOXC4, HSF1, ID1, ID2, ID4, IGF1R, IGFBP1, IHH, ILDR2, INHBB, INSIG1, INSR, ITGA1, ITGA5, JAG1, JAG2, JAK3, JAM3, JARID2, JMJD6, KAT2A, KCNQ4, KISS1, KLF6, LAMB3, LBX1, LGR4, LHX5, LMX1A, LMX1B, MAF, MAP1B, MAP1S, MAP3K14, MAPK8IP3, MDGA1, MDK, MEIS1, MEIS2, MMP2, MYCN, MYH6, MYLK2, MYO5A, NAPA, NCOR2, NDEL1, NFATC1, NFIB, NFIC, NGFR, NKX2-8, NOS2, NR1H2, NR2E1, NR2F2, NR4A3, NRG1 (includes EG:112400), NTN1, NUMBL, ONECUT1, ONECUT2, OSR1, OTX2, PAX5, PAX6, PAX7, PDGFC, PDPK1, PIP5K1C, PKD1, PLEC, PLXNA3, POFUT1, POU3F4, PPP1CA, PPP3CB, PRDX2, PRKAR1B, PRKAR2B, PRKCZ, PTCH1, PTEN, PTGER2, PTHLH, PTPN11, RAB3A, RAC1, RB1, RBL2, RBM15, RBP1, RBPJ, RORA, RPS6KA2, RXRA, SALL4, SDF4, SEMA3C, SGPL1, SIM2, SIX3, SKI, SLC22A5, SLC26A4, SLC35C1, SLIT1, SMAD7, SMARCA4, SMARCC1, SNAI2, SOD1, SOX1, SOX9, SPTBN1, SREBF1, SRF, STK11, STX2, TACC1, TAF4, TBX4, TGFBR1, THRA, TLX1, TNFRSF11A, TRIO, TSC2, TSHZ3, TULP3, TWSG1, TXNRD2, UNC5C, UTP3, VHL, WNT10A, WNT5A, WNT5B, WNT7B, ZEB2, ZIC3, ZIC5, ZNF423	247
activation of DNA	3.92E-14	ABL1, ACVR1B, ADCYAP1, AES, AHR, AKAP12, AKAP13, ALX4, APBA2, APLP1, APLP2, ARHGEF7, ARNT, ASCL1, ASXL1, ATF5, ATOH1, ATXN7, BAD, BANP, BCL10, BCL2L1, BCR, BDNF, BEX2, BHLHA15, BLOC1S2, BMI1, BMP4, BSX, CAMTA2, CARM1, CBX2, CBX7, CCNH, CD44, CD9, CDCA7L, CDK9, CDX2, CHD4, CHUK, CIITA, CLU, CREB3L2, CREBBP, CRK, CRLF3, CRTC1, CRY1, CSDA, CSRNP1, CTBP1, CTBP2, CUL1, CUX1, CXCL3, CYTL1, DAB2IP, DBP, DCLK1, DDB1, DEAF1, DNAJB5, DNMT3A, DNTTIP1, DTX3, DUSP1, DUSP3, DVL1, DVL2, EAPP, EBF1, EGR1, ELL, ELP2, ELP4, EN1, EPAS1, ERCC2, ERF, ESRRG, FADD, FGFR2, FLI1, FOXE1, FOXF2, FOXK1, FOXK2, FOXO3, FOXP1, FUBP1, FZD4, FZD5, GADD45A, GATA6, GDNF, GLI2, GLIS2, GLP1R, GNAQ, GNB2L1,	315

		GTF2H2/LOC100510744, GZF1, HCFC2, HDAC3, HES6, HEY2, HGS, HLF, HLTF, HOXA1, HOXA10, HOXA2, HOXA5, HOXA7, HOXA9, HOXB4, HOXB7, HOXB9, HOXC5, HOXC8, HSF1, HSPA8, HTATIP2, HYAL2, ID1, ID2, ID4, IHH, IKBKG, IL12A, ING2, INHBB, INSR, IQGAP1, IRF8, ITGA5, JAG1, JAG2, JAK3, JARID2, JUND, KAT2A, KAT5, KDM6B, KEAP1, KLF10, KLF16, KLF6, LMO2, LMO4, LMX1A, LMX1B, LPIN1, MAF, MAP2K7, MAP3K14, MAP4K4, MAPK13, MAPK8IP1, MAPKAPK2, MBD3 (includes EG:17192), MED16, MED24, MED26, MED27, MEIS2, MLLT1, MNT, MTA1, MXD1, MYCN, MYST2, MZF1, NCOR2, NDUFA13, NFATC1, NFIB, NFIC, NFIX, NKX2-8, NOC2L, NR1H2, NR2E1, NR2F2, NR4A2, NR4A3, NRG1 (includes EG:112400), NSD1, ONECUT1, ONECUT2, OTUB1, OTX2, PAWR, PAX5, PAX6, PAX7, PCGF6, PDPK1, PEBP1, PHB2, PIAS4, PIK3R1, PLK2, POLR2A, POLR2H, POLR3C, POU3F4, PPAP2B, PPP2R5C, PRKACA, PRKAR2B, PRKCZ, PRKD1, PRMT2, PRMT5, PRPF6, PSMD14, PTBP1, PTEN, PTGER2, PTHLH, PTPN22, PTPRU, PURA, RAC1, RAI1, RB1, RBL2, RBM14, RBM15, RBPJ, RBX1, REEP5, REXO4, RGS12, RGS20, RORA, RPS6KA3, RUNX1, RXRA, S100A1, SALL4, SCAF1, SCAF8, SETD8, SIM2, SIRPA, SIRT1, SIX3, SKAP1, SKI, SMAD6, SMAD7, SMARCA2, SMARCA4, SMARCC1, SMURF1, SMURF2, SNAI2, SNAPC2, SND1, SOST, SOX12, SOX7, SOX9, SRCAP, SREBF1, SRF, SSBP3, STAP2, STRAP, SUB1, SUMO1, SUMO3, TAF1A, TAF1C, TAF4, TARDBP, TBKBP1, TCF25, TCF7, TEF, TGFBR1, THOC1, THRA, TIAL1, TICAM2, TJP1, TLX1, TNFSF13, TOLLIP, TOM1L1, TRIM13, TRRAP, TSC2, TXNRD1, USF2, USP2, VAV3, VHL, WHSC1, WNT5A, WWOX, YWHAQ, ZBTB16, ZBTB4, ZBTB7A, ZIC3, ZNF136, ZNF238, ZNF354C, ZNF423, ZNF444	
development of organ	4.37E-14	AARS, ABL1, ACADM, ACVR1B, ADAM17, ADRA2C, AES, AHR, ALX4, APLP1, APLP2, AQP11, ARNT, ASB1, ASCL1, ATOH1, ATP2B2, BCAN, BCL2L1, BCR, BDNF, BMI1, BMP4, CALML5, CASP9, CD44, CDK5, CDK5R1, CDX2, CHUK, CLCN2, CLDN1, CLU, COL5A1, COL7A1, CRK, CSDA, CTBP1, CTBP2, CTHRC1, CTNNA2, CUL1, CUX1, CXCL3, CYR61, DCLK1, DGCR2, DHCR7, DLX6, DNMT3A, DSCAML1, DSP, DVL1, DVL2, EBF3, ECE2, EDNRA, EFNB2, EGR1, EMX1, EMX2, EN1, EOMES, EPAS1, EPHA4, EPHB2, ERCC1, ERCC2, ERF, EZR, FADD, FGF19, FGF8, FGFR2, FKBP4, FLI1, FOXE1, FOXF2, FOXP1, FRZB, FZD4, FZD5, GAB1, GAS1, GATA6, GDF6, GDF7, GDNF, GFRA2, GLI2, GMNN, GNA11, GNAQ, GRHL3, GYS1, GZF1, HOXA1, HOXA3, HOXA5, HOXA9, HOXB3, HOXB4, HOXC4, HSF1, ID1, ID2, ID4, IGF1R, IGFBP1, IHH, ILDR2, INHBB, INSIG1, INSR, ITGA1, ITGA5, JAG1, JAK3, JAM3, JARID2, JMJD6, KAT2A, KCNQ4, KISS1, KLF6, LAMB3, LBX1, LGR4, LHX5, LMX1A, LMX1B, MAF, MAP1B, MAP1S, MAP3K14, MAPK8IP3, MDGA1, MDK, MEIS1, MEIS2, MMP2, MYCN, MYH6, MYLK2, MYO5A, NAPA, NCOR2, NDEL1, NFATC1, NFIB, NGFR, NKX2-8, NOS2, NR1H2, NR2E1, NR2F2, NR4A3, NRG1 (includes EG:112400), NTN1, NUMBL, ONECUT1, ONECUT2, OSR1, OTX2, PAX5, PAX6, PAX7, PDGFC, PDPK1, PIP5K1C, PKD1, PLEC, PLXNA3, POFUT1, POU3F4, PPP1CA, PPP3CB, PRDX2, PRKAR1B, PRKAR2B, PRKCZ, PTCH1, PTEN, PTGER2, PTHLH, PTPN11, RAB3A, RAC1, RB1, RBM15, RBP1, RBPJ, RORA, RPS6KA2, RXRA, SALL4, SDF4, SEMA3C, SGPL1, SIM2, SIX3, SKI, SLC22A5, SLC26A4, SLIT1, SMAD7, SMARCA4, SMARCC1, SNAI2, SOD1, SOX1, SOX9, SPTBN1, SREBF1, SRF, STK11, STX2, TACC1,	241

		TAF4, TBX4, TGFBR1, THRA, TLX1, TNFRSF11A, TRIO, TSC2, TSHZ3, TULP3, TWSG1, TXNRD2, UNC5C, UTP3, VHL, WNT10A, WNT5A, WNT5B, WNT7B, ZEB2, ZIC3, ZIC5, ZNF423	
modification of protein	5.33E-14	AARS, ABL1, ACVR1B, ADAM17, ADRBK2, AHR, AKT1S1, ALG1, ALG5, ATR, BAG5, BCKDK, BCL10, BCR, BMP4, BRAP, BRSK1, BRSK2, BSG, C13orf15, CAMK1, CAMK2B, CAPN1, CAST, CCK, CCNB1, CCNH, CCT4, CD44, CD81, CD8A, CD9, CDK2AP1, CDK5, CDK5R1, CDK9, CDKL1, CHFR, CHUK, CIT, CLK2, COG3, CRK, CSK, CSNK1D, CTBP1, CTDNEP1, CTDP1, CTDSP2, CUL1, CUL3, DAPK1, DAPK3, DCLK1, DDB1, DNAJA1, DNAJA2, DNAJB1, DNAJB2, DNAJC10, DOLPP1, DPAGT1, DPM2, DUSP1, DUSP11, DUSP3, DUSP6, DVL1, EDIL3, EDNRA, EFNB2, EIF2AK1, EPB41L3, EPHB2, ERCC2, FAM129A, FASTK, FBXO3, FBXO45, FGF5, FKBP1B, FKRP, FLCN, FNDC1, FNTA, FTL, FZD1, GALNT10, GALNT2, GANAB, GGCX, GNB2L1, GPX4, GTF2H2/LOC100510744, HDAC3, HECTD3, HS3ST3B1, HSF1, HSPA8, IGF1R, ING5, INPP5B, INSR, JAK3, KALRN, KCTD13, KLHL9, LIPE, LMTK2, LRPAP1, LRRK2, LTBP4, LYN, MAN1B1, MAP2K7, MAP3K10, MAP3K11, MAP3K14, MAP3K9, MAP4K3, MAP4K4, MAPK13, MAPK4, MAPKAPK2, MAPKAPK5, MATK, MGRN1, MIB2, MKNK2, MSRA, MSRB2, MTMR7, MYLK2, N4BP1, NAA30, NAGPA, NEK4, NNAT, NOS2, NRG1 (includes EG:112400), PDIA6, PDK2, PDPK1, PEBP1, PELI2, PFDN2, PHKG2, PIAS4, PICK1, PIK3R1, PLOD2, PMVK, POFUT1, POLR2A, POLR2H, PPAP2A, PPM1F, PPM1G, PPME1, PPP1CA, PPP2CB, PPP2R1A, PPP3CB, PRDX6, PRKAB1, PRKACA, PRKAR1B, PRKAR2B, PRKCSH, PRKCZ, PRKD1, PRMT1, PRMT2, PSAP, PTCH1, PTEN, PTHLH, PTPN11, PTPN12, PTPN21, PTPN22, PTPRM, PTPRN2, PTPRU, RBX1, RPS6KA2, RPS6KA3, RPS6KA5, RPS6KB1, SBF1, SENP6, SEPHS1, SHB, SIRT1, SIRT3, SKP2, SLC35C1, SMAD7, SMURF1, SMURF2, SOCS7, SOX9, SRPK2, STAP2, STK11, STK25, STK39, SUMO1, SUMO3, SYN1, SYVN1, TBCC, TBCD, TGFBR1, THY1, TNIK, TNNI3K, TOM1L1, TOPORS, TRAF7, TRIM13, TRIM17, TWF1, UBA52, UBE2E1, UBE2G1, ULK1, USP2, USP22, USP42, USP7, VHL, WNK4, WNT5A, ZDHHC17	235
cell division process	1.48E-13	ABL1, ABTB1, ACVR1B, ADCYAP1, AHR, AKAP12, AKAP8, AP2A2, APBB2, ARNT, ASPM, ASXL1, ATF5, ATR, AXIN2, BAD, BANF1, BANP, BCAR1, BCL2L1, BCR, BEX2, BMI1, BMP4, BOD1, BRF1 (includes EG:2972), BRSK1, BRSK2, C13orf15, C1QBP, CABLES1, CAMK1, CAMK2B, CAMK2N1, CAPNS1, CAST, CBX2, CBX7, CCK, CCNB1, CD320, CD44, CDC123, CDC14A, CDC16, CDC20, CDK2AP1, CDK5, CDK5R1, CDK9, CDKL1, CEP76, CETN2, CHFR, CHKA, CHUK, CIRBP, CIT, CKS1B, CLCN2, CLU, CREBBP, CRK, CRLF3, CSNK1D, CUL1, CUL3, CYR61, DDB1, DDX17, DEGS1, DGKZ, DIABLO, DNM1, DNM1L, DSTN, DTD1, DUSP1, DUSP3, E2F8, E4F1, EBNA1BP2, EGFL6, EGR1, ELAVL1, EMX2, EP400, ERCC1, ERCC2, ESRRG, EVL, FADD, FBXO5, FGF8, FGFR2, FLI1, FNTA, FOXK1, FOXN3, FOXO3, FUBP1, FZR1, GAB1, GADD45A, GAS1, GATA6, GDNF, GLI2, GMNN, GNB2L1, GORASP1, GPC1, GPX4, HDAC3, HINFP, HMGN1, HOXA10, HOXA7, HOXB3, HOXB4, HSF1, HSPA8, ID1, ID2, ID4, IGF1R, IKBKG, IL12A, ING2, ING5, INSR, ITGA5, ITGAL, JAK3, JARID2, JUND, KAT5, KDM2A, KDM2B, KDM5B, KLF6, KLHL9, LIG3, LIG4, LSM1, LSM10, LYN, LZTS1, LZTS2, MAP1S, MAP2K7, MAP3K11, MATK, MCM7, MDC1, MDK, MEIS1, MIS12, MNT, MPHOSPH6, MSH6, MUS81, MXD1, MYCN, MYLK2, NCOR2, NDEL1,	275

		NEDD4L, NFATC1, NGFR, NOS2, NPM1, NR2E1, NR4A2, NR4A3, NRG1 (includes EG:112400), NUCKS1, OPA1, PAWR, PAX6, PCYOX1, PDCD6, PDK2, PEBP1, PGAM2, PHF13, PIK3R1, PITPNA, PKD1, PLAUR, PLK2, PNN, POLR2A, PPM1G, PPP1CA, PPP1R15A, PPP2CB, PPP2R1A, PPP2R2B, PPP2R5C, PRKACA, PRKAR2B, PRKCZ, PRMT1, PRMT5, PSAP, PSMD13, PTCH1, PTEN, PTHLH, PTOV1, PTPN11, PTPN22, PTPRN2, PURA, RAB11FIP3, RAB11FIP4, RAC1, RAE1, RANBP1, RASSF5, RB1, RBL2, RBX1, RIC8A, RIOK3, RPS6KA2, RPS6KA3, RPS6KB1, RUNX1, RXRA, SACM1L, SETD8, SIRT1, SKI, SKP2, SLBP, SMAD7, SMARCA2, SMARCA4, SMARCC1, SMC4, SOD1, SOX9, SREBF1, SRF, SRPK2, STK11, STX2, TGFBR1, THAP1, THOC1, TIAL1, TRRAP, TSC2, TUBB1, TUSC2, UBIAD1, UPF1, UQCRFS1, USP16, USP2, USP22, VAV3, VHL, VPS24, VPS4A, WDR6, WWOX, YWHAG, YWHAQ, ZBTB16, ZEB2, ZNF274, ZNF655	
activation of DNA endogenous promoter	4.21E-13	ABL1, ACVR1B, AES, AHR, ALX4, ARNT, ASCL1, ATF5, ATOH1, ATXN7, BDNF, BHLHA15, BLOC1S2, BMI1, BMP4, BSX, CAMTA2, CBX2, CBX7, CCNH, CDX2, CHD4, CIITA, CREBBP, CRK, CRLF3, CRTC1, CSDA, CSRNP1, CTBP1, CUX1, CYTL1, DAB2IP, DBP, DEAF1, DNAJB5, DNMT3A, DVL1, DVL2, EGR1, ELP2, ELP4, EN1, EPAS1, ERCC2, ERF, FADD, FGFR2, FOXE1, FOXF2, FOXK1, FOXK2, FOXO3, FOXP1, FUBP1, FZD5, GATA6, GDNF, GLI2, GLIS2, GLP1R, GNAQ, GTF2H2/LOC100510744, GZF1, HCFC2, HDAC3, HES6, HEY2, HLF, HLTF, HOXA1, HOXA2, HOXA5, HOXA7, HOXB4, HOXB9, HOXC5, HOXC8, HTATIP2, ID1, ID2, ID4, IHH, IRF8, JAK3, JARID2, JUND, KAT2A, KAT5, KDM6B, KLF10, KLF16, KLF6, LMO4, LMX1B, LPIN1, MAF, MBD3 (includes EG:17192), MED16, MED24, MED26, MED27, MEIS2, MLLT1, MNT, MYCN, NCOR2, NFATC1, NFIB, NFIC, NFIX, NKX2-8, NOC2L, NR1H2, NR2E1, NR2F2, NR4A2, NR4A3, ONECUT1, ONECUT2, OTX2, PAWR, PAX5, PAX6, PCGF6, PIAS4, PIK3R1, POLR2A, POLR2H, POLR3C, POU3F4, PRKACA, PRKCZ, PRMT5, PRPF6, PURA, RAC1, RAI1, RB1, RBM14, RBM15, RBPJ, RORA, RPS6KA3, RUNX1, RXRA, S100A1, SALL4, SCAF1, SCAF8, SETD8, SIM2, SIRT1, SIX3, SKAP1, SKI, SMAD7, SMARCA2, SMARCA4, SMARCC1, SNAI2, SNAPC2, SOX7, SOX9, SRCAP, SREBF1, SRF, SSBP3, STRAP, SUB1, TAF1A, TAF1C, TAF4, TARDBP, TCF25, TCF7, TEF, THRA, TIAL1, TLX1, USF2, USP2, VHL, WHSC1, WNT5A, ZBTB16, ZBTB7A, ZIC3, ZNF136, ZNF238	190
differentiation	1.67E-12	ABL1, ACACA, ACADM, ACVR1B, ADAM17, ADCYAP1, ADD1, ADRA2C, AHR, AKT1S1, ALG5, ALOX5, AP3D1, AQP3, ARHGEF7, ARNT, ASCL1, ASPM, ATF5, ATOH1, ATP2B2, AXIN2, BAD, BAIAP2, BCL2L1, BCR, BDNF, BEX2, BHLHE22, BMI1, BMP4, BRSK1, BRSK2, BSG, C1QBP, CAMK1, CAPNS1, CARM1, CARTPT, CAST, CD44, CD81, CD8A, CD9, CDK2AP1, CDK5, CDK5R1, CDK9, CDX2, CHKA, CHUK, CIT, CLEC11A, CLU, CREB3L2, CREBBP, CRK, CSDA, CTBP1, CTBP2, CTNNA2, CTNND2, CUX1, CYR61, CYTL1, DAPK3, DEF6, DHCR7, DLG4, DLK2, DLX6, DNER, DNM1L, DOCK2, DSP, DUSP1, EBF1, EBF3, EGR1, EIF5A, ELAVL1, ELAVL3, EMX1, EMX2, EN1, ENC1, EOMES, EPAS1, EPHA4, EPHB2, ERCC2, ERF, ESRRG, FADD, FAM20C, FGF5, FGF8, FGFR2, FLI1, FOXF2, FOXO3, FOXP1, FRZB, FZD1, FZD5, GAB1, GALNS, GAS1, GATA6, GDF5, GDF6, GDF7, GDNF, GET4, GLI2, GLP1R, GNA11, GNAQ, GPC1, HDAC3, HES6, HEY2, HINFP, HIST1H4A (includes others), HMGN1, HOXA1, HOXA10, HOXA2, HOXA5,	312

		HOXA7, HOXA9, HOXB4, HOXB7, HOXC8, HPS4, ICAM5, ID1, ID2, ID4, IGF1R, IHH, IL12A, ILDR2, INSIG1, INSR, IRF8, ITGA5, ITGAL, ITPKB, JAG1, JAG2, JAK3, JAM3, JMJD6, JUND, KALRN, KCNMA1, KEAP1, KLF10, KLF6, LAMB3, LAMP1, LBX1, LIG4, LMO2, LMO4, LMX1A, LMX1B, LPIN1, LTBP4, LYN, MAF, MAGI2, MAP1B, MAP2K7, MAPK8IP3, MATK, MDK, MED28, MEIS1, METRN, MLLT1, MMP2, MRAP, MTA1, MXD1, MYCN, MYO5A, MYST1, MZF1, NAPA, NCOR2, NFATC1, NFIC, NGFR, NOS2, NPM1, NR2E1, NR4A2, NR4A3, NRG1 (includes EG:112400), NTN1, NTN3, NUMBL, OLIG1, ONECUT1, ONECUT2, OPA1, OSR1, OTX2, PACSIN1, PALLD, PALM, PAX5, PAX6, PAX7, PDE4D, PDGFC, PDPK1, PFN2, PIK3R1, PIK3R2, PIP5K1A, PKD1, PLAUR, PMP22, POLM, PPP1CA, PPP3CB, PRDX2, PRKAR2B, PRKCZ, PRKD1, PTEN, PTGER2, PTHLH, PTPN11, PTPN22, RAC1, RALGDS, RASGRP1, RASSF5, RB1, RBL2, RBM15, RBP1, RBPJ, RND2, RND3, RORA, RPS6KB1, RUNX1, RXRA, SART1, SBF1, SEMA4C, SGPL1, SH3PXD2B, SHC3, SIRT1, SKI, SLC37A4, SLC46A1, SLIT1, SMAD6, SMAD7, SMARCA2, SMARCA4, SMURF1, SOCS7, SORT1, SOST, SOX1, SOX9, SREBF1, SRF, STK11, STK39, STX2, TCF7, TGFBR1, THRA, TLX1, TNFRSF11A, TNFRSF11B, TNFRSF6B, TNFSF13, TOM1L1, TRAPPC9, TSC2, TSHZ3, TUBA1A, TWSG1, TXNRD1, ULK1, UNC5B, VAMP2, VHL, VIM, VSX1, WNT5A, WNT5B, WNT7B, WWOX, YWHAG, YWHAQ, ZBTB16, ZBTB7A, ZIC3, ZIC5, ZNF238, ZNF423	
development of nervous tissue	1.85E-12	ADCYAP1, AFG3L2, AHR, AMIGO1, ARHGEF10, ARHGEF7, ASCL1, ATOH1, BAI1, BAIAP2, BDNF, BMP4, CCK, CD44, CDC20, CDK5, CDK5R1, CELSR2, CIT, CLU, CNP, CNTNAP1, CRLF1, CTNNA2, CYTH2, DAGLB, DCLK1, DLG4, DSCAM, DVL1, EBF3, EN1, ENC1, EPHA4, EPHA7, EPHB2, ERCC2, FBXO45, FGFR2, GALNS, GDNF, GFRA2, GLI2, HEY2, HOXA1, HOXA3, HOXA4, HOXB3, HOXB8, HOXC8, IGF1R, IHH, ITGA1, KALRN, KCNA3, KIAA1598, LMO4, LYN, LZTS1, MAP1B, MAP1S, MAPK8IP3, MYO5A, NDEL1, NFIX, NGFR, NKX2-8, NR2E1, NR4A2, NRG1 (includes EG:112400), NTN1, NTN3, NUMBL, OTX2, PACSIN1, PALLD, PAX5, PAX6, PDGFC, PIP5K1C, PLXNA3, PMP22, PRMT1, PTEN, PTPN11, PTPRM, RAB3A, RAC1, RB1, RBPJ, SKI, SLC11A2, SLIT1, SPR, SRF, SS18L1, STAM, STK11, STMN3, TLX1, ULK1, VSX1, WNT7B, ZIC3	104
skeletal and muscular disorder	5.29E-12	ABCC5, ACADM, ACVR1B, ADAM17, ADARB2, ADCY5, ADRA2C, AIM1, AKAP13, ALDOA, ALOX5, ALX4, ANKFY1, ANKRD11, ANKS1B, AP1S1, AP3B2, AP3D1, APBB2, APLP1, APLP2, ARHGEF10, ARHGEF4, ARHGEF7, ASB3/GPR75-ASB3, ASCL1, ASPH, ASTN2, ATP2A1, ATP2B2, ATP5O, ATP6V1E1, ATP9A, ATRNL1, AUTS2, AXIN2, BAALC, BAD, BAIAP2, BANP, BCAN, BCL2L1, BCR, BCS1L, BDNF, BEX4, BMP4, BRSK1, BSCL2, BSG, BTN2A1, C13orf15, C21orf91, C22orf28, C4orf22, C6orf125, CA11, CA8, CACHD1, CACNA1B, CACNA1C, CACNA2D3, CACNG3, CADPS, CAMK1, CAMK2B, CAMTA1, CAPNS1, CASP9, CAST, CCDC86, CCNB1, CD44, CD81, CD9, CDC16, CDK5, CDK5R1, CDK9, CERKL, CETN2, CHCHD2, CHCHD6, CHRNA4, CHRNB4, CHST11, CIITA, CIRBP, CIT, CLCN2, CLCN7, CLSTN2, CLU, CNP, CNTNAP5, COL5A1, COX5B, CPNE5, CRAMP1L, CREB3L2, CREBBP, CRIM1, CSMD2, CTDP1, CTNNA2, CTNND2, CXCL3, CXXC5, CYB5B, CYB5D2, CYC1, CYP51A1, DAPK1, DBNL, DBP, DCLK1, DCTD, DEAF1, DEDD2, DEF6, DEPDC5, DGCR2, DHCR7, DHX33, DIP2C, DKK2, DNAJA1, DNAJB1,	501

		DNAJB12, DNAJB2, DNER, DNM1, DOPEY2, DPAGT1, DSCAM, DSCAML1, DSP, DTD1, DUSP1, DYX1C1, EBF1, ECHDC1, EDNRA, EEF1A2, EEF1G, EEF2, EGFL8, EGR1, EIF1B, ELOVL6, EML1, EMX2, ENO2, EPAS1, EPDR1, EPHA4, EPHB2, ERCC2, ESRRG, FAM129A, FAM172A, FAM19A5, FBL, FBN2, FDFT1, FGF14, FGFR2, FHOD3, FKBP4, FKRP, FLI1, FOXN3, FOXO3, FOXP1, FRMPD4, FRZB, FTL, FUBP1, FZD5, G0S2, GAD2, GADD45A, GALNT10, GALNT2, GAN, GAS1, GDF5, GDNF, GFRA2, GLE1, GLI2, GLIPR2, GLP1R, GNAL, GNG3, GNG7, GOLIM4, GPC1, GPC6, GPD1, GPR123, GPR6, GPT2, GRHL3, GSTT1, HLTF, HMGN1, HN1L, HNRNPM, HOXC4, HPSE2, HS6ST3, HSPA8, HYAL2, ICAM5, ID4, IDE, IGF1R, IGFBP1, IHH, IKBKG, IL12A, IMPDH1, IMPDH2, INSIG1, INSR, IRF8, ITGA5, ITGAL, ITPKB, JAG2, JAKMIP1, JAM3, JARID2, JPH3, KAT2A, KCNAB1, KCNIP1, KCNMA1, KCNQ2, KCNQ4, KCTD13, KCTD8, KHSRP, KIF1B, KLF16, LAMP1, LBR, LHFPL3, LIPE, LMO2, LMO4, LMX1B, LONP2, LPIN1, LRFN2, LRRC7, LRRK2, LYN, MAGI2, MAL2, MAP1B, MAP3K14, MAP4K3, MAP4K4, MAPK13, MAPK8IP3, MAPKAPK2, MCF2L2, MDC1, MDK, MEIS2, MFI2, MICALL1, MLF2, MLLT1, MMP16, MMP2, MMP28, MRPS27, MRPS6, MSRA, MT1X, MTAP, MTMR2, MTRR, MXD1, MYCN, MYO5A, NAPA, NBEA, NCOR2, NDUFA13, NDUFA7, NDUFB2, NDUFC1, NDUFS7, NEBL, NFIX, NGFR, NGLY1, NGRN, NKAIN3, NOS2, NPLOC4, NPM1, NPR2, NPY, NR4A2, NR4A3, NRG1 (includes EG:112400), NTN1, NUDCD2, ODZ3, ODZ4, OPRD1, OSBPL10, PAPPA, PAX5, PCDH9, PCGF3, PDE10A, PDE4D, PDE4DIP, PDE8A, PDGFC, PDGFRL, PDLIM4, PDXK, PEBP1, PENK, PFN2, PGAM2, PGK1, PGLYRP1, PHB2, PLAUR, PLEC, PLEKHB1, PLK2, PLOD2, PLXDC1, PLXDC2, PLXNC1, PMP22, POLG, PON1, PPM1H, PPP1R7, PPP2R2B, PPP3CB, PRDM2, PRDX2, PRDX6, PRKAR2B, PRKCZ, PRMT1, PRRC2A, PRUNE2, PSMB8, PTCH1, PTEN, PTGER2, PTHLH, PTPLB, PTPN11, PTPN22, PTPRM, PTPRN2, PURA, RAB11FIP4, RAB3A, RAC1, RALGDS, RANBP1, RAP1GAP, RAP1GDS1, RB1, RBM10, RBMS1, RBPJ, RECK, RERE, RHCG, ROM1, RPL13, RPL18A, RPL3, RPS6KA3, RPS6KA5, RPS6KB1, RRP1B, RUNDC3B, RUNX1, RWDD4, SACM1L, SALL4, SBF2, SCOC, SCUBE2, SDCCAG8, SEPHS1, SERF1A/SERF1B, SGCB, SH3KBP1, SHE, SIM2, SIRPA, SIX3, SLC17A7, SLC25A1, SLC25A6, SLC27A1, SLC2A13, SLC41A1, SLC9A3R1, SLIT1, SLMAP, SMAD6, SMG7, SMOC1, SNAI2, SND1, SOD1, SORBS2, SORT1, SOST, SOX9, SP8, SPAG16, SPATA2, SPEG, SPOCK3, SPTBN5, SRPK2, SSBP3, ST8SIA3, STK25, STRN4, SUB1, SUCLA2, SUMO3, SYN1, SYT9, SYVN1, TAF1A, TAF1C, TAOK2, TBC1D22A, TCF7, TCOF1, THRA, THY1, TJP1, TK2, TMEM127, TMEM165, TNFRSF10C, TNFRSF11A, TNFRSF11B, TNFRSF21, TNFRSF6B, TNFSF13, TNIK, TNNI3K, TOMM20, TPD52L2, TRAPPC9, TRIM9, TRRAP, TUBA1A, TUBB1, TUBB2B, TUBB4, TULP3, UBAC1, UBB, UBE2G1, UBE2G2, UNC5B, UNC5C, UQCRC2, USP2, USP36, USP7, VAMP2, VAV3, VIM, WARS2, WNT10A, WNT5A, WNT5B, WNT7B, WWOX, XKR6, XPNPEP3, ZBTB16, ZCCHC24, ZNF230, ZNF440/ZNF808, ZNF767	
differentiation of cells	8.90E-12	ABL1, ACACA, ACADM, ACVR1B, ADAM17, ADCYAP1, ADD1, ADRA2C, AHR, AKT1S1, ALG5, ALOX5, AP3D1, AQP3, ARNT, ASCL1, ASPM, ATF5, ATOH1, ATP2B2, AXIN2, BAD, BAIAP2, BCL2L1, BCR, BDNF, BEX2, BHLHE22, BMI1, BMP4, BRSK1, BRSK2, BSG, C1QBP, CAMK1, CAPNS1, CARM1,	293

		CARTPT, CAST, CD44, CD81, CD8A, CD9, CDK2AP1, CDK5, CDK5R1, CDK9, CDX2, CHKA, CHUK, CIT, CLEC11A, CLU, CREB3L2, CREBBP, CRK, CSDA, CTBP1, CTBP2, CTNNA2, CTNND2, CUX1, CYR61, CYTL1, DAPK3, DEF6, DHCR7, DLK2, DLX6, DNER, DOCK2, DSP, DUSP1, EBF1, EBF3, EGR1, EIF5A, ELAVL1, ELAVL3, EMX1, EMX2, EN1, ENC1, EOMES, EPAS1, EPHA4, ERCC2, ERF, ESRRG, FADD, FAM20C, FGF5, FGF8, FGFR2, FLI1, FOXF2, FOXO3, FOXP1, FRZB, FZD1, FZD5, GAB1, GALNS, GAS1, GATA6, GDF5, GDF6, GDF7, GDNF, GET4, GLI2, GLP1R, GNA11, GNAQ, GPC1, HDAC3, HES6, HEY2, HINFP, HIST1H4A (includes others), HMGN1, HOXA1, HOXA10, HOXA2, HOXA5, HOXA7, HOXA9, HOXB4, HOXB7, HOXC8, HPS4, ID1, ID2, ID4, IGF1R, IHH, IL12A, ILDR2, INSIG1, INSR, IRF8, ITGA5, ITGAL, ITPKB, JAG1, JAG2, JAK3, JAM3, JMJD6, JUND, KALRN, KCNMA1, KEAP1, KLF10, LAMB3, LAMP1, LBX1, LIG4, LMO2, LMO4, LMX1A, LMX1B, LPIN1, LTBP4, LYN, MAF, MAP1B, MAP2K7, MAPK8IP3, MATK, MDK, MED28, MEIS1, METRN, MLLT1, MMP2, MRAP, MXD1, MYCN, MYO5A, MYST1, MZF1, NAPA, NCOR2, NFATC1, NFIC, NGFR, NOS2, NPM1, NR2E1, NR4A2, NR4A3, NRG1 (includes EG:112400), NTN3, OLIG1, ONECUT1, ONECUT2, OSR1, OTX2, PACSIN1, PALLD, PAX5, PAX6, PAX7, PDE4D, PDPK1, PIK3R1, PIK3R2, PKD1, PLAUR, PMP22, POLM, PPP1CA, PPP3CB, PRDX2, PRKAR2B, PRKCZ, PRKD1, PTEN, PTGER2, PTHLH, PTPN11, PTPN22, RAC1, RALGDS, RASGRP1, RASSF5, RB1, RBL2, RBM15, RBP1, RBPJ, RND3, RORA, RPS6KB1, RUNX1, RXRA, SART1, SBF1, SEMA4C, SGPL1, SH3PXD2B, SHC3, SIRT1, SKI, SLC37A4, SLC46A1, SMAD6, SMAD7, SMARCA2, SMARCA4, SMURF1, SOCS7, SORT1, SOST, SOX1, SOX9, SREBF1, SRF, STK11, STK39, STX2, TCF7, TGFBR1, THRA, TLX1, TNFRSF11A, TNFRSF11B, TNFRSF6B, TNFSF13, TOM1L1, TRAPPC9, TSC2, TSHZ3, TUBA1A, TWSG1, TXNRD1, ULK1, VHL, VIM, VSX1, WNT5A, WNT5B, WNT7B, WWOX, YWHAG, YWHAQ, ZBTB16, ZBTB7A, ZIC3, ZIC5, ZNF238, ZNF423	
movement disorder	4.79E-11	ACADM, ADCY5, ADRA2C, AKAP13, ANKRD11, AP1S1, AP3B2, APBB2, APLP1, ARHGEF10, ARHGEF7, ASCL1, ASTN2, ATP2A1, ATP2B2, ATP5O, ATP6V1E1, ATRNL1, ATXN3, ATXN7, BAIAP2, BANP, BCL2L1, BCR, BDNF, BEX4, BRSK1, C21orf91, CA11, CA8, CACNA1C, CACNA2D3, CAMK2B, CAMTA1, CAPNS1, CARTPT, CASP9, CAST, CCDC86, CD44, CD9, CDK5, CDK5R1, CETN2, CHCHD6, CHRNA4, CHRNB4, CIRBP, CIT, CLCN2, CLSTN2, CLU, CNP, CNTNAP1, CNTNAP5, COX5B, CPNE5, CRAMP1L, CRIM1, CSTB, CYC1, CYP51A1, DBNL, DBP, DHCR7, DNAJA1, DNAJB1, DNAJB12, DNAJB2, DNER, DSCAML1, DSP, DYX1C1, EEF1A2, EGR1, EML1, EMX2, ENO2, EPDR1, EPHA4, EPHB2, ESRRG, FAM172A, FBL, FDFT1, FGF14, FGF8, FHOD3, FKBP4, FOXN3, FOXP1, FRMPD4, FTL, FZD4, GAD2, GADD45A, GDNF, GLP1R, GNAL, GNG3, GNG7, GPD1, GPR6, HMGN1, HN1L, HSPA8, ICAM5, ID4, IL12A, ITPKB, JPH3, KCNAB1, KCNC3, KCNIP1, KCNMA1, KCNQ2, KCTD13, KIF1B, KLF16, LAMP1, LONP2, LRRK2, MAL2, MAP1B, MAPK8IP3, MCF2L2, MEIS1, MEIS2, MICALL1, MLF2, MLLT1, MT1X, MYO5A, NAPA, NBEA, NDUFA13, NDUFA7, NDUFB2, NDUFC1, NGRN, NKAIN3, NPLOC4, NPM1, NPY, NR4A2, NRG1 (includes EG:112400), NTN1, OPRD1, OSBPL10, PAX5, PCGF3, PDE10A, PDE4D, PDE4DIP, PDXK, PEBP1, PENK, PFN2, PGAM2, PGK1, PHYH, PLK2, PLOD2, PLXDC1, PLXNC1, PMP22,	230

		PON1, PPM1H, PRDM2, PRDX2, PRDX6, PRKAR2B, PSAP, PSMB8, RAB11FIP4, RAB3A, RANBP1, RAP1GAP, RBM10, RBMS1, RERE, ROM1, RPL13, RPL3, RPS6KA5, RUNX1, SCOC, SCUBE2, SEPHS1, SERF1A/SERF1B, SH3KBP1, SLC17A7, SLC25A1, SLC25A6, SLC27A1, SLC9A3R1, SLMAP, SMAD6, SMOC1, SOD1, SORT1, SOX9, SPATA2, SPOCK3, SSBP3, ST8SIA3, STRN4, SUB1, SUCLA2, SUMO3, SYN1, TAF1C, THY1, TOMM20, TPD52L2, TUBA1A, TUBB2B, TULP3, UBAC1, UBB, UNC5C, USP2, USP36, VAMP2, VAV3, VIM, WWOX, ZBTB16, ZNF423, ZNF440/ZNF808	
development of brain	6.49E-11	ABL1, APLP1, APLP2, ASCL1, ATOH1, ATP2B2, BCAN, BCR, BDNF, BMI1, BMP4, CASP9, CDK5, CDK5R1, CTNNA2, CXCL3, DCLK1, DSCAML1, EBF3, EGR1, EMX1, EMX2, EN1, EOMES, EPHA4, EPHB2, FGF8, FGFR2, GAS1, GDF7, GLI2, HOXA1, ID2, ID4, IGF1R, IGFBP1, KAT2A, LHX5, LMX1A, LMX1B, MAP1B, MAP1S, MAPK8IP3, MDGA1, MYO5A, NAPA, NCOR2, NDEL1, NFIB, NOS2, NR1H2, NR2E1, NR2F2, NR4A3, NRG1 (includes EG:112400), NTN1, NUMBL, OTX2, PAX6, PDPK1, PLXNA3, PTEN, RAC1, RORA, SDF4, SIX3, SKI, SLIT1, SMARCA4, SOX1, SPTBN1, TACC1, TRIO, TULP3, TWSG1, UNC5C, UTP3, WNT5A, ZEB2, ZIC5, ZNF423	81
infection by HIV-1	1.24E-10	ABTB1, AES, AFG3L2, AGAP2, AGBL5, AKAP13, ATG16L2, ATMIN, ATP6V0C, BCR, BICD2, C19orf50, CACNA2D3, CANT1, CCAR1, CD44, CHORDC1, COG3, COL5A1, CRIM1, CTDP1, CYP46A1, DDX50, DEPDC5, DHX33, DNAJA2, DNAJB1, DVL1, EDNRA, ENC1, EPAS1, ERCC1, EXOSC10, EXOSC3, FAM172A, FBXO18, FBXO21, FNTA, GANAB, GBAS, GPT2, GTF2H2/LOC100510744, HDAC11, HDAC3, HGS, IKBKG, KARS, KAT2A, KBTBD7, KIF3C, LSM14B, MAP3K11, MAP3K14, MAP3K9, MED19, MED26, MED27, MED28, MPHOSPH6, MT1X, MUS81, MYO1F, NCOR2, NDUFS7, NGLY1, NIPSNAP3B, NTN1, NUMBL, NUP85, NXF1, OTUD1, PANK3, PDE8A, PDIA6, PGM1, PGRMC2, PIP5K1C, PLOD2, POLR1E, POLR2A, POLR2H, POLR3A, PRPF6, PRPF8, PSMA1, PSMA7, PSMD6, PTPRN2, PTPRU, PURA, RAB1B, RAB28, RAD23A, RANBP1, RBM10, RGPD5 (includes others), RND2, RNF214, RNH1, RPL18, RPS6KA3, RSL1D1, RTN3, S100A1, SBF2, SCAF1, SDF4, SEC14L1, SLC2A13, SLC46A1, SLCO2A1, SLU7, SNRPA1, SPAST, SPG7, SPTBN1, SRSF6, SSU72, ST3GAL3, STXBP1, SUB1, TECR, THAP3, TMED2, TMEM127, TPPP, TRAPPC1, TRAPPC8, TRIM44, TWF1, UBE2E1, UPF3B, UQCRFS1, USP39, VPS4A, ZC3H7B, ZGPAT, ZNF417/ZNF587	138
modification of RNA	3.34E-10	AARS, ADAR, APLP1, ARL6IP4, ATXN3, CCAR1, CCNB1, CD2BP2, CPSF3, CTDP1, DDX17, DDX50, DDX56, DUSP11, EBNA1BP2, EIF4A1, EXOSC3, FASTK, FBL, GRSF1, HNRNPM, HNRNPUL1, INTS1, INTS3, KARS, KHSRP, LSM1, METTL3, NCBP1, NCBP2, NHP2L1, NOS2, NPM1, NPM3, NUDT21, PAPOLA, PARN, PNN, POLR2A, POLR2H, PRPF3, PRPF6, PRPF8, PTBP1, RBM15B, RBM4, RBM8A, RBMS1, RPL14, RPL26, RPS15, RPS7, SARS, SCAF1, SCAF8, SF3A3, SLU7, SNRNP70, SNRPA1, SNRPB, SNRPD2, SNRPE, SOX9, SRPK2, SRSF5, SRSF6, SRSF7, SRSF9, TARDBP, THOC1, TXNL4A, U2AF1, U2AF2, UPF1, UPF3B, USP39, WDR55, ZC3H3	78
disease of central nervous system	3.77E-10	ABCC5, ABL1, ACADM, ADCY5, ADRA2C, AHR, AIM1, ALOX5, ANKRD11, AP1S1, APLP1, ARHGEF7, ASCL1, ASTN2, ATOH1, ATP2A1, ATP2B2, ATP5O, ATXN3, ATXN7, BAIAP2, BANP, BCAN, BCL2L1,	248

		BCR, BCS1L, BDNF, BMI1, BSCL2, CA11, CACNA1B, CACNA1C, CACNA2D3, CAMK2B, CAPNS1, CASP9, CD44, CDCA7L, CDK5, CDK5R1, CETN2, CHRNA4, CHRNB4, CIRBP, CLCN2, CLCN7, CLK2, CLU, CNP, COX5B, CPNE5, CRIM1, CRLF1, CSTB, CXCL3, CYC1, CYP51A1, DBP, DEAF1, DGCR2, DHCR7, DNAJA1, DNAJB1, DNAJB12, DNAJB2, DOCK1, DUSP1, EDNRA, EEF1A2, EGR1, EML1, EMX2, ENC1, ENO2, EOMES, EPHA4, EPHB2, ERCC2, ESRRG, FBL, FDFT1, FGF14, FKBP4, FKRP, FNTA, FOXN3, FOXP1, FRZB, FTL, GAD2, GADD45A, GAS1, GDNF, GLI2, GLP1R, GNAL, GNG3, GNG7, GPD1, GPR6, HMGN1, HOXA9, HSF1, HSPA8, ICAM5, ID2, ID4, IGF1R, IGFBP1, IKBKG, IL12A, IMPDH1, INSR, IRF8, ITGAL, ITM2B, ITPKB, JAG1, JPH3, KAT2A, KCNAB1, KCNC3, KCNIP1, KCNMA1, KCNQ2, KCNQ4, KCTD13, KIAA1279, KLF16, LIG4, LMO4, LYN, MAL2, MAP1B, MAP4K3, MAP4K4, MEIS2, MGRN1, MICALL1, MLF2, MMP2, MT1X, MYCN, MYO5A, NAPA, NBEA, NDUFA13, NDUFA7, NDUFB2, NDUFC1, NDUFS7, NFIB, NFIX, NGFR, NGRN, NIPA1, NOS2, NPM1, NPY, NR4A2, NRG1 (includes EG:112400), NSD1, OBSCN, OLIG1, OPA1, OPA3, OPRD1, OTX2, PAX6, PDE10A, PDE4D, PDE4DIP, PDGFC, PENK, PEX26, PEX5, PFN2, PGAM2, PGK1, PLK2, PLOD2, PMP22, POLG, PON1, PPP2R2B, PPP3CB, PRDX2, PRDX6, PSAP, PSMB8, PTCH1, PTEN, PTGER2, RAB3A, RAC1, RANBP1, RAP1GAP, RB1, RECK, RERE, ROM1, RORA, RPS6KA3, RPS6KA5, SCOC, SCUBE2, SEPHS1, SERF1A/SERF1B, SLC17A7, SLC25A1, SLC27A1, SLC9A1, SLC9A3R1, SLMAP, SMAD6, SOCS7, SORT1, SOX9, SP8, SPAST, SPEG, SPG7, SPOCK3, ST8SIA3, STRN4, SUB1, SUCLA2, SYN1, TCF7, TMEM165, TNFRSF21, TNFRSF6B, TOMM20, TPD52L2, TRIO, TSC2, TUBA1A, TUBB1, TUBB4, UBAC1, UBB, USP2, VAMP2, VAV3, VIM, ZBTB16, ZEB2, ZNF440/ZNF808	
processing of RNA	5.68E-10	AARS, APLP1, ARL6IP4, ATXN3, CCAR1, CCNB1, CD2BP2, CPSF3, CTDP1, DDX17, DDX56, DUSP11, EBNA1BP2, EXOSC3, FASTK, FBL, GRSF1, HNRNPM, HNRNPUL1, INTS1, INTS3, KHSRP, LSM1, NCBP1, NCBP2, NHP2L1, NPM1, NPM3, NUDT21, PAPOLA, PNN, POLR2A, POLR2H, PRPF3, PRPF6, PRPF8, PTBP1, RBM15B, RBM4, RBM8A, RBMS1, RPL14, RPL26, RPS15, RPS7, SARS, SCAF1, SCAF8, SF3A3, SLU7, SNRNP70, SNRPA1, SNRPB, SNRPD2, SNRPE, SOX9, SRPK2, SRSF5, SRSF6, SRSF7, SRSF9, TARDBP, THOC1, TXNL4A, U2AF1, U2AF2, UPF1, UPF3B, USP39, WDR55, ZC3H3	71
developmental process of neurons	6.31E-10	ABL1, ADCYAP1, ADRA2C, AHR, ASCL1, ASPM, ATOH1, ATP2B2, BCL2L1, BDNF, BHLHE22, BMP4, BRSK1, BRSK2, CDK5, CDK5R1, CIT, CNP, CRLF1, CUX1, DAGLB, DAPK3, EBF1, EBF3, ELAVL3, EMX1, EMX2, EN1, ERCC2, FGF5, FGF8, FGFR2, GALNS, GDF5, GDF6, GDF7, GDNF, GET4, GFRA2, GLI2, HEY2, HOXA1, HOXA2, HOXC8, ID2, ID4, IHH, JAG1, KCNA3, KCNMA1, LBX1, LMX1A, LMX1B, MAP1B, MAPK8IP3, MDK, MEIS1, METRN, MRAP, NAPA, NGFR, NR2E1, NR4A2, NRG1 (includes EG:112400), NTN3, OLIG1, OTX2, PACSIN1, PAX5, PAX6, PMP22, POU3F4, RAC1, RB1, RBPJ, RXRA, SHC3, SOX1, SPR, SRF, STAM, THRA, TLX1, ULK1, VHL, VIM, VSX1, WNT5A, WNT7B, YWHAG, ZIC3	91

Supplementary Table 16 - The top between-individual differences in blood and corresponding values in cerebellum and cortex. Values shown represent normalized MeDIP-seq read counts across each specific feature.

			Blood			Cerebellum			Cortex		
**Location**	**Feature**	**Gene**	**Ind 1**	**Ind 2**	**Difference**	**Ind 1**	**Ind 2**	**Difference**	**Ind 1**	**Ind 2**	**Difference**
4:187593970-187594227	Intergenic CGI		5851	8164	-2312	3374	5629	-2255	1884	2796	-912
6:167117435-167117705	Intragenic CGI	RPS6KA2	1788	0	1788	935	0	935	450	0	450
4:187591970-187593970	Intergenic Shore		2409	3978	-1569	1393	2758	-1364	847	1459	-612
2:132732210-132734210	Intergenic Shore		2637	1900	738	1284	716	568	1078	1217	-139
2:3163194-3164420	Intergenic CGI		1379	647	732	499	167	332	613	494	118
4:187594227-187596227	Intergenic Shore		792	1497	-705	460	1002	-541	272	506	-234
7:155640094-155641415	Intergenic CGI		794	108	686	443	26	417	314	73	241
10:1272151-1274151	Intragenic Shore	ADARB2	1383	704	679	401	130	271	193	100	93
8:58283085-58283349	Intergenic CGI		3712	3036	676	1870	1348	522	1286	1153	132
22:48808340-48810340	Intragenic Shore	TTLL8	147	768	-621	62	364	-302	40	211	-171
22:18721886-18722863	Intergenic CGI	AC023490.5	628	1222	-594	292	703	-411	180	360	-180
8:86753787-86753849	Genebody	AC093331.9	1014	462	553	291	124	167	668	506	162
8:58287464-58287728	Intergenic CGI		3391	2884	506	1731	1314	417	1177	1078	99
4:189317842-189318241	Intergenic CGI		1188	703	485	642	272	371	465	365	100
8:918310-918527	Intergenic CGI		568	133	435	195	31	164	219	129	90
8:145252531-145252988	Intergenic CGI		1352	919	432	541	208	333	602	702	-101
21:46168143-46169567	Intragenic CGI	PCBP3	1380	984	395	700	321	380	565	460	104
5:346625-347130	Intragenic CGI	PDCD6	747	356	391	261	77	183	213	129	84
22:17238158-17240158	Intergenic Shore		140	531	-391	68	262	-194	32	103	-71
4:191004198-191004591	Intergenic CGI		1176	791	385	536	216	320	648	780	-133
2:296675-297167	Intergenic CGI		570	185	384	337	68	269	249	132	118
19:41460047-41460465	Intergenic CGI		581	945	-364	148	433	-285	158	311	-153
13:20849293-20849717	Intragenic CGI	ZDHHC20	445	98	347	165	21	144	193	94	99
1:536512-538512	Intergenic Shore		794	452	342	355	269	86	296	194	102
9:139117667-139117869	Intragenic CGI	MAN1B1	702	362	340	311	99	211	284	242	42
19:2298135-2300732	Intragenic CGI	AC004410.1	1317	996	321	645	445	199	458	354	104

21:45590157-45590516	Intergenic CGI		1054	742	312	414	252	162	199	203	-3
12:126811545-126811789	Intergenic CGI		543	243	300	112	55	58	159	47	112
19:2298978-2300978	Intragenic Shore	AC004410.1	1425	1129	296	749	538	211	502	414	88
9:139794946-139795148	Intragenic CGI	EHMT1	669	375	294	311	72	239	276	283	-8
4:8677689-8677958	Intergenic CGI		99	390	-291	17	71	-54	22	124	-102
21:46626893-46629188	Intragenic CGI	PCNT	455	163	291	168	68	101	119	50	69
22:19868849-19870849	Intergenic Shore		204	478	-274	111	260	-150	50	99	-49
4:8683325-8683594	Intergenic CGI		138	400	-262	42	95	-53	30	193	-163
4:8688975-8689244	Intergenic CGI		128	385	-256	42	83	-40	35	205	-170
9:139115667-139117667	Intragenic Shore	MAN1B1	571	320	250	212	85	127	181	174	7
16:22454918-22455163	3'UTR CGI	AC106788.3	352	107	245	79	32	47	167	128	38
3:75800803-75801846	Intergenic CGI		483	240	243	97	56	41	259	213	46
8:58281085-58283085	Intergenic Shore		790	549	242	311	200	111	344	311	32
1:146611213-146611485	Intergenic CGI		380	142	238	105	37	68	136	131	5
20:59398971-59399185	Intragenic CGI	CDH4	1245	1011	233	651	469	182	333	297	36
4:15971514-15971864	Intergenic CGI		554	324	230	265	179	86	214	216	-2
8:58285464-58287464	Intergenic Shore		691	462	229	271	164	107	296	272	24
16:88446575-88446857	Intragenic CGI	SPIRE2	296	69	228	138	0	138	165	62	103

Supplementary Table 17 – Oligo primers used for the bisulfite pyrosequencing verification and replication experiments. [Btn] = biotin tag.

Name	Sequence	CpG sites
BDNF_F	GGTATATTGGGTTAATTTTGGGAAATG	
BDNF_R(Bio)	[Btn]TACCAAAAAACAACCCCCTCTCAT	
BDNF_seq	AGTGTTTATTTTTAGGATTTAGT	5
EOMES_F	GGTAAATATAGAAGGTAGAATAATTAGTGA	
EOMES_R(Bio)	[Btn]ACAATCCTTTAACCATCTCATCT	
EOMES_seq	GTTTTTTTAGATAGATTTAAGAG	8
JMJD2B_F	GTTAGTATTTGTTTGGGTTGTGTTATT	
JMJD2B_R(Bio)	[Btn]CTCTCCAAAAAACCCCAATCT	
JMJD2B_seq	GGTTGTGTTATTTAGAGTGT	7
BC150495_CGI_F(Bio)	[Btn]GGGTTTGAGTAGGTTTGGTTTT	
BC150495_CGI_R	AAACAACCCTCAAACCTAACTAATC	
BC150495_CGI_seq	AACTAATCCCAATCTACATCCC	15
BC150495 SHORE1_F	AGTAAGGAGGAAATAAGTGATATGTTAG	
BC150495_SHORE1_R(Bio)	[Btn]ACCAAAAAAAACTACACCTTCTCTATA	
BC150495_SHORE1_seq	GGTTGTTATGGGGTT	4
BC150495_SHORE2_F	TAGGATTTTGGGAAAGTTATAATAGATGTA	
BC150495_SHORE2_R(Bio)	[Btn]ATCCATTACTTCTCATATACATACATATCT	
BC150495_SHORE2_seq	TGTGAGGGTTTTTATTAGT	3
